# Differential gene expression during recall of behaviorally conditioned immune enhancement in rats: a pilot study

**DOI:** 10.12688/f1000research.123975.1

**Published:** 2022-11-30

**Authors:** Markus Rueckels, Marcus Picard-Mareau

**Affiliations:** 1 Lisa-Kolk-Stiftung, Berg. Neukirchen, North Rhine Westphalia, 51381, Germany

**Keywords:** Behavioral conditioning, NK cells, gene expression analysis, HPA axis, poly I:C, campher smell, Otx2, Sox11, Wnt/β-catenin pathway, Slc18a2, VMAT2, Spp1, OPN, Osteopontin, Gpr88, Fzd6, Zic1, Pmch, Npy, Nps, Chrna3, CAIP, ACTH, IFN-α

## Abstract

**Background:** Behaviorally conditioned immune functions are suggested to be regulated by bidirectional interactions between CNS and peripheral immune system *via* the hypothalamic-pituitary-adrenal (HPA) axis, sympathetic nervous system (SNS), and the parasympathetic nervous system (PNS). Since the current knowledge about biochemical pathways triggering conditioned immune enhancement is limited, the aim of this pilot study was gaining more insights into that.

**Methods:** Rats were conditioned with camphor smell and poly I:C injection, mimicking a viral infection. Following stimulus re-exposure, animals were sacrificed at different time points, and neural tissues along the HPA axis was analyzed with a rat genome array together with plasma protein using Luminex analysis.

**Results:** In the hypothalamus, we observed a strong upregulation of genes related to Wnt/β-catenin signaling (Otx2, Spp1, Fzd6, Zic1), monoaminergic transporter Slc18a2 and opioid-inhibitory G-protein Gpr88 as well as downregulation of dopaminergic receptors, vasoactive intestinal peptide Vip, and pro-melanin-concentrating hormone Pmch. In the pituitary, we recognized mostly upregulation of steroid synthesis in combination with GABAergic, cholinergic and opioid related neurotransmission, in adrenal glands, altered genes showed a pattern of activated metabolism plus upregulation of adrenoceptors Adrb3 and Adra1a. Data obtained from spleen showed a strong upregulation of immunomodulatory genes, chemo-/cytokines and glutamatergic/cholinergic neurotransmission related genes, as also confirmed by increased chemokine and ACTH levels in plasma.

**Conclusions:** Our data indicate that in addition to the classic HPA axis, there could be additional pathways as e.g. the cholinergic anti-inflammatory pathway (CAIP), connecting brain and immune system, modulating and finetuning communication between brain and immune system.

## Introduction

Pavlovian or classical conditioning is a phenomenon of associative learning, in which a behavioral response is induced by establishing a temporal pairing between a conditioned stimulus (CS) and unconditioned stimulus (US). ^1^ In addition to the well-known phenomenon of classic conditioning of physiological reflexes, immune responses can also be memorized and recalled using Pavlovian conditioning, based on a reciprocal communication between the central nervous system (CNS) and the peripheral immune system. ^2^

This concept of behaviorally conditioned immune modulation was rediscovered by Ader and Cohen ^3^ in the 1970s, who used cyclophosphamide as an immunosuppressive agent (US) and illness-induced taste aversion by lithium chloride (LiCl) solution as CS and then measured the changes in immune responsiveness by a specific antibody reaction, using sheep erythrocytes as antigen. Since the study was published, this phenomenon of conditioned immunosuppression has been replicated in a multitude of studies ^4^^–^^7^ by different researchers all over the world and current knowledge about this phenomenon including potential pathways at work and experimental paradigms employed have been comprehensively summarized in a recent review by Hadamitzky *et al.* ^8^

While most of our knowledge on conditioning of immunological functions is derived from immunosuppression paradigms, relatively few studies have been focusing on immune enhancement. A series of these studies targeted the conditioned enhancement of both CTLs and natural killer (NK) cells in rodents, identifying multiple possible signaling molecules and pathways driving these effects. ^9^^–^^12^

Historically, behaviorally conditioned immune functions are suggested to be regulated by bidirectional interaction between CNS and peripheral immune system via the hypothalamic-pituitary-adrenal (HPA) axis, sympathetic nervous system (SNS), and the parasympathetic nervous system (PNS). ^13^ In addition to the HPA axis, the brain limbic system (cortex, hippocampus, amygdala) has also been associated with behaviorally conditioned immune responses. ^13^ In a previous study, ^11^ both adrenocorticotropic hormone (ACTH) and interferon-alpha (IFN-α) were shown to be involved in the efferent signaling pathways during recall of the conditioned enhancement of NK cell activity. ^11^ However, the exact biological basis (biochemical, neuroanatomical, genetic) and the underlying molecular mechanisms driving these processes are still unclear.

To shed more light on these pathways, we conducted a pilot study, using the formerly published 3-day conditioning paradigm originally developed by Hsueh *et al.*. ^11^ Following that paradigm, animals were first exposed to camphor smell and then injected with polyinosinic:polycytidylic acid (poly I:C), simulating a viral infection and also inducing so-called sickness behavior, an animal model for depression-like behavior in rodents. ^14^^,^^15^ 48h later, animals in the test group were re-exposed to camphor smell, only, to recall a behaviorally conditioned immune response in rats while animals in a positive control group receive a re-injection of poly I:C and animals in a negative control group receive smell conditioning, only, but no poly I:C re-injection.

We then used a gene-agnostic expression analysis approach to identify first gene candidates along the 4 major tissues – hypothalamus, pituitary gland, adrenal glands, and spleen - of the HPA axis while in parallel looking at cytokine/chemokine protein levels in the plasma of the conditioned animals. Results from both approaches were combined and aligned with the existing literature to provide a list of candidate genes and proteins for future studies.

## Methods

### Animals

Male, Sprague Dawley (SD) rats (5-7 weeks, 160-250 g) were obtained from InVivos Pte Ltd, Singapore. Male animals were chosen to minimize the impact of hormonal cyclical changes on gene expression and behavioral readouts. Animals were housed at the Biological Resource Centre (BRC) in groups of 2 in individually ventilated cages and maintained on a 12h light/dark cycle with food and water *ad libitum* in accordance with the Agency for Science, Technology and Research (A*STAR) Animal Care and Use Committee. All efforts were made to ameliorate any suffering of animals; animals were provided with Nylabones (hard non-toxic nylon), nestlets and/or domes for environmental enrichment to ensure adequate welfare and psychological well-being. In accordance with IACUC guidelines, animals were allowed to acclimatize for a minimum of 3 days prior to study commencement and examined to confirm their health status.

### General conditioning procedure and tissue collection

Animals (N=30) were randomly allocated into two series (a and b) of 15 animals, each, and divided into three experimental groups as shown in Table 1. A 3-day conditioning paradigm was used in the study as described previously ^11^ where camphor smell was used as conditioned stimulus (CS) and poly I:C injection (ip) as unconditioned stimulus (US). In the present study, camphor smell exposure was performed in a separate procedure room for 1h using a 10 cm petri dishes filled with 5 g of camphor powder (Sigma #21310) placed above the cage (on top of the metal grid) and warmed up with a heat lamp. On day D0, animals from all the three groups were exposed to camphor smell for 1h following exposure to saline (ip; 10 ml/kg; negative control group) and poly I:C (Sigma #P1530; ip; 1 mg/kg; test and positive control group). On day D2, animals from test and negative control groups received saline injections (ip), following exposure to camphor smell for 1h whereas animals from positive control group were exposed to poly I:C only. After the last injection on day D2, animals from each group were sacrificed at 0h (n=2), +3h (n=2), +6h (n=2), +24h (n=2) and +48h (n=2) post injection, and tissues were collected (adrenal gland, hypothalamus, pituitary gland, and spleen) and stored snap frozen in RNAlater (MilliporeSigma, Burlington, U.S.A) for further analysis. Blood was collected for the isolation of peripheral blood mononuclear cells (PBMCs) and serum to perform cytokine/chemokine array analysis. Animal experiments were conducted at Biological Resource Centre (BRC), Biomedical Sciences Institutes, Agency for Science, Technology and Research (A*STAR), 20 Biopolis Way, #07-01 Centros Building, Singapore 138668.

**Table 1.** Animal groups and conditioning protocol.

Group number	Group name	1 ^st^ treatment (Day D0)	2 ^nd^ treatment (Day D2)
1	Test (n=5)	Camphor smell + Poly I:C	Camphor smell + Saline
2	Positive control (n=5)	Camphor smell + Poly I:C	Poly I:C
3	Negative control (n=5)	Camphor smell + Saline	Camphor smell + Saline

### Sucrose preference test (SPT)

The SPT is a standard behavioral test to assess depression-like behavior in rodents during the development of anti-depressive treatments. The SPT is based on anhedonia (lack of interest in rewarding stimuli), which is present in some forms of affective disorders including depression. In this test, the interest of animals in seeking out a sweet rewarding drink relative to plain drinking water is used and a bias toward the sweetened drink is typical, failure to do so is an indication of anhedonia/depression. ^16^ The SPT was carried out on days -7 to - 1 (run-in phase for baseline data) as well as days D0 - D3. Rats were presented with two drinking bottles, one for each plain drinking water and 2% (w/v) sucrose solution for 24 h. The ratio of the sweetened and plain water consumed was calculated, and results were expressed as %sucrose preference.

### Affymetrix microarray analysis

At the end of the study, mRNA from all tissues harvested at different time points was isolated by using a RNeasy Plus minikit (QIAGEN, Hilden, Germany). RNA quality was assessed by using a Bioanalyzer 2100 (Agilent Technologies, Santa Clara, U.S.A.). Highly purified RNA was subjected to microarray analysis with a GeneChip ^TM^ Rat Gene 2.0 ST Array (Affymetrix, Inc, Santa Clara, U.S.A.) to identify differential gene expression during the recall of behaviorally conditioned immune responses. RNA labelling, hybridization, staining, and scanning were performed according to the manufacturer’s instructions. Briefly, 100 ng of total RNA from each sample was reverse transcribed to cDNA, followed by overnight in vitro transcription to generate cRNA which was further fragmented and labeled. The quality of cDNA and fragmented cRNA was assessed with an Agilent bioanalyzer. We used the Robust Multichip Average (RMA) model for array background correction and quantile normalization as described previously. ^17^^–^^19^ Probes were mapped to the Rattus norvegicus genome chip (RGSC 5.0/rn5) and results from all tissues were normalized using beta-Actin.

### Analysis of differentially expressed genes

All gene expression results of samples in test and positive control were matched by tissue and time point with their respective counterpart in negative control and subtracted to derive single point gene expression differences (all data expressed as 2^x). Due to insufficient RNA quality after extended storage, we could not analyze all tissue samples by microarray and had to discard multiple samples (hypothalamus negative control +24h, pituitary test and positive control 0h and +3h, adrenal test +6h and +24h and positive control 0h and +3h, spleen +24h and +48h all samples); accordingly, we first limited the analysis to the average across all available time points and then looked at single point regulations with focus on neurotransmitter-related genes in more detail; in each sample, a minimum of three individual time points were available. Following gene enrichment and comparison of test vs. negative and positive vs. negative control selection, all differentially expressed genes per tissue were then further analyzed by Reactome pathway analysis. ^20^ As we hypothesized the origin of the message post recall was from hypothalamus, we additionally leveraged QIAGEN Ingenuity Pathway Analysis (QIAGEN IPA) for a more detailed analysis of hypothalamic genes. All genes identified with avg. regulation >2fold in the hypothalamus of test animals were selected and subcellular network analysis was derived using IPA graphic interface.

### Quantitative RT-PCR

The same RNA of all samples as used in the microarray analysis was also subjected to quantitative reverse transcription PCR (qRT-PCR) to confirm the observed differential gene expression. The qPCR laboratory work was conducted as a fee-for-service by the Qiagen service laboratory (Qiagen Life Science Service & Support, Hilden, Germany), using the RT ^2^ Profiler ^TM^ PCR custom Array RAT (CLAR27943). Briefly, RNA was isolated, and cDNA was synthesized with Qiagen RT ^2^ First Strand Kit using 500 ng of RNA, 1 μl RT primer mix (oligo-dT and random hexamer primers), 4 μl Quantscript RT buffer (5X) and 1 μl Quantiscript Reverse Transcriptase (QuantiTect kit, Cat. No. /ID: 205311, Qiagen). qPCR reactions were then assembled using synthesized cDNA (1μl), 5 μl RT ^2^ Profiler PCR Array SYBR Green master mix (Qiagen, Germany), 1μl of each primer diluted to a 5 μM working solution and 1μl sterile water and processed in 96-well format including housekeeping genes, RNA and DNA controls, using Qiagen’s proprietary primer panel. The mRNA expression was determined using the 2− ^ΔΔCT^ method. ^21^ All gene expression values were normalized using Hypoxanthine phosphoribosyl transferase (HPRT), glyceraldehyde phosphate dehydrogenase (GAPDH) and β-Actin as references for expression analysis of genes of interest.

### Cytokine array analysis

Plasma protein levels for all animals and time points were evaluated as a fee-for-service by Singapore Immunology Network (SIgN), coordinated by Biomedical Research Council (#04-06 Immunos, Singapore), using the MILLIPLEX ^®^ MAP rat pituitary endocrine multiplex assay (RPTMAG-86K) for ACTH and BDNF and MILLIPLEX ^®^ MAP Cytokine/Chemokine Panel (RECYMAG65K27PMX, both MerckMillipore, Darmstadt, Germany) to simultaneously analyze the levels of 27 further cytokines/chemokines. Plasma samples were diluted to 2 mg/ml protein concentration, then analyzed on a MAGPIX system (Luminex, Austin, U.S.A.) and quantified using MILLIPLEX ^®^ Analyst 5.1 software. Cytokines/chemokine measured included RANTES, GRO KC CINC-1, VEGF, Fraktalkine, LIX, MIP-2, G-CSF, Eotaxin/CCL11, GM-CSF, IL-1α, Leptin, MIP-1α, IL-4, IL-5, IL-1β, IL-2, IL-6, EGF, IL-13, IL-10, IL-12p70, IL-5, IL-17A, IL-18, MCP-1, IP-10, KC, VEGF, Fractalkine, LIX, MIP-2 and TNF-α.

### Statistical analyses

All statistical analysis was conducted using RMA gene expression data for all available time points for test, positive and negative control, respectively, and applying a two-tailed, unpaired, homoscedastic Student t-test (Microsoft ^®^ Excel ^®^ for Microsoft 365 MSO, version 2202). A *p*-value of <0.05 was considered significant.

### Ethical approval

This study was approved under the Institutional Animal Care and Use Committee (IACUC) protocol number BRC IACUC #151001 by the IACUC of the Biological Resource Centre (BRC, Biomedical Sciences Institutes, Agency for Science, Technology and Research (A*STAR), 20 Biopolis Way, #07-01 Centros Building, Singapore 138668) on 26 Feb 2015. A specific procedure amendment pertaining to the use of sucrose preference test was approved on 03 Feb 2016.

## Results

### Gene expression analysis

The present pilot study was designed to identify differentially expressed genes along the HPA-axis i.e., hypothalamus, pituitary, adrenal, and spleen during recall of the behaviorally conditioned immune response, using a conditioning paradigm to explore efferent pathways. ^11^ As we didn’t know the exact time point of gene alteration, we first looked at genes with maximum average regulation across all time points (0h, +3h, +6h, +24h, and +48h post recall) in all three studied groups and then looked at neurotransmitter-related regulations in more detail, also considering single point regulations. Each analysis per group and tissue included a minimum of three independent timepoints (for exact samples analyzed, see Methods).

**Figure 1.**
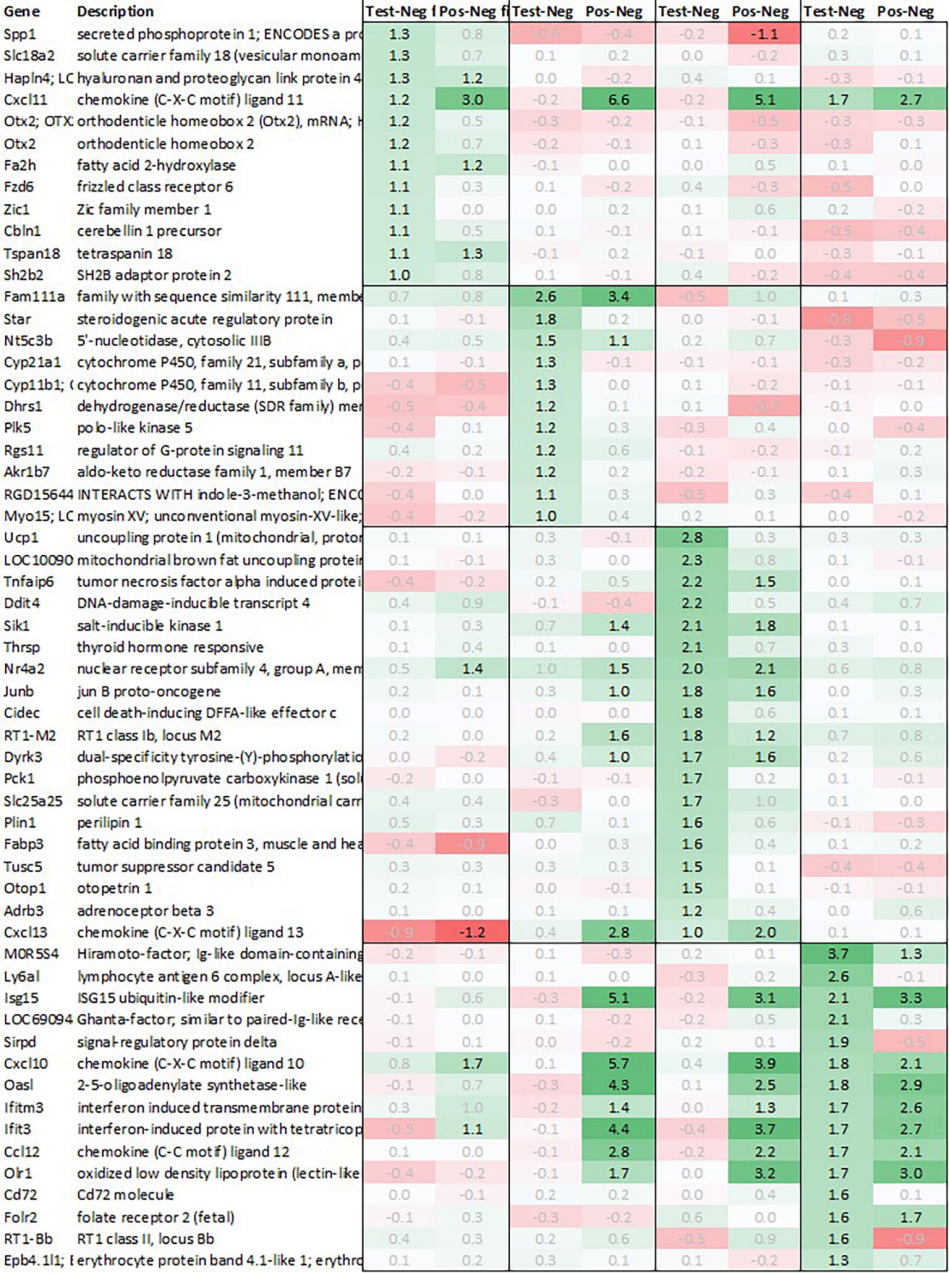
Genes upregulated across all four tissues; differential gene expression shown as avg. across all time points test vs. negative and positive vs. negative controls.

**Figure 2.**
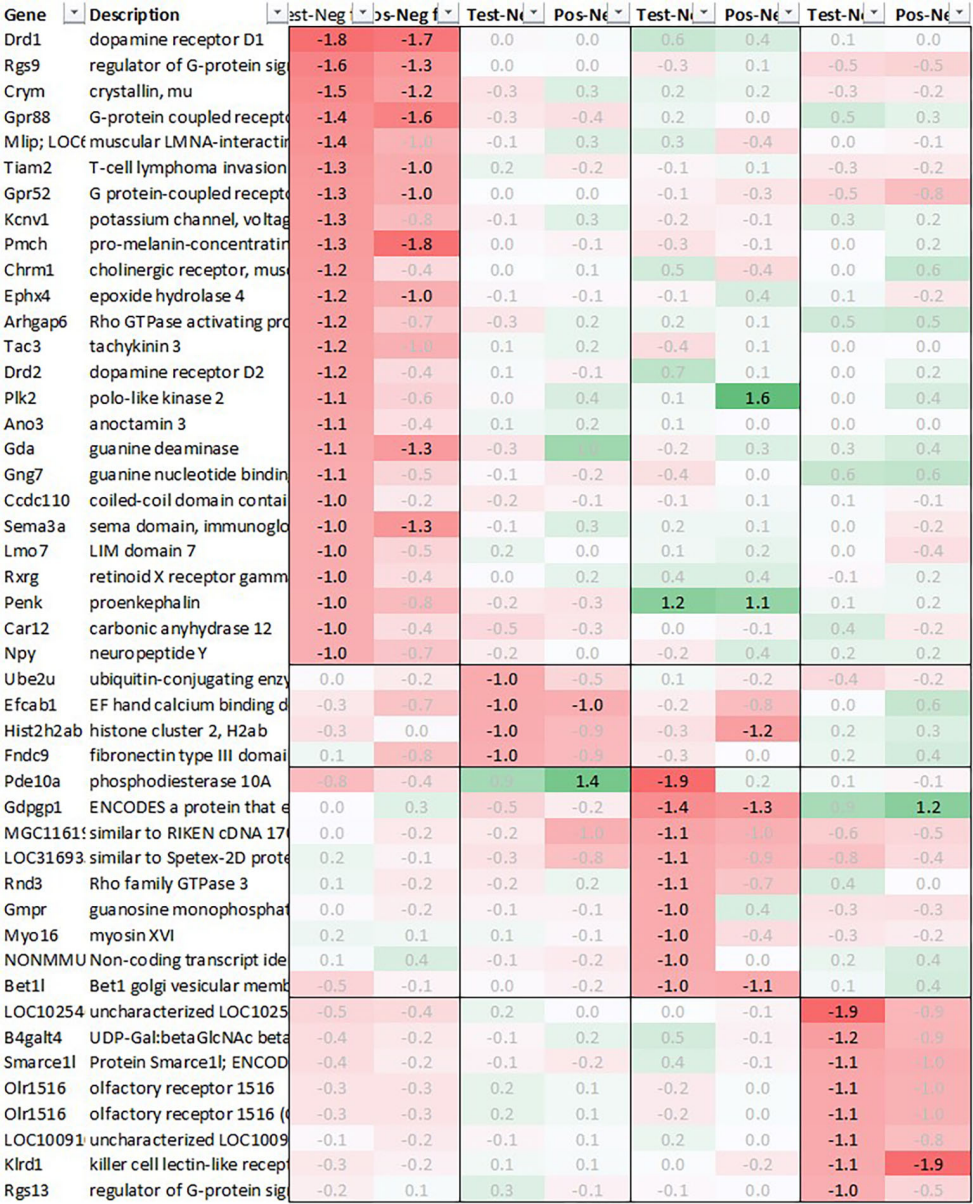
Genes downregulated across all four tissues; differential gene expression shown as avg. across all time points test vs. negative and positive vs. negative controls.

Leveraging this approach to identify major upregulated genes in test vs. negative control (‘test’) across all tissues with a >2fold average regulation across all time points, we identified 12 and 10 upregulated transcripts in hypothalamus and pituitary, respectively ( Figure 1). In adrenal and spleen, however, the number of genes upregulated using this approach was significantly higher. To identify meaningful candidates in these tissues, we therefore excluded transcripts with unknown identity and focused only on genes with strong upregulation during the first 6h post recall and/or *p*<0.05, comparing expression across all time points in test with their respective counterpart in negative control, thereby reducing the number of transcripts in adrenal glands and spleen to 18 and 20, respectively. We also excluded genes with a stronger upregulation in positive vs. negative control (‘positive’) than test except when also showing a strong regulation across multiple other tissues as e.g. Cxcl11 or Cxcl13 and applied a similar approach to focus on the most promising candidate genes for downregulation ( Figure 2).

In addition to this, we focused at up- and downregulation of neurotransmitter-associated genes such as receptors, transporters, and solute carriers, potentially involved in the efferent signaling post recall such as dopaminergic, serotonergic, opioid, cholinergic, glutamatergic, and GABAergic neurotransmission, selecting for strong regulation across multiple tissues even if not crossing an average >2fold regulation across all timepoints and limiting the time frame to the first 6h post recall unless otherwise mentioned.

### Hypothalamus

In the hypothalamus, a total of 11 transcripts, detected by 12 probes, were upregulated >2fold when comparing average regulation across all time points test vs. negative. Out of these, Slc18a2 coding for vesicular monoamine transporter 2 (VMAT2) was found upregulated significantly test vs. negative, while both Otx2, coding for orthodenticle homeobox 2 and detected by two independent probes, and Spp1, coding for secreted immune modulator osteopontin (OPN), despite strong regulation only showed a trend towards significance when combining all timepoint test vs. negative control ( Figure 1).

8 additional genes - Hapln4, Cxcl11, Fa2h, Fzd6, Zic1, Cbln1, Tspan18, and Sh2b2 - were also found upregulated >2fold in test however either were also strongly upregulated in positive or did not reach significance when comparing gene expression in test vs. negative control: while Fzd6 and Zic1 were mainly upregulated in test vs. negative, Hapln4, Cxcl11, Fa2h, Tspan18, and Sh2b2 showed a similar or even higher differential gene expression in positive vs. negative than test vs. negative.

As both Otx2 and Spp1 have been shown to interact with Sox9 (SRY box transcription factor 9) ^22^and furthermore, like Fzd6 and Zic1, seem to be involved in Wnt/ß-catenin signaling, we also analyzed Sox-related gene expression and found that Sox7/8/9/10/11 and 13 were also upregulated in hypothalamus test vs. negative control, with Sox11 showing the strongest immediate regulation post recall ( Figure 4).

Looking at downregulation ( Figure 2), among the genes with the strongest average regulation across all time points were dopamine receptor Drd1 and Drd2, inhibitory regulatory G-protein Rgs9, and Gpr88, involved in negative regulation of opioid signaling as well as thyroid hormone-binding protein crystallin-mu (Crym). Likewise, Gpr52, shown to have a modulatory role in dopamine receptor signaling, as well as Mlip, Tiam2, Kcnv1, Chrm1, Ephx4, Arhgap6, Tac3, Plk2, and Ano3 were strongly downregulated.

Looking in more detail at neurotransmitter-related regulation ( Figure 3a), we detected an upregulation of inhibitory glycine- (Glra1, Slc6a9, Slc6a5) and GABA- (Slc6a11, Gabra6) plus excitatory glutamate-related genes (Grik3, Grik4, Grm1, Grm4, Grin2d and 3b, Slc17a6 and Slc25a18). In addition, we saw an upregulation of neuropeptide S (Nps), adenylate cyclase activating polypeptide 1 (Adcyap1) plus an upregulation of nicotinic cholinergic (Chrna4, Chrna2, and Chrna6) and opioid genes (Pnoc, Pcsk1n; detected by two independent probes). Interestingly, while this upregulation was observed with multiple genes immediately post recall in positive, the same upregulation was only observed +3h post recall in test.

**Figure 3a.**
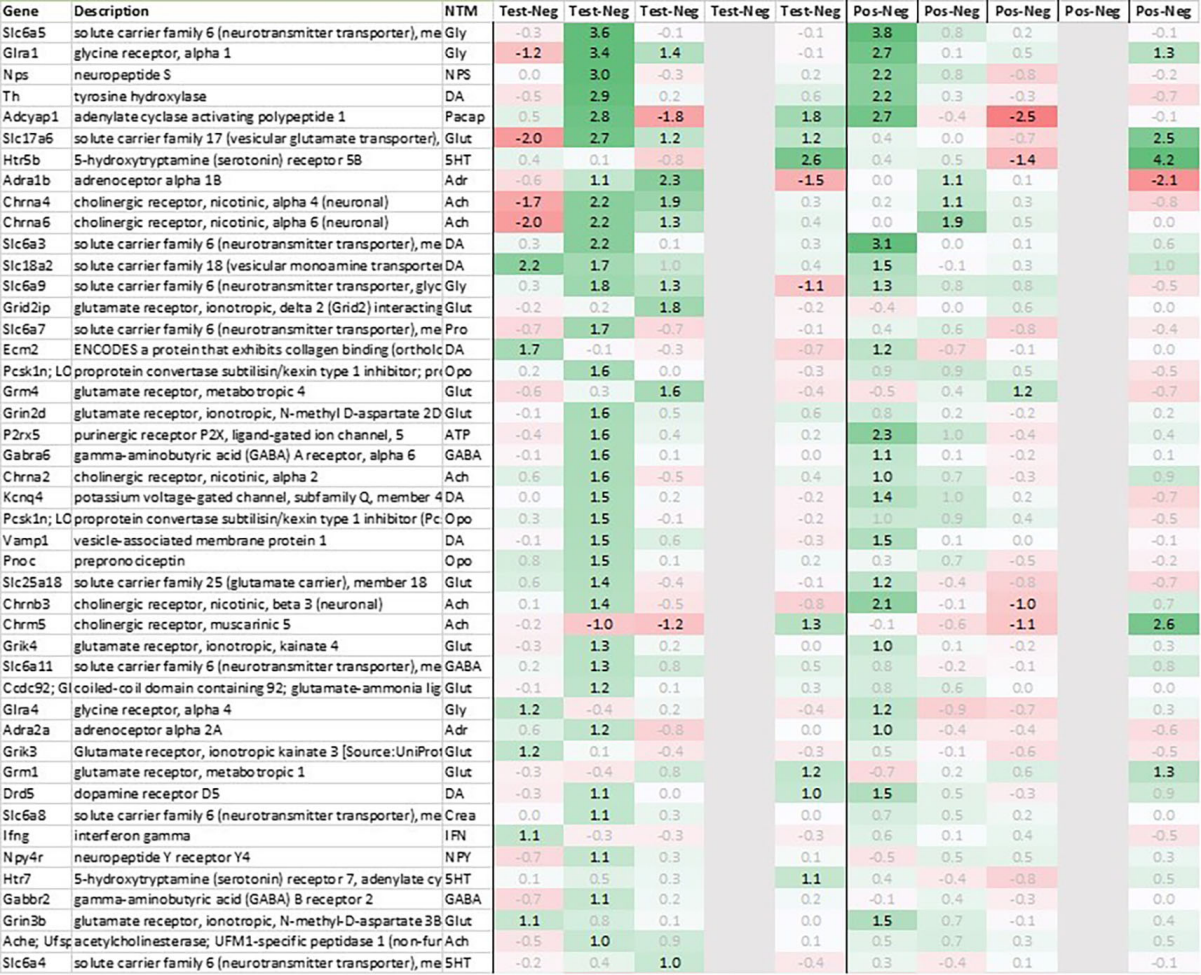
Neurotransmitter-related genes upregulated in the hypothalamus (average regulation test vs. negative and individual time points 0h, +3h, +6h, +24h, and +48h post recall; test vs. negative and positive vs. negative control, respectively). All data shown as 2^x.

This pattern of +3h delayed regulation in test vs. positive was also seen with many downregulated signaling-related transcripts ( Figure 3b) as enkephalin precursor proenkephalin Penk. We also observed a downregulation of Pcsk1 and prodynorphin Pdyn, opioid receptors kappa and mu (Oprk1, Oprm1), GABAergic- (Gabra4, Gabra5, Gabrad, Gabrarq), serotonergic- (Htr2a, Htr1d, and Htr6) and muscarinic cholinergic receptors (Chrm1), opioid regulators Rgs4 and 9, and neuropeptide Y (Npy; detected by two independent probes). Interestingly, two of the most pronounced regulated genes - pro-melanin-concentrating-hormone Pmch and vasoactive intestinal peptide Vip - were downregulated in the hypothalamus of test animals immediately post recall by more than 30fold.

**Figure 3b.**
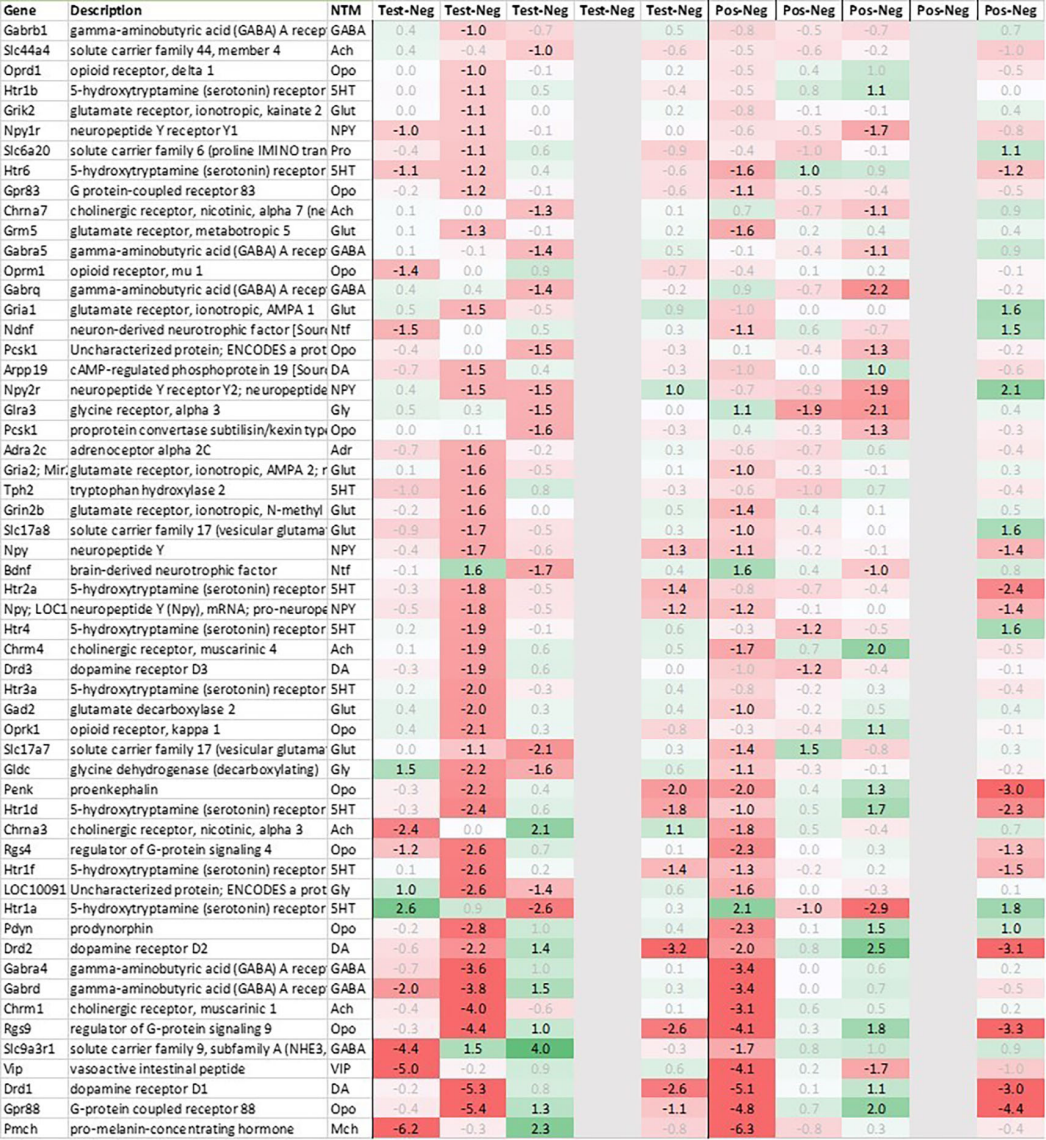
Neurotransmitter-related genes downregulated in the hypothalamus (average regulation test vs. negative and individual time points 0h, +3h, +6h, +24h, and +48h post recall; test vs. negative and positive vs. negative control, respectively). All data shown as 2^x.

**Figure 4.**
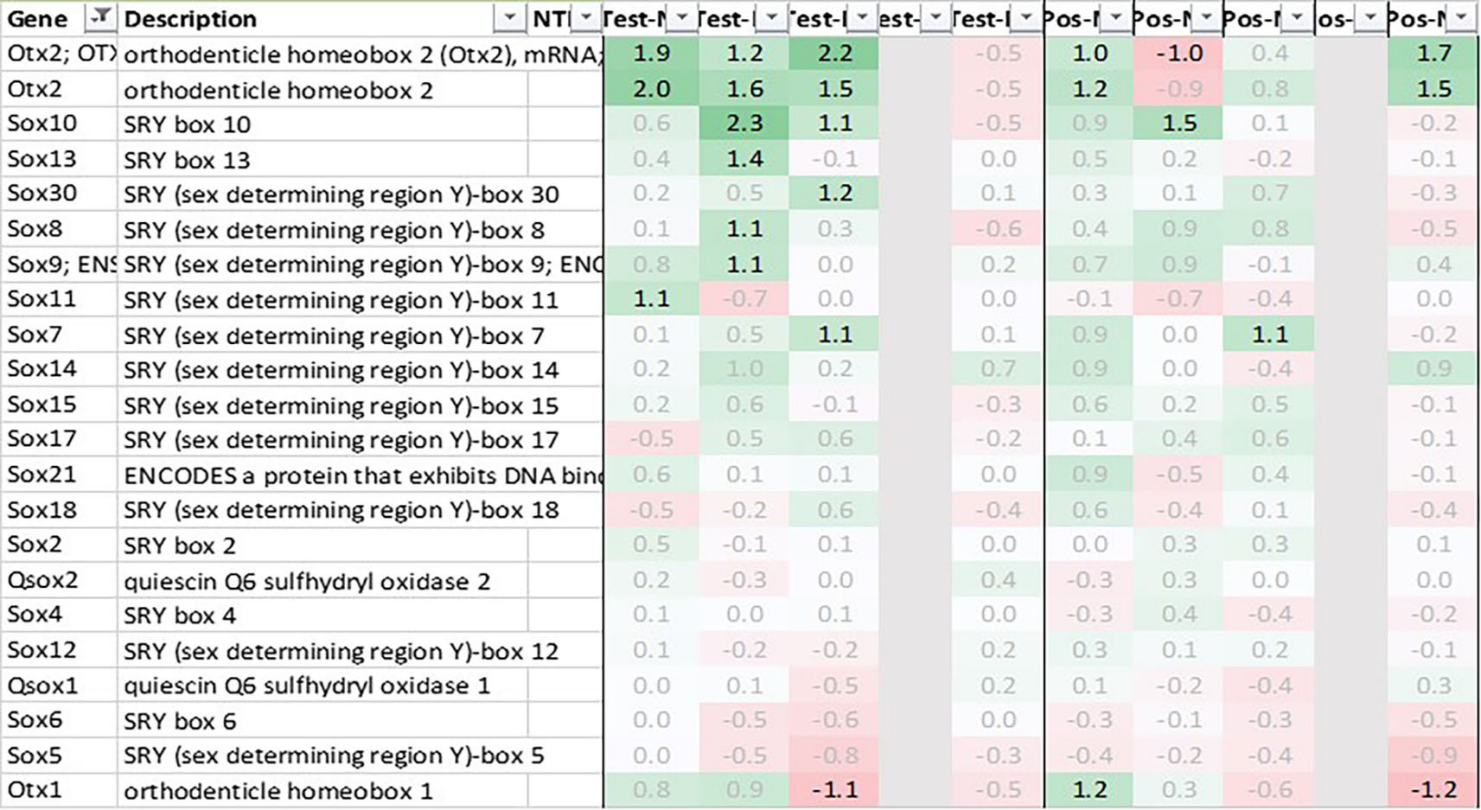
Otx and Sox genes upregulated and downregulated in hypothalamus test vs. negative and positive vs. negative control 0h, +3h, +6h, +24h and +48h post recall.

### Pituitary

In the pituitary samples of test and positive control, we found a heterogenous pattern of expression in steroidogenic, nuclear, and metabolism-related genes. Except Fam111a and Nt5c3b, most of the genes like Cyp11b1/2, Plk5, unknown transcript RGD1564409, and Myo15 showed an upregulation preferentially in test but not in positive; however, as we had to exclude the first two time points in pituitary, most of the upregulation of these genes was observed only +24h post recall. Contrary to this pattern in test, we detected a very strong upregulation of interferon stimulated genes (ISGs) in positive vs. negative control such as Cxcl9/10/11, Gbp5, Rsad2, Ccl2 and 12, Isg15, Oasb1a/b, Ifit2/3, in some cases exceeding regulations by factor 100.

Looking at downregulation, we only observed four genes with an average gene expression downregulated >2fold: ubiquitin-conjugating enzyme Ube2U, Efcab1, Hist2h2ab and Fndc9.

With regard to neurotransmission, we detected an upregulation of ionotropic glutamate receptors like kainate receptor (Grik2) at +6h and monoamine transporter Slc18a1 (VMAT1, endocrine variant of CNS-specific and hypothalamus-upregulated VMAT2), GABA receptors (Gabbr1, Gabre) and opioid binding protein-like (Opcml) upregulation in test pituitary +24h post recall. At the same time, tryptophan hydroxylase Tph1 and GABA-related transporter Slc6a1 were downregulated at +6h post recall, while cholinergic nicotinic neurotransmission-related genes as Chrna9 and Slc44a4, neuron-derived neurotrophic factor Ndnf and proline transporter Slc6a20 were downregulated +24h post recall ( Figure 9).

### Adrenal

In the adrenal glands, 19 genes were found to be upregulated >2fold on average in test animals compared to their negative counterparts. Most of the observed upregulated genes were involved in thermogenesis and metabolic pathways like carbohydrate metabolism (Pck1), lipid metabolism (Cidec, Thrsp), triglyceride catabolism (Fabp3) and heat generation related functions (Ucp1; Thermogenin), which showed a strong and immediate upregulation in test.

In addition to above-mentioned genes, we observed an upregulation of tumor necrosis factor signaling genes (Tnfaip6, Nr4a2), stress responsive gene (Ddit4), gene encoding serine/threonine protein kinase (Sik1), thyroid hormone responsive protein (Thrsp), nuclear receptor (Nr4a2), proto-oncogene (Junb), and cell death-inducing DFFA-like effector c (Cidec).

Looking at downregulation, we observed 9 genes with an average gene expression downregulated >2fold: Phosphodiesterase (Pde01a), Gdpgp1, Mgc11619, LOC31693, Rho family GTPase (Rnd3), Guanosine monophosphate receptor (Gmpr), Myosin protein (Myo16), NONMMU and Golgi vesicular membrane trafficking protein (Bet1l).

Related to neurotransmission in the adrenal glands of test animals, we found an immediate upregulation of adrenergic receptors (Adra1a, Adrb3), neuropeptide Y receptor (Npy4r), purinergic signaling receptor (P2rx5) as well as cholinergic nicotinic receptor (Chrna7). We also observed an upregulation of endorphin precursor proenkephalin (Penk), opioid receptor mu (Oprm1), neuron-derived neurotrophic factor (Ndnf) and dopamine receptor (Drd2) ( Figure 10). We further saw an upregulation in the inhibitory neurotransmission associated genes like GABA receptors (Gabrg1, Gabrg2), excitatory neurotransmitter glutamate/D-serine amino acid transporter (Slc1a4) and muscarinic cholinergic receptor 1 (Chrm1). Moreover, we observed single point downregulation for opioid-related convertase (Pcsk1/2), vesicular glutamate transporter (Slc17a6), GABA transporters (Slc6a1, Slc6a11), and serotonin receptor (Htr3a).

### Spleen

While analyzing the gene expression in the spleen, the expression of 15 transcripts was found to be upregulated on average >2fold across all timepoints measured in test animals as compared to negative control ( Figure 1). These genes included Ig-like domain-containing protein MOR5S4 (“Hiramoto factor”), lymphocyte antigen 6 complex (Ly6al), ubiquitin-like modifier (Isg15), similar to paired-Ig-like receptor A1 LOC690948, signal regulatory protein delta (Sirpd), chemokines Cxcl10 and Ccl12, oligoadenylate synthetase (Oasl), interferon signaling molecules as Ifitm3 and Ifit3, oxidized low density lipoprotein (Olr1), B-cell receptor Cd72, folate receptor 2, RT-1BP, and erythrocyte protein band Ebp4.1. Most of the upregulated genes identified in spleen were either involved in immune activation or in immune function regulation.

Likewise, also many genes downregulated in spleen across all time points measured were related to immune regulatory functions such as G protein signaling regulator (Rgs13) and Killer cell lectin receptor (Klrd1). In addition, we observed a strong downregulation of olfactory receptor Olr1516, detected by two probes, Smarce1l, UDP-Gal:betaGlcNAc beta 1,4-galactosyltransferase polypeptide 4 (B4galt4) and two further uncharacterized transcripts (LOC10254, LOC10091).

Looking at gene expression regarding neuronal and endocrine signaling ( Figure 11), we detected an upregulation of opioid signaling (Pcsk2 and Pcsk1; detected by two independent probes and opioid receptor Sigmar1), nicotinic acetylcholine receptor alpha 3 (Chrna3), ionotropic glutamate receptor delta 2 (Grid2), DNA directed RNA polymerase II (Polr2m), NMDA glutamate receptor ionotropic 1A (Grin1a), D-serine transporter (Slc1a4) and glutamate decarboxylase 2 (Gad2). We also observed a downregulation opioid receptor delta 1 (Oprd1), adrenomedullin 2 (Adm2), cholinergic receptor muscarinic 2 and both glutamatergic (Grik1, Gad2, Grm4), GABAergic (Gabbr1, Gabrp) and serotonergic receptors (Htr1d). Interestingly, exactly opposite as in hypothalamus, we observed an upregulation of neuropeptide Y (Npy) and a downregulation of neuropeptide S (Nps) in spleen.

Taken together, gene expression data obtained from spleen were summarized by a strong upregulation of immunomodulatory genes, chemo-/cytokines and glutamatergic as cholinergic neurotransmission related genes. And similar to hypothalamus, also in spleen we saw a pattern of immediate regulation in positive e.g. of opioid-related transcripts as Pcsk1 or Gpr88, while the same upregulation in test was only observed +3h later.

### Validation of microarray gene expression data by using qRT-PCR

**Figure 5.**
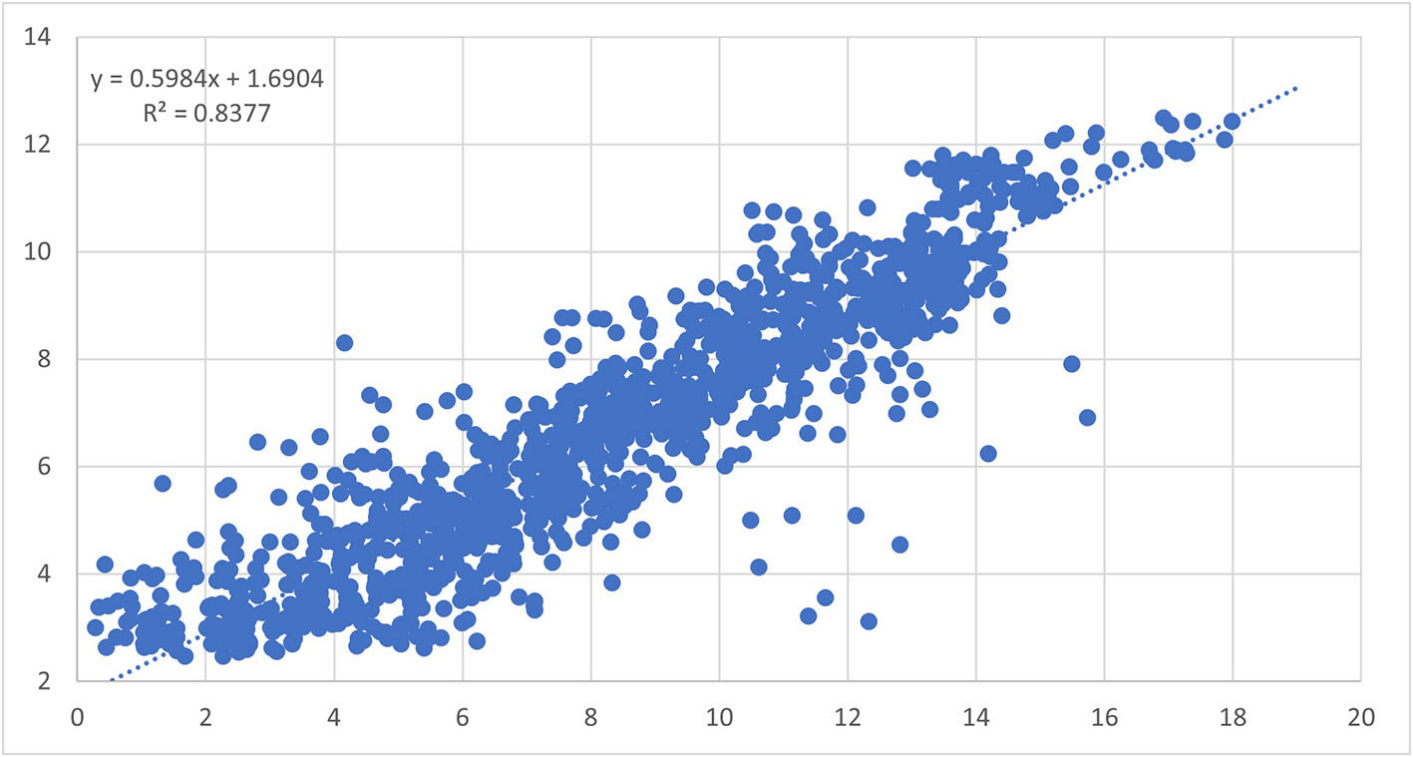
Scatter plot correlation analysis of 32 differentially expressed genes in all four tissues analyzed using Affymetrix chip analysis and qRT-PCR.

To validate the gene expression results obtained by rat full genome microarray analysis, we picked a representative subset of 32 genes including housekeeping genes, interleukins, ISGs, and chemokines, preferably regulated across multiple tissues ( Table 2). ^23^ The mRNA quantity of those genes was analyzed by qRT-PCR and correlation with the corresponding microarray results was assessed. While not all genes correlated perfectly, a strong overall correlation across multiple tissues and expression levels (r ^2^ = 0.8377) was detected ( Figure 5).

**Table 2.** List of 32 genes used for validation of Affymetrix rat genome chip data by qRT-PCR including individual correlation per gene and correlation across all data points.

Gene symbol	Official full name	Correlation
Ccl12	chemokine (C-C motif) ligand 12	0.840
Ccl2	chemokine (C-C motif) ligand 2	0.859
Ccl5	chemokine (C-C motif) ligand 5	0.929
Ccl7	chemokine (C-C motif) ligand 7	0.821
Cd274	CD274 molecule	0.914
Creb3l1	cAMP responsive element binding protein 3-like 1	0.944
Csf1	colony stimulating factor 1 (macrophage)	0.829
Cx3cl1	chemokine (C-X3-C motif) ligand 1	0.877
Cxcl10	chemokine (C-X-C motif) ligand 10	0.890
Cxcl11	chemokine (C-X-C motif) ligand 11	0.872
Cxcl9	chemokine (C-X-C motif) ligand 9	0.851
Fos	FBJ osteosarcoma oncogene	0.898
Fosl2	fos-like antigen 2	0.962
Gbp2	guanylate binding protein 2, interferon-inducible	0.926
Gbp5	guanylate binding protein 5	0.866
Hprt1	hypoxanthine phosphoribosyltransferase 1	0.783
Igtp	interferon gamma induced GTPase	0.939
Il10	interleukin 10	0.907
Il1a	interleukin 1 alpha	0.734
Il1b	interleukin 1 beta	0.913
Il21	interleukin 21	0.347
Irf1	interferon regulatory factor 1	0.865
Irf7	interferon regulatory factor 7	0.954
Junb	jun B proto-oncogene	0.914
Ly49si1	immunoreceptor Ly49si1	0.647
Mmp12	matrix metallopeptidase 12	0.906
Mx2	myxovirus (influenza virus) resistance 2	0.947
Rsad2	radical S-adenosyl methionine domain containing 2	0.928
RT1-A1	RT1 class Ia, locus A1	0.859
Sdha	succinate dehydrogenase complex, subunit A, flavoprotein	0.896
Tnf	tumor necrosis factor	0.759
Usp18	ubiquitin specific peptidase 18	0.932
**Total**		**0.914**

### Plasma cytokine/chemokine levels post recall of the behaviorally conditioned immune response

**Figure 6.**
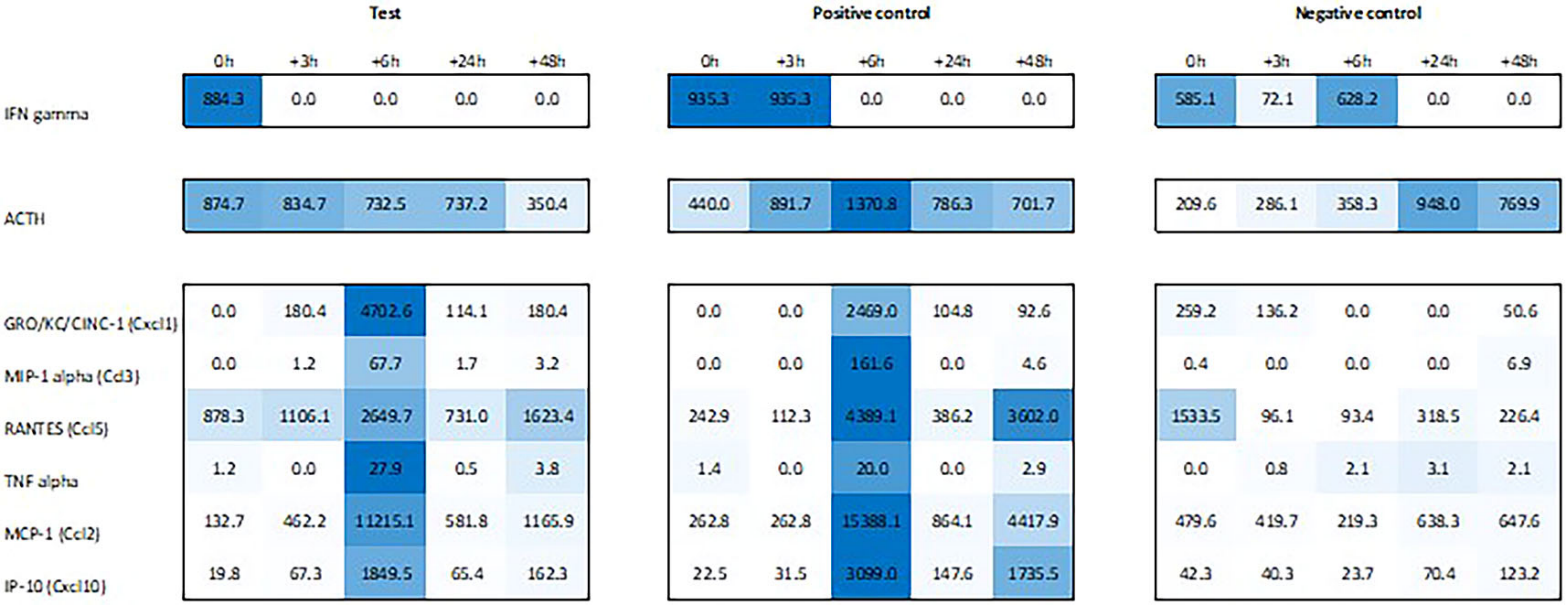
Heat map showing levels of IFN-γ, ACTH, GRO/KC/CINC-1, MIP-1α, RANTES, TNFα, MCP-1 and IP-10 in the plasma of test, positive control, and negative control animals.

Plasma levels of ACTH were found to be increased during the first 6h post recall in both in test and positive control animals, with a peak at +6h in positive. Similar, we observed an increased plasma level of IFN-γ immediately post recall in test and 0h to +3h in positive control. IL-1α showed a similar yet less pronounced response in the plasma of both test and positive control animals, peaking at 0h and +6h post recall while IL-1β showed a similar profile to IFN-γ, peaking 0h to +3h in test and 3h later in positive control. The most pronounced changes were observed for the plasma levels of chemokines GRO/KC/Cinc-1 (Cxcl1), IP-10 (Cxcl10), MCP-1 (Ccl2), MIP-1α (Ccl3), Rantes (Ccl5), and TNF-α, with a strong peak at +6h post recall, both in test and in positive control animals ( Figure 6).

### Sucrose preference test (SPT) and anhedonia

With regard to future studies, we also assessed behavioral data during recall of the conditioned immune response employing the sucrose preference test to evaluate potentially conditioned depression-like anhedonia. During the run-in phase from day -7 to -1, a constant and strong preference for the sucrose solution >90% was observed in all study groups. On D0 (day of 1 ^st^ camphor exposure/poly I:C treatment), a drop in sucrose preference both in test and positive control was observed after poly I:C injection, indicating anhedonia, while there was a less pronounced reduction of sucrose preference in negative control with saline injection, only. Following a return to normal, these effects were further exacerbated on D2 (post recall) with further reduction in sucrose in all animal groups ( Figure 12).

**Figure 7.**
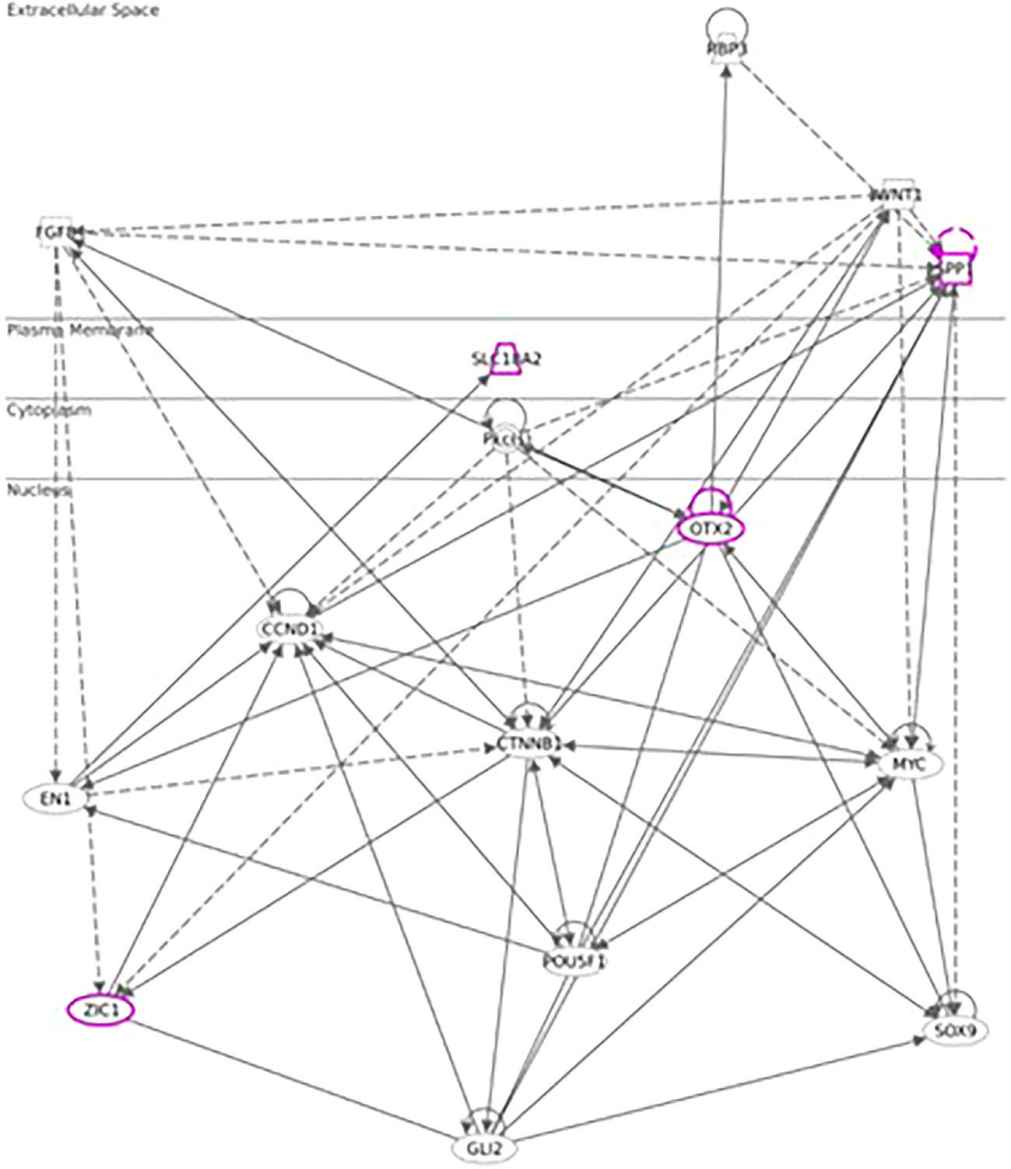
Network chart, depicting potential interaction between upregulated genes connected to Otx-2, Spp1 and Slc18a2 in hypothalamus and potential intermediaries (Qiagen 2020-2022 Ingenuity Pathway Analysis).

**Figure 8.**
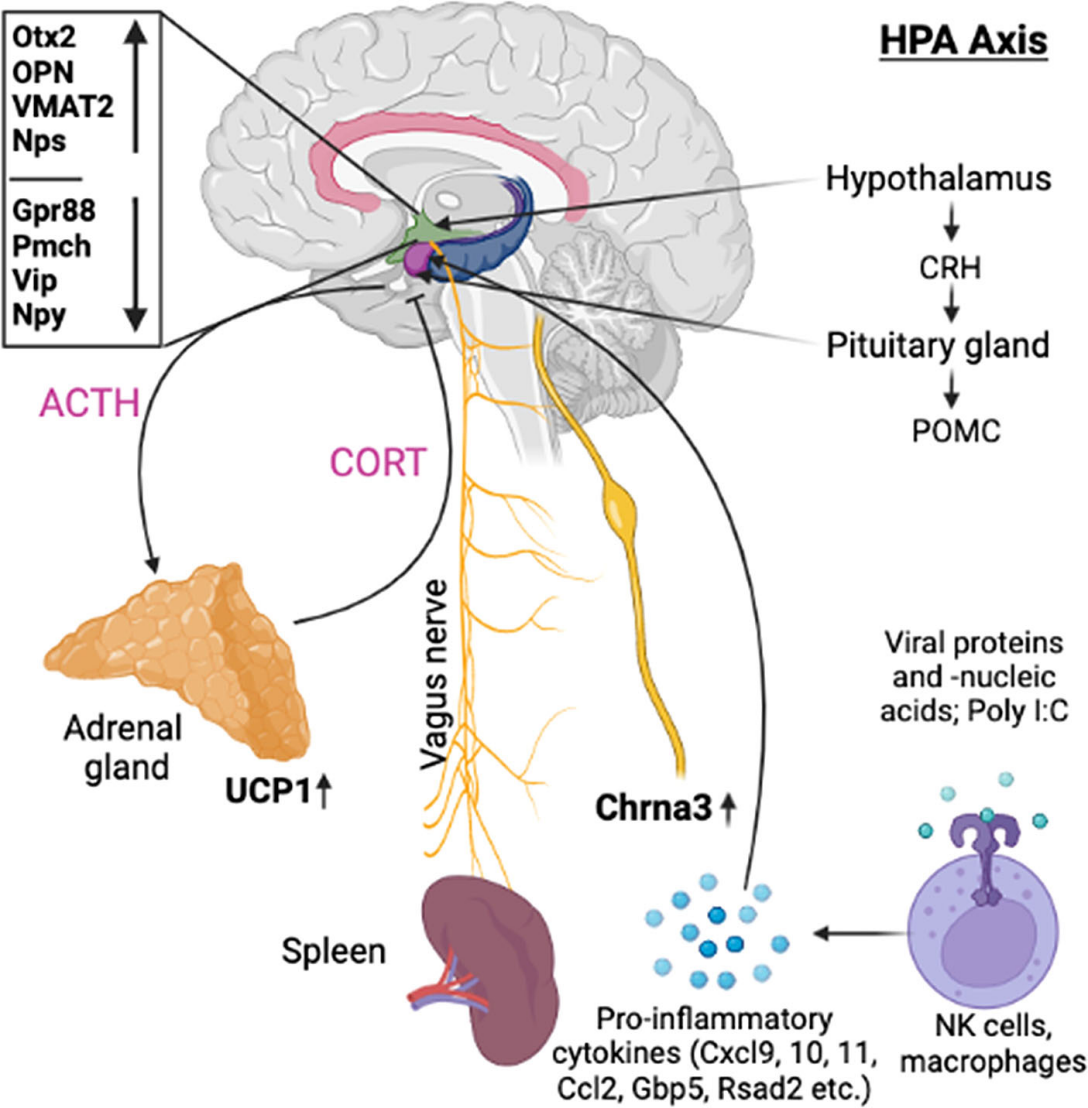
Illustration showing the possible efferent pathway involved in the immune regulation during recall of behaviorally conditioned immune response.

**Figure 9.**
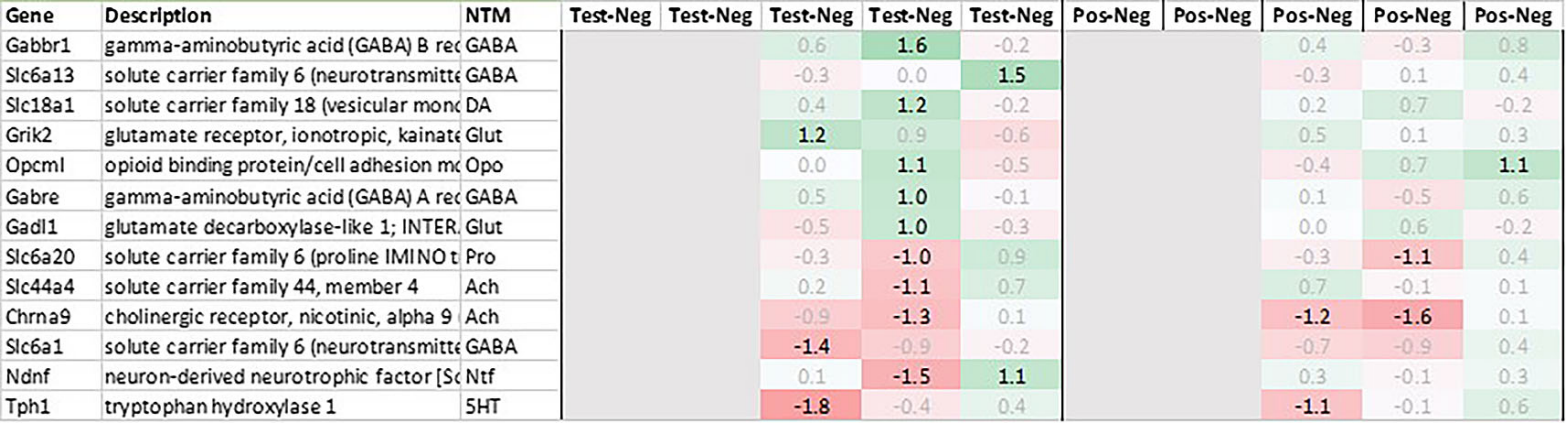
Neurotransmitter-related genes upregulated/downregulated in the pituitary (average regulation test vs. negative and individual time points 0h, +3h, +6h, +24h, and +48h post recall; test vs. negative and positive vs. negative control, respectively).

**Figure 10.**
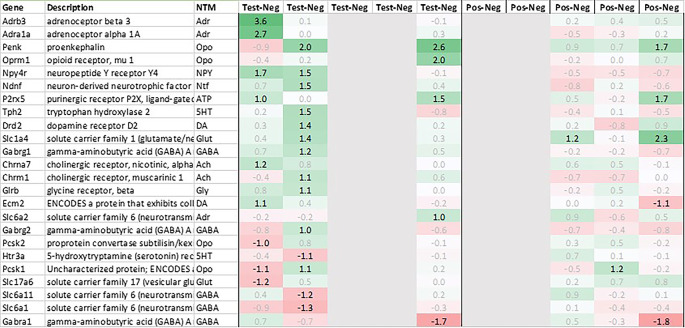
Neurotransmitter-related genes upregulated/downregulated in the adrenal glands (average regulation test vs. negative and individual time points 0h, +3h, +6h, +24h, and +48h post recall; test vs. negative and positive vs. negative control, respectively).

**Figure 11.**
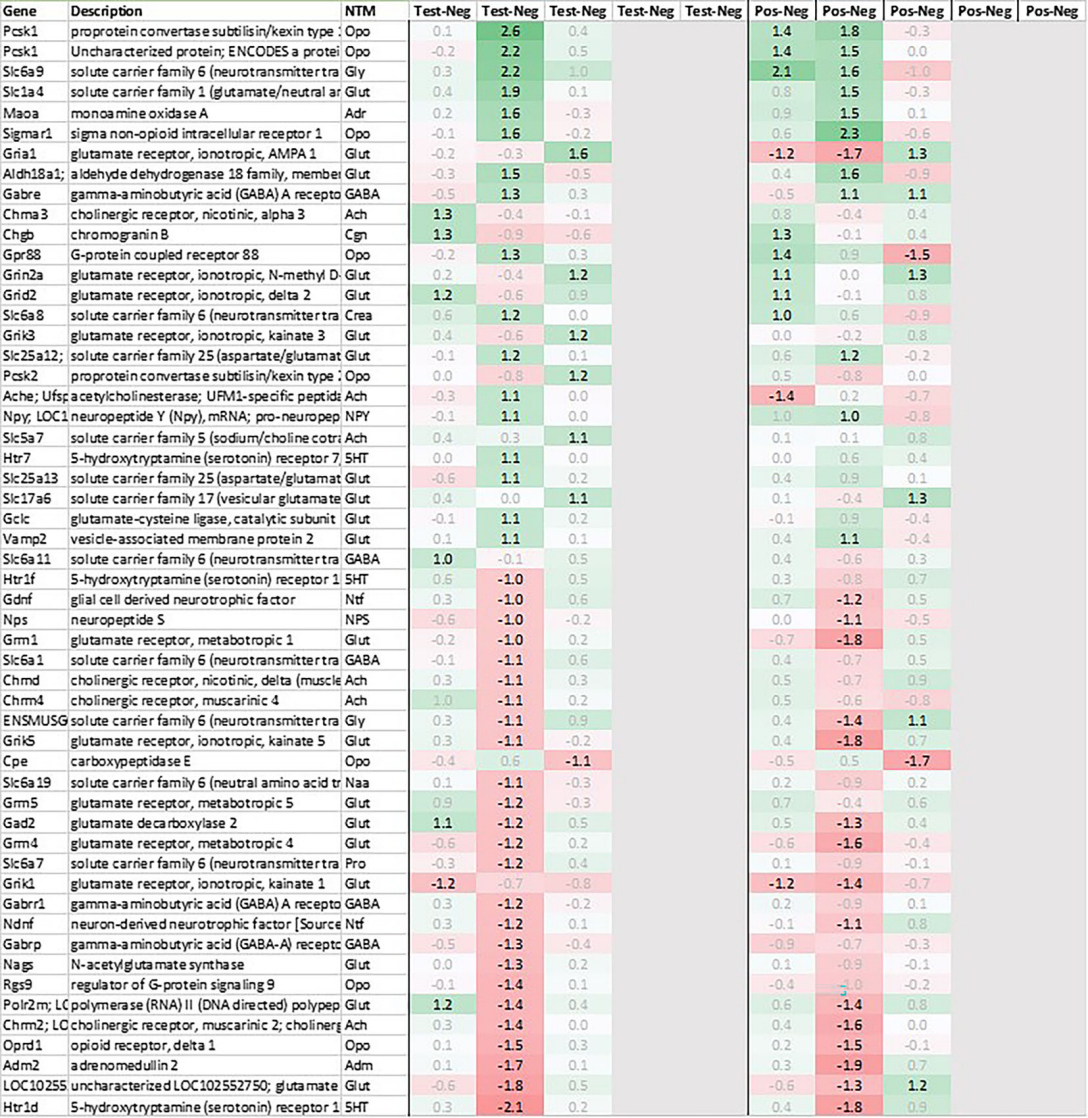
Neurotransmitter-related genes up/downregulated in the spleen (average regulation incl. p test vs. negative and individual time points 0h, +3h, +6h, +24h, and +48h post recall; test vs. negative and positive vs. negative control, respectively).

**Figure 12.**
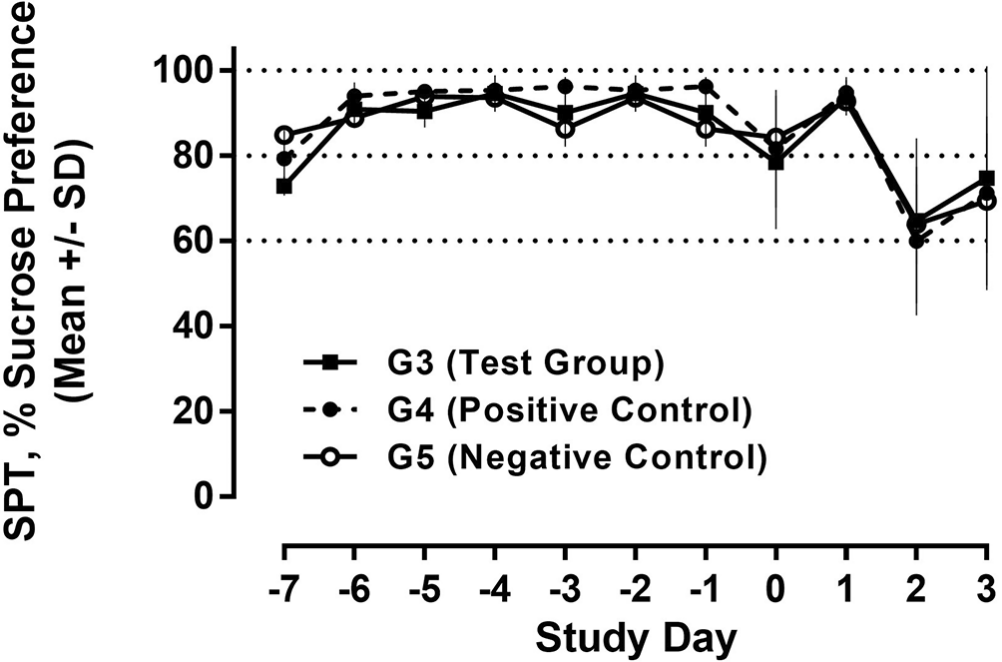
Sucrose preference test (SPT). Water and 2% sucrose solution was presented to all animals, and the relative consumption of sucrose solution is shown as % (100% = exclusively sucrose solution). The consumption over 24h was measured and analyzed for each day.

## Discussion

In the present pilot study, we tried to shed preliminary light on the underlying molecular mechanisms, driving behaviorally conditioned immune enhancement. For that purpose, we employed a conditioning paradigm using camphor smell as conditioned stimulus and an i.p. injection of poly I:C as unconditioned stimulus and hypothesized that the efferent signals for the recall of immune response originate from the hypothalamus. To test our hypothesis, we analyzed differential gene expression in the organs along the HPA axis in test (smell re-exposure and sham injection on D2), positive (poly I:C injection on D0 and D2, only) and negative control (smell re-exposure however sham injection both on day 0 and day +2, respectively) animals along with plasma levels of cytokines/chemokines.

As opposed to our initial hypotheses, it seems there could not only be one but multiple signaling pathways post recall along the HPA axis. Analyzing gene expression patterns in hypothalamus, pituitary, adrenal glands, and spleen, we found evidence for the involvement of WNT/β-catenin pathways, including genes as Otx2, Fzd6, Zic1, and Sox7/8/9/10/11 and 13 in the hypothalamus during the early phases of the recall, corroborating earlier studies and recent studies. ^24^^,^^25^ These findings are complemented by the elevated expression of secreted immunomodulators (osteopontin, OPN) in the hypothalamus and dopaminergic as well as opioid signaling. In the pituitary of test animals, we observed a strong upregulation of steroid synthesis related genes, while gene expression of interferon stimulated genes (ISGs) as Cxcl11, Cxcl10, Cxcl9, Ccl2, Ccl7, Ccl12, Gbp5, Rsad2, Oasb1a/b, and Ch25h seemed mostly confined to positive control. We also identified a strong and immediate upregulation of thermogenesis and prostaglandin synthase associated genes in the adrenal glands and strong upregulation of immunoregulatory molecules in the spleen. Overall, the molecular signaling post recall seems to travel from hypothalamus to pituitary and then onwards to adrenal and spleen, both via neuronal pathways as blood borne messengers and then reach back to the hypothalamus via feedback loops. The same expression pattern can also be observed in positive control; however, here the reaction seems to occur much faster, especially regarding downregulated genes, which show up 3h earlier with poly I:C re-injection compared to re-exposure to the conditioned stimulus.

Looking at the cascade in more detail, one of the key players in the hypothalamus seems to be Otx2, with its immediate and strong upregulation in the test arm, detected by two independent array probes. The upregulation of frizzled receptor Fzd6 and transcription factor Zic1 in test further point towards an involvement of the WNT/β-catenin pathway. While Wnt1 upregulation in the test arm did not reach the >2fold threshold, we observed an immediate upregulation of Wnt5a and Wnt7b in the hypothalamus of test animals. This observation of Wnt regulation in association with genes involved in dopaminergic signal transmission (Slc18a2) links to a recent study by Zhang & Yang, 2021, ^26^ which found two other Wnt genes, Wnt1 and Wnt3a play a critical role in the differentiation of neural stem cells into dopaminergic neurons using tetrahydroxy stilbene glycoside and Wnt-signaling specific inhibitor (IWR1).

Downstream of Otx2, we saw an upregulation in neural transcription factors Sox8, 9, 10, 11 and 13, with Sox11 being upregulated immediately post recall in test and Sox10 showing the strongest upregulation at +3h post recall. Of note, Sox9, 10, and 11 have previously been shown to interact with Otx2 during the neural development ^27^ and regulation of visual cycle gene expression in the retinal pigment epithelium. ^22^ In addition, we also observed a single point upregulation of the homeobox transcription factor Engrailed 1 (En1) +3h post recall in test and immediately in positive. Engrailed 1 has been shown to interact with Otx2, repressing canonical Wnt-signaling during murine embryonic development of the mid and hindbrain and its expression seems to be an important survival factor both for serotonergic and dopaminergic neurons. ^28^

Beside Otx2, two further genes were found strongly upregulated in hypothalamus post recall in test: Spp1, coding for immune regulator osteopontin (OPN) and Slc18a2, coding for vesicle transporter VMAT2. OPN, beside its historical role in biomineralization, has been shown to hold a central role in immune activation and regulation e.g. by interacting with leukocyte integrin receptors (α4β1, α9β1, and α9β4) ^29^^,^^30^ and cell-surface marker (CD44), ^31^ involved in lymphocyte activation, recirculation, and homing. OPN is also a chemoattractant for neutrophils, expressed by wide range of immune cells and has been shown to play a role in immunomodulation by blocking IL-10 in Th2 type cytokine responses and promoting inflammatory IL-12 and Th1 type expansion. ^32^^,^^33^ Considering its pronounced upregulation during the first 6h post recall in test and its humoral immunomodulatory functions, we postulate a central role for OPN in the recall of conditioned immune enhancement, either directly or working in concert with release of CRH and/or other soluble factors into the median eminence, triggering the release of ACTH and further immunomodulatory molecules from the pituitary gland. While this hypothesis is in line with earlier findings by Hsueh et al., ^11^ which demonstrated the involvement of plasma ACTH in the recall of the behaviorally conditioned immune enhancement, except a short peak +3h in positive, we did not detect any upregulation of Pomc, precursor of ACTH and multiple other peptide hormones, on mRNA level in any of the tissues. We did, however, detect an immediate, single point upregulation of IFN-γ gene expression in the hypothalamus of test and a similar upregulation of IFN-α in the spleen +6h post recall of positive control animals.

Another - not necessarily mutually exclusive - option of how OPN might be involved in the signaling cascade during recall of the conditioned immune reaction, could be the HPA feedback loop. As shown by Wang et al., ^34^ OPN -/- mice show reduced levels of ACTH and elevated levels of cortisol during chronic restraint stress and as speculated, same could be due to a negative feedback loop involving OPN. Interestingly, we also detected decreased levels of Cxcl10 and Ccl2, both in hypothalamus of test +6h post recall and immediately post recall in the pituitary of positive control animals. Further to this hypothesis, Trinh et al. in 2020 ^35^ further demonstrated that stress, induced by systemic poly I:C injection, can increase plasma OPN, triggering the release of ACTH. This is in line with the Wang’s observation that OPN injection results in partial restoration of the ACTH response to stress in OPN -/- mice, while an anti-OPN antibody was shown to partially inhibit the stress response in OPN WT mice. ^34^

The third strongly upregulated gene in hypothalamus in test post recall was Slc18a2, encoding vesicular monoamine transporter (VMAT2). VMAT2 is responsible for the transport of cholinergic neurotransmitters norepinephrine and dopamine into synaptic vesicles. Interestingly, both Reserpine, which irreversibly inhibits VMAT2, and 6-Hydroxydopamine (6-OHDA), a neurotoxin inhibiting dopamine signaling, have been shown to downregulate Slc18a2 transcription, blocking both the conditioned recall of an enhanced NK reaction ^36^ and the conditioned recall of the enhanced neutrophil reaction. ^12^ As further shown by Hsueh et al., ^11^^,^^12^^,^^37^ both cholinergic and serotonergic pathways in the CNS seem to be involved during acquisition as well as recall of the conditioned NK cell response and same has been shown to involve both muscarinic and nicotinic receptors. In line with these data, we saw a downregulation of both dopamine receptors (Drd1, Drd2) and serotonergic receptors (Htr1f, Htr2a) in the hypothalamus of test animals +3h post recall, further pointing towards an involvement of dopaminergic and/or noradrenergic signaling pathways during the recall of the conditioned response. Figure 7 shows the putative interaction between the upregulated genes and potential interactors.

Additional genes strongly downregulated in hypothalamus included pro-melanin-concentrating hormone gene (Pmch), precursor of the orexigenic peptide MCH, vasoactive intestinal peptide (Vip) as well as neuropeptide Y (Npy), together with an upregulation of neuropeptide S (Nps). MCH, in addition to its role in appetite stimulation, has been shown to play a critical role in regulating dopamine signaling by suppressing DA release in the nucleus accumbens while NPY seems to play a similar role by directly inhibiting dopaminergic neurons in the ventral tegmental area (VTA). ^38^ Neuropeptide S has the exact opposite effect as neuropeptide Y, acting anxiolytic and anorectic, which adds to the pattern of appetite-reduction and energy conversation by immediate and strong downregulation of Pmch and Vip, further augmented by downregulation of Npy and upregulation of Nps, observed immediately in positive and +3h post recall in test.

We also observed a strong downregulation of opioid inhibitory G-protein coupled receptor 88 (Gpr88) and regulator of G-protein signaling 9 (Rsg9) in concert with an upregulation of prepronociceptin (Pnoc) and proprotein convertase subtilisin/kexin type 1 inhibitor (Pck1n), detected by two independent probes; like with Npy and Nps, regulation was observed +3h in test and immediate in positive. Again, these observations are in line with former studies by Hsueh et al., ^39^ which have demonstrated the involvement of opioid pathways during the recall of behavioral conditioned immune enhancement.

Finally, we also identified RT-1A upregulated in both hypothalamus and adrenal of test animals. RT-1A is a classical rat MHC class Ia gene, involved in the presentation of peptides to cytotoxic T lymphocytes and has been shown to interact with the Ly-49 family of receptor on NK cells as “self-identifier”, thereby providing protection against NK cell lysis. ^40^

Most of the other genes we observed regulated in hypothalamus shortly post recall such as cilia and flagella associated protein Cfap43, involved in olfactory detection, Npas4, key regulator of GABAergic synapse development, stabilizing the activity of neurons during glutamatergic input, or dynein subunits Dnah12 and Dnah1, point towards a heavy involvement of microtubular transport processes during the recall of the conditioned immune reaction.

Pituitary onwards, we observed an upregulation of steroidogenic, nuclear, and metabolism-related genes like Fam111, Star or Rgs1 in test animals post recall as opposed to a strong upregulation of ISGs in positive. Same view, however, might be distorted as we were not able to analyze time points +0h and +3h in pituitary, and hence might have missed upregulation during first 3h.

Independently of this, we did observe an upregulation of orphan nuclear receptors Nr4a2 and Nr4a3 in test - while the exact function of both receptors has not been fully elucidated, recent studies point towards a role in antigen presentation and viral response. ^41^ Downregulation did not yield a consistent pattern and beside few, single point downregulations as observed with Tetraspanin (Tspan8) and Claudin (Cldn1), only serotonin-related tryptophan hydroxylase Tph1 showed a clear pattern. This rather mixed expression pattern in test is in strong contrast to the clear and strongly ISG-dominated expression pattern in positive control, showing more than 50x upregulation for genes like Cxcl9, 10, 11 or Gbp5, Rsad2, and Ccl2, which points to a different mechanism when comparing poly I:C re-injection to the recall of the conditioned immune reaction.

Further along the HPA axis, the strong immediate upregulation of Ucp1 (>300fold) in the adrenal glands of test animals point towards a dramatic shift in metabolism towards heat generation as e.g. required for fever induction. Likewise, strongly upregulated Thrsp has also been shown to play a role in thermogenesis of brown adipocytes, ^42^ which was recently confirmed in fed and refed hatchling chicks. This metabolic shift towards heat generation is also in line with the upregulation of fatty acid binding protein Fabp3, involved in the regulation of mitochondrial thermogenesis and temperature homeostasis. At the same time, we saw the upregulation of both adrenergic receptors Adr1a and Adr3b. Both receptors have been shown to be involved in metabolic abnormalities and associated diseases and mutations in these receptors have been speculated to be associated with weight gain in association with anti-psychotic drugs, further corroborating a role of gene expression towards metabolic adaptation to sickness-related conditions.

In the spleen, we saw a strong upregulation of known and partially unknown immunomodulatory genes. The highest upregulation was observed for a 354bp uncharacterized transcript, coding for a 118aa Ig-like domain containing protein (M0R5S4; “Hiramoto factor”). Reactome analysis suggests the involvement of this protein in immunoregulatory interaction between lymphoid and non-lymphoid cells involving lymphoid expressed Fc-gamma receptors and/or in signaling of the B cell receptor. The second upregulated gene, Ly6aI, also known as stem cell antigen 1 (SCA-1), has recently attracted attention after it was identified as key gene in the transport of neurotropic vector adeno-associated virus serotype 9 across the blood-brain barrier in C57BL/6J mice. ^43^ Its classic function, however, is more known in hematopoiesis; in fact, Sca1 is one of the most common cell surface marker used to enrich adult hematopoietic stem cells. Furthermore, a regulatory role of Ly6 proteins in nicotinic acetylcholine receptors (nAChRs) has been suggested. ^44^

In addition to M0R5S4 and Ly6aI, many other genes upregulated in spleen fall into the category of immunomodulation and -regulation. RT1-Bb is a member of the MHC class II family interacting with CD4 receptors on helper T cells. SIRPδ (Sirpd) is a member of the signal regulatory protein family, involved in signal transduction and cell adhesion and more researched member SIRPα acts as inhibitory receptor by interacting with transmembrane protein CD47, controlling effector functions of the innate immune response. ^45^ Loc690948, predicted to code for Lilrb3a, is an inhibitory member of the leukocyte immunoglobulin-like receptor family and LILRBs have been documented on a broad range of immune cells including NK cells, where they can modulate immune cell functions as cytokine release, antibody production and -presentation ^46^ as well as Toll-like receptor signaling. ^47^ Apol11a, coding for a Apolipoprotein L, a Bcl-2 like protein, has been proposed to have cell death related function due to their pore-forming domain, inflicted by dendritic cells after viral stimulation and also gets strongly expressed splenic DCs post stimulation with poly I:C, dependent on TLR3/TRIF, a reaction mimicked by IFN-ß. ^48^ CD72 is an inhibitory co-receptor on B cells, that recognizes the RNA-containing Sm/RNP (Smith/Ribonucleoproteins as resulting from cell death) by an extracellular C-type lectin domain (CTLD) while inhibiting the corresponding B cell response through it intracellular immunoreceptor tyrosine-based inhibition motif (ITIM), thereby blocking the TLR7-mediated activation of antibody production. ^49^ Finally, RT1-N2 belongs to the family of rat MHC class Ib molecules. This class has recently been shown to interact with both inhibitory and activating Ly49 receptors, resulting in the specific expansion of allospecific NK cells. ^50^ All these findings point towards a central role for spleen in the modulation of the behavioral conditioned immune enhancement.

With reference to Hsueh et al. ^11^ original study in mice, we also looked at IFN-related gene expression in spleen and while we couldn’t find an upregulation of Ifn-α or -β in spleen of test, we did observe an upregulation of Ifn-α2 gene expression in positive +6h post recall. More importantly, we also saw an immediate peak of Ifn-γ expression in hypothalamus of test, also reflected by elevated IFN-gamma levels in plasma. Likewise in line with Hsueh et al., we did observe elevated ACTH plasma levels in test and positive during the first 6h post recall but not in negative control animals ( Figure 6).

For other chemokines, we saw a rather mixed pattern when comparing plasma levels and gene expression: while we did see a peak of GRO/KC/Cinc-1, MIP-1α, and Rantes +6h in plasma of both test and positive control, a corresponding upregulation of Cxcl1, Ccl3 and Ccl5 on gene level could only be observed in pituitary and adrenal of positive. Likewise, a plasma peak of TNF-α did show a corresponding upregulation of Tnf gene expression. For plasma MCP-1, we observed immediate upregulation of Ccl2 in the spleen of positive and 3h later in spleen of test. This pattern resembles very much the expression of Cxcl10 and corresponding plasma levels of IP-10, where we first saw an upregulation of Cxcl10 in spleen of positive and 3h later in test plus a similar, 3h delayed pattern in hypothalamus. This pattern, an upregulation in spleen, first, and 3h later in hypothalamus, starting in positive, immediately and 3h delayed in test, was also observed for other ISGs as Cxcl11, Gbp5, and Rsad2, and might suggest that the upregulation of ISGs in hypothalamus is rather a secondary effect, triggered by an upregulation of ISGs in spleen.

In this context, it might be interesting to mention that we also observed a short but clear upregulation of acetylcholine receptor Chrna3 in the spleen of test animals immediately post recall. Most of the available literature suggest Chrna7 as the main receptor for acetylcholine ^51^ in the spleen, involved in the relay of vagus nerve signals to cytokine-producing macrophages via acetylcholine-synthesizing T cells in the spleen ^52^ and as recently discovered, this Chrna7-based activation of macrophages seems to be mediated by a downregulation of α1,3-Fucosyltransferase Fut7. ^53^ We could not see any upregulation of Chrna7 in spleen, however we did see a downregulation in Fut7 +3h post recall in both test and positive, hinting towards a potential involvement of the vagal system in modulating the recall of the conditioned immune reaction. And while a recent paper by Verlinden et al. 2019 ^54^ demonstrated catecholaminergic but no direct cholinergic innervation of the spleen, this finding does not rule out the possibility of an indirect vagal innervation via postganglionic non-cholinergic fibers, further supported by a recent study by Zhang et al. ^55^ that has shown that CRH neurons originating from the CeA of the amygdala and PVN of the hypothalamus can stimulate splenic plasma cells formation via direct splenic innervation, potentially via intermediate T cells, translating the noradrenalinergic signal into an acetylcholinergic message.

In summary, our data indicate that in addition to the classic HPA axis involving CRH, ACTH and cortisol release, there could be additional pathways connecting brain and immune system during the recall of behavioral conditioned immune enhancement e.g. by direct splenic innervation, modulating and finetuning the message via cholinergic signaling from the PVN. We further postulate that recall of the conditioned immune response starts in the hypothalamus via a Wnt/β-catenin related pathway, involving Otx2, Sox7/8/9/10/11, Zic1, and Fzd6 as well as monoaminergic (Slc18a2) and opioid messaging (Gpr88). While still to be analyzed in more detail, both osteopontin as strongly upregulated, potentially in concert with Pmch and Vip as strongly downregulated immediately post recall, seem to play an important role during the early phases - either in the communication between hypothalamus and pituitary and/or as blood borne immune modulators themselves. While pituitary onwards, it is hard to identify one specific pathway, we observe the upregulation of steroidogenic and thermogenic genes in pituitary and adrenal, respectively, pointing towards an early hormonal activation in parallel to a strong immunomodulation observed in spleen, from where the feedback loop involving strongly upregulated ISGs as Cxcl9/10/11 reaches back to the brain.

## Data availability

Underlying data deposited in Zenodo.org: https://doi.org/10.5281/zenodo.7086375. ^56^

• 20220902 Affymetrix all tissues all timepoints red green incl p v2.xlsb (Affymetrix raw data and analysis across all tissues and time points)
• 20220803 Ct values and Affymetrix correlation v2.xlsx
  (qRT-PCR Ct values and corresponding Affymetrix data for 32 genes)

### Reporting guidelines

Zenodo: ARRIVE checklist for *“Differential gene expression during recall of behaviorally conditioned immune enhancement in rats: a pilot study”*, https://doi.org/10.5281/zenodo.7086375. ^56^

Data are available under the terms of the Creative Commons Attribution 4.0 International “No rights reserved” data waiver ( https://creativecommons.org/share-your-work/public-domain/cc0/)
