# Differential gene expression during recall of behaviorally conditioned immune enhancement in rats: a pilot study

**DOI:** 10.12688/f1000research.123975.3

**Published:** 2025-01-10

**Authors:** Markus Rueckels, Marcus Picard-Mareau

**Affiliations:** 1 Lisa-Kolk-Stiftung, Berg. Neukirchen, North Rhine Westphalia, 51381, Germany

**Keywords:** Behavioral conditioning, NK cells, gene expression analysis, HPA axis, poly I:C, campher smell, Otx2, Sox11, Wnt/β-catenin pathway, Slc18a2, VMAT2, Spp1, OPN, Osteopontin, Gpr88, Fzd6, Zic1, Pmch, Npy, Nps, Chrna3, CAIP, ACTH, IFN-α

## Abstract

**Background:**

Behaviorally conditioned immune functions are suggested to be regulated by bidirectional interactions between CNS and peripheral immune system *via* the hypothalamic-pituitary-adrenal (HPA) axis, sympathetic nervous system (SNS), and the parasympathetic nervous system (PNS). Since the current knowledge about biochemical pathways triggering conditioned immune enhancement is limited, the aim of this pilot study was gaining more insights into that.

**Methods:**

Rats were conditioned with camphor smell and poly I:C injection, mimicking a viral infection. Following stimulus re-exposure, animals were sacrificed at different time points, and neural tissues along the HPA axis was analyzed with a rat genome array together with plasma protein using Luminex analysis.

**Results:**

In the hypothalamus, we observed a strong upregulation of genes related to Wnt/β-catenin signaling (Otx2, Spp1, Fzd6, Zic1), monoaminergic transporter Slc18a2 and opioid-inhibitory G-protein Gpr88 as well as downregulation of dopaminergic receptors, vasoactive intestinal peptide Vip, and pro-melanin-concentrating hormone Pmch. In the pituitary, we recognized mostly upregulation of steroid synthesis in combination with GABAergic, cholinergic and opioid related neurotransmission, in adrenal glands, altered genes showed a pattern of activated metabolism plus upregulation of adrenoceptors Adrb3 and Adra1a. Data obtained from spleen showed a strong upregulation of immunomodulatory genes, chemo-/cytokines and glutamatergic/cholinergic neurotransmission related genes, as also confirmed by increased chemokine and ACTH levels in plasma.

**Conclusions:**

Our data indicate that in addition to the classic HPA axis, there could be additional pathways as e.g. the cholinergic anti-inflammatory pathway (CAIP), connecting brain and immune system, modulating and finetuning communication between brain and immune system.

## Introduction

Pavlovian, sometimes also called classical conditioning, is a phenomenon of associative learning, in which a behavioural response is induced by establishing a temporal pairing between a conditioned stimulus (CS) and an unconditioned stimulus (US). ^1^ In addition to the well-known phenomenon of classic conditioning of physiological reflexes, immune responses can also be memorized and recalled using Pavlovian conditioning, based on a reciprocal communication between the central nervous system (CNS) and the peripheral immune system. ^2^

This concept of behaviourally conditioned immune modulation was rediscovered by Ader and Cohen ^3^ in the 1970s, who used cyclophosphamide as immunosuppressive agent (US) and illness-induced taste aversion by lithium chloride (LiCl) solution as CS. They then measured the changes in immune responsiveness by a specific antibody reaction, using sheep erythrocytes as antigens. Since the study was published, this phenomenon of conditioned immunosuppression has been replicated in a multitude of studies ^4^^–^^7^ by different researchers all over the world, and current knowledge about this phenomenon, including potential pathways at work and experimental paradigms employed, has been comprehensively summarized in a recent review by Hadamitzky *et al.* ^8^

While most of our knowledge of the conditioning of immunological functions is derived from immunosuppression paradigms, relatively few studies have focused on immune enhancement. A series of these studies targeted the conditioned enhancement of both, cytotoxic T-cells (CTL) and natural killer (NK) cells in rodents, identifying multiple possible signaling molecules and pathways driving these effects. ^9^^–^^12^

Historically, behaviourally conditioned immune functions have been suggested as being regulated by bidirectional interaction between CNS and peripheral immune system via the hypothalamic-pituitary-adrenal (HPA) axis, sympathetic nervous system (SNS), and the parasympathetic nervous system (PNS). ^13^ In addition to the HPA axis, the brain limbic system (cortex, hippocampus, amygdala) has also been associated with behaviourally conditioned immune responses. ^13^ In a previous study, ^11^ both adrenocorticotropic hormone (ACTH) and interferon-alpha (IFN-α) were shown to be involved in the efferent signaling pathways during recall of the conditioned enhancement of NK cell activity. ^11^ However, the exact biological basis (biochemical, neuroanatomical, genetic) and the underlying molecular mechanisms driving these processes are still unclear.

To shed more light on these pathways, we conducted a pilot study, using the formerly published 3-day conditioning paradigm originally developed by Hsueh *et al.* ^11^ Following that paradigm, all animals were first exposed to camphor smell and animals in the test and positive control group then injected with polyinosinic:polycytidylic acid (poly I:C), simulating a viral infection and inducing so-called sickness behaviour, an animal model for depression-like behaviour in rodents; animals in the negative control group were injected with saline. ^14^^,^^15^ After a 48h interval, animals in the test group were re-exposed to camphor smell to recall the behaviourally conditioned immune response in rats, while animals in the positive control group received a re-injection of poly I:C only. Animals in the negative control group received smell re-exposure and an injection of saline.

We then used a gene-agnostic expression analysis approach to identify first gene candidates along the four major tissues – hypothalamus, pituitary, adrenal glands, and spleen – of the HPA axis, while in parallel measuring cytokine and chemokine protein levels in the plasma of the conditioned animals. Results from both approaches were combined and aligned with the existing literature to provide a list of candidate genes and proteins for future studies.

## Methods

### Animals

Male, Sprague Dawley (SD) rats (5-7 weeks, 160-250 g) were obtained from InVivos Pte Ltd, Singapore. Male animals were chosen to minimize the impact of hormone-related gene expression and behavioural readouts. Animals were housed at the Biological Resource Centre (BRC) in groups of two in individually ventilated cages and maintained on a 12h light/dark cycle with food and water *ad libitum* in accordance with the guidelines of the Agency for Science, Technology and Research (A*STAR) Animal Care and Use Committee. All efforts were made to ameliorate any suffering of animals; animals were provided with Nylabones (hard, non-toxic nylon), nestlets, and/or domes for environmental enrichment to ensure adequate welfare and psychological well-being. In accordance with Institutional Animal Care and Use Committee (IACUC) guidelines, animals were allowed to acclimatize for a minimum of three days and examined to confirm their health status prior to study commencement.

### General conditioning procedure and tissue collection

Animals (N=30) were randomly allocated into two series (a and b), each containing 15 animals, and further divided into three experimental groups as shown in Table 1. A 3-day conditioning paradigm was used in the study as described previously ^11^ where camphor smell was used as the conditioned stimulus (CS) and poly I:C injection (i.p.) as the unconditioned stimulus (US). In the present study, camphor smell exposure was performed in a separate procedure room for 1h using 10 cm petri dishes filled with 5 g of camphor powder (Sigma #21310) placed above the cage (on top of the metal grid) and warmed up with a heat lamp. On day D0, animals from all three groups were exposed to camphor smell for 1h following exposure to saline (i.p.; 10 ml/kg; negative control group) or poly I:C (Sigma #P1530; i.p.; 1 mg/kg; test and positive control group). ^16^ On day D2, animals from the test and negative control groups received saline injections (i.p.), following exposure to camphor smell for 1h, whereas animals from the positive control group were re-injected with poly I:C, only. After the last injection on day D2, animals from each group were sacrificed at 0h (n=2), +3h (n=2), +6h (n=2), +24h (n=2) and +48h (n=2) postinjection, and tissues were collected (hypothalamus, pituitary, adrenal glands, and spleen) and stored snap frozen in RNAlater (MilliporeSigma, Burlington, U.S.A.) for further analysis. Blood was collected for the isolation of peripheral blood mononuclear cells (PBMCs) and plasma was collected to perform cytokine and chemokine Luminex analysis. Animal experiments were conducted at the Biological Resource Centre (BRC), Biomedical Sciences Institutes, Agency for Science, Technology and Research (A*STAR), 20 Biopolis Way, #07-01 Centros Building, Singapore 138668.

**Table 1.** Animal groups and conditioning protocol including timeline.

Group number	Group name	1 ^st^ treatment (Day D0)	2 ^nd^ treatment (Day D2)
1	Test (n=5)	Camphor smell + Poly I:C	Camphor smell + Saline
2	Positive control (n=5)	Camphor smell + Poly I:C	Poly I:C
3	Negative control (n=5)	Camphor smell + Saline	Camphor smell + Saline
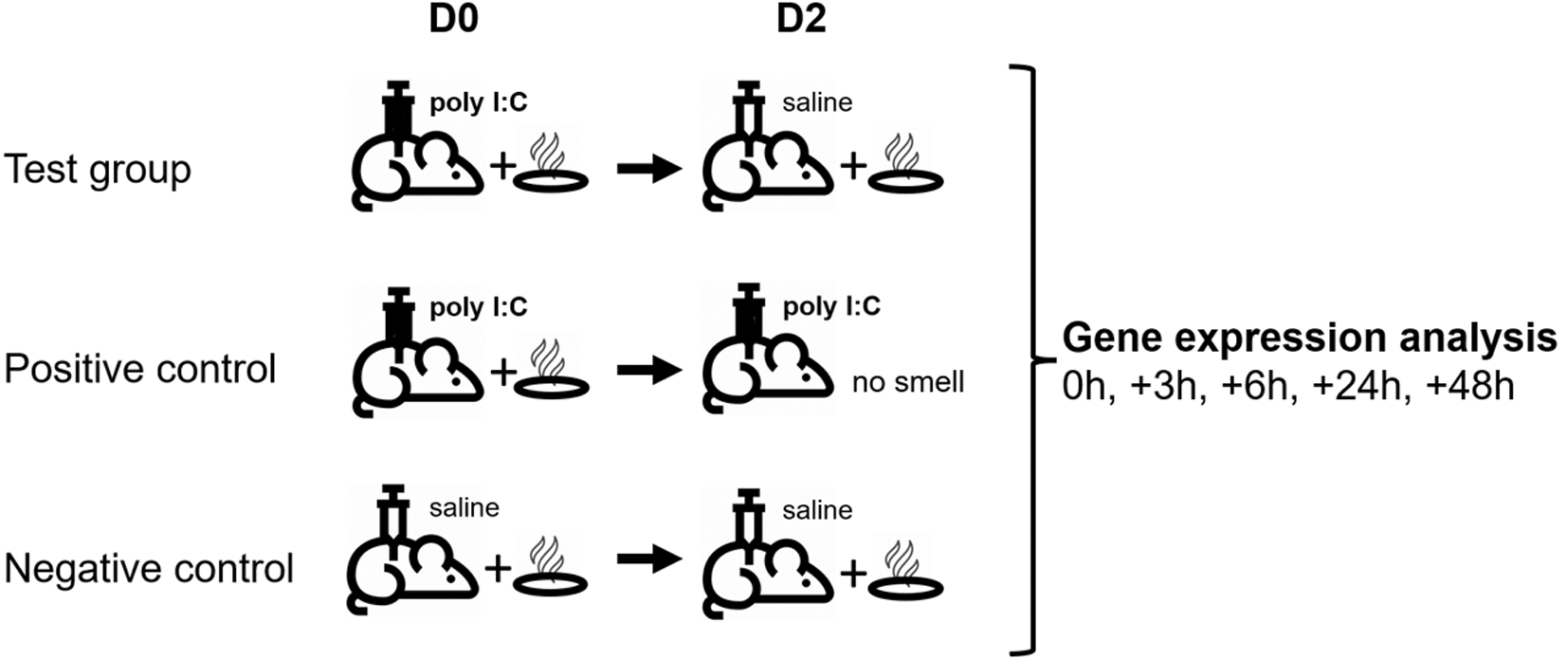			

### Sucrose preference test (SPT)

The SPT is a standard behavioural test to assess depression-like behaviour in rodents during the development of anti-depressive treatments. The SPT is based on anhedonia (lack of interest in rewarding stimuli), which is present in some forms of affective disorders including depression. In this test, the interest of animals in seeking out a sweet rewarding drink relative to plain drinking water is used and a bias toward the sweetened drink is typical, failure to do so is an indication of anhedonia/depression. ^17^ The SPT was carried out on D-7 to D-1 (run-in phase for baseline data) as well as days D0 - D3. Rats were presented with two drinking bottles, one with plain drinking water and one with 2% (w/v) sucrose solution for 24 h. Except for days D2 and D3, all 30 animals were analyzed; due to sacrifize after treatment on D2, only 12 animals were analyzed on D2 and 6 animals on D3. The ratio of sweetened vs. plain water consumed during this period was calculated, and results were expressed as %sucrose preference.

### Affymetrix microarray analysis

At the end of the study, mRNA from all tissues harvested at different time points was isolated by using an RNeasy Plus mini kit (QIAGEN, Hilden, Germany). RNA quality was assessed by using a Bioanalyzer 2100 (Agilent Technologies, Santa Clara, U.S.A.). Highly purified RNA was subjected to microarray analysis with a GeneChip ^TM^ Rat Gene 2.0 ST Array (Affymetrix, Inc, Santa Clara, U.S.A.) to identify differential gene expression during the recall of behaviourally conditioned immune responses. RNA labelling, hybridization, staining, and scanning were performed according to the manufacturer’s instructions. Briefly, 100 ng of total RNA from each sample was reverse transcribed to cDNA, followed by overnight *in vitro* transcription to generate cRNA which was further fragmented and labelled. The quality of cDNA and fragmented cRNA was assessed with an Agilent bioanalyzer. We used the Robust Multichip Average (RMA) model for array background correction and quantile normalization as described previously. ^18^^–^^20^ Probes were mapped to the Rattus norvegicus genome chip (RGSC 5.0/rn5) and results from all tissues were normalized using β-actin.

### Analysis of differentially expressed genes

Gene expression results for all test and positive control samples were matched by tissue and time point with their respective counterparts in the negative control group and subtracted to derive single point gene expression differences (all data expressed as 2^x). Due to insufficient RNA quality after extended storage, we could not analyse all tissue samples by microarray and had to discard multiple samples (hypothalamus negative control +24h, pituitary test and positive control 0h and +3h, adrenal test +6h and +24h and positive control 0h and +3h, spleen +24h and +48h all samples); accordingly, we first limited the analysis to the average across all available time points and then examined single-point regulations with a focus on genes related to neurotransmitters in greater detail; in each type of tissue, a minimum of three individual time points were available. Following gene enrichment and comparison of test vs. negative and positive vs. negative control selection, all differentially expressed genes per tissue were then analysed by Reactome pathway analysis. ^21^ As we hypothesized the origin of the message post recall was from the hypothalamus, we additionally leveraged QIAGEN Ingenuity Pathway Analysis (QIAGEN IPA) for a more detailed analysis of hypothalamic genes. All genes with an average regulation of >2fold in the hypothalamus of test animals were selected, and a subcellular network analysis was conducted using an IPA graphic interface.

### Quantitative RT-PCR

The same RNA of all samples as used in the microarray analysis was also subjected to quantitative reverse transcription PCR (qRT-PCR) to confirm the observed differential gene expression. The qPCR laboratory work was conducted as a fee-for-service by the Qiagen service laboratory (Qiagen Life Science Service & Support, Hilden, Germany), using the RT ^2^ Profiler ^TM^ PCR custom Array RAT (CLAR27943). Briefly, RNA was isolated, and cDNA was synthesized with Qiagen RT ^2^ First Strand Kit using 500 ng of RNA, 1 μl RT primer mix (oligo-dT and random hexamer primers), 4 μl Quantscript RT buffer (5X) and 1 μl Quantiscript Reverse Transcriptase (QuantiTect kit, Cat. No. /ID: 205311, Qiagen). qPCR reactions were then assembled using synthesized cDNA (1 μl), 5 μl RT ^2^ Profiler PCR Array SYBR Green master mix (Qiagen, Germany), 1 μl of each primer diluted to a 5 μM working solution and 1 μl sterile water and processed in a 96-well format including housekeeping genes, RNA and DNA controls, using Qiagen’s proprietary primer panel. The mRNA expression was determined using the 2− ^ΔΔCT^ method. ^22^ All gene expression values were normalized using Hypoxanthine phosphoribosyl transferase (HPRT), glyceraldehyde phosphate dehydrogenase (GAPDH), and β-actin as references for expression analysis of genes of interest.

### Cytokine array analysis

Plasma protein levels for all animals and time points were evaluated as a fee-for-service by Singapore Immunology Network (SIgN), coordinated by Biomedical Research Council (#04-06 Immunos, Singapore), using the MILLIPLEX ^®^ MAP rat pituitary endocrine multiplex assay (RPTMAG-86K) for ACTH and BDNF and MILLIPLEX ^®^ MAP Cytokine/Chemokine Panel (RECYMAG65K27PMX, both MerckMillipore, Darmstadt, Germany) to simultaneously analyze the levels of 27 further cytokines/chemokines. Plasma samples were diluted to a protein concentration of 2 mg/ml, then analysed on a MAGPIX system (Luminex, Austin, U.S.A.) and quantified using MILLIPLEX® Analyst 5.1 software. Cytokines/chemokine measured included RANTES, GRO KC CINC-1, VEGF, Fraktalkine, LIX, MIP-2, G-CSF, Eotaxin/CCL11, GM-CSF, IL-1α, Leptin, MIP-1α, IL-4, IL-5, IL-1β, IL-2, IL-6, EGF, IL-13, IL-10, IL-12p70, IL-5, IL-17A, IL-18, MCP-1, IP-10, KC, VEGF, Fractalkine, LIX, MIP-2 and TNF-α.

### Statistical analyses

All statistical analyses were conducted using RMA gene expression data for all available time points for test, positive, and negative control, respectively, applying a two-tailed, unpaired, homoscedastic Student t-test (Microsoft ^®^ Excel ^®^ for Microsoft 365 MSO, version 2022). A *p*-value of <0.05 was considered significant.

### Ethical approval

This study was approved under protocol number BRC IACUC #151001 by the IACUC of the Biological Resource Centre (BRC, Biomedical Sciences Institutes, Agency for Science, Technology and Research (A*STAR), 20 Biopolis Way, #07-01 Centros Building, Singapore 138668) on 26 Feb 2015. A specific procedure amendment for the use of the sucrose preference test was approved on 03 Feb 2016.

## Results

### Gene expression analysis

The present pilot study was designed to identify differentially expressed genes along the HPA-axis (hypothalamus, pituitary, adrenal, and spleen) during recall of the behaviourally conditioned immune response, using a conditioning paradigm to explore efferent pathways. ^11^ As the exact time point of gene alteration was unknown, we first looked at genes with maximum average regulation across all time points (0h, +3h, +6h, +24h, and +48h post recall) in all three studied groups and then looked at neurotransmitter-related regulations in greater detail, also considering single point regulations. Each analysis per group and tissue included a minimum of three independent time points (for exact samples analysed, see Methods).

**Figure 1.**
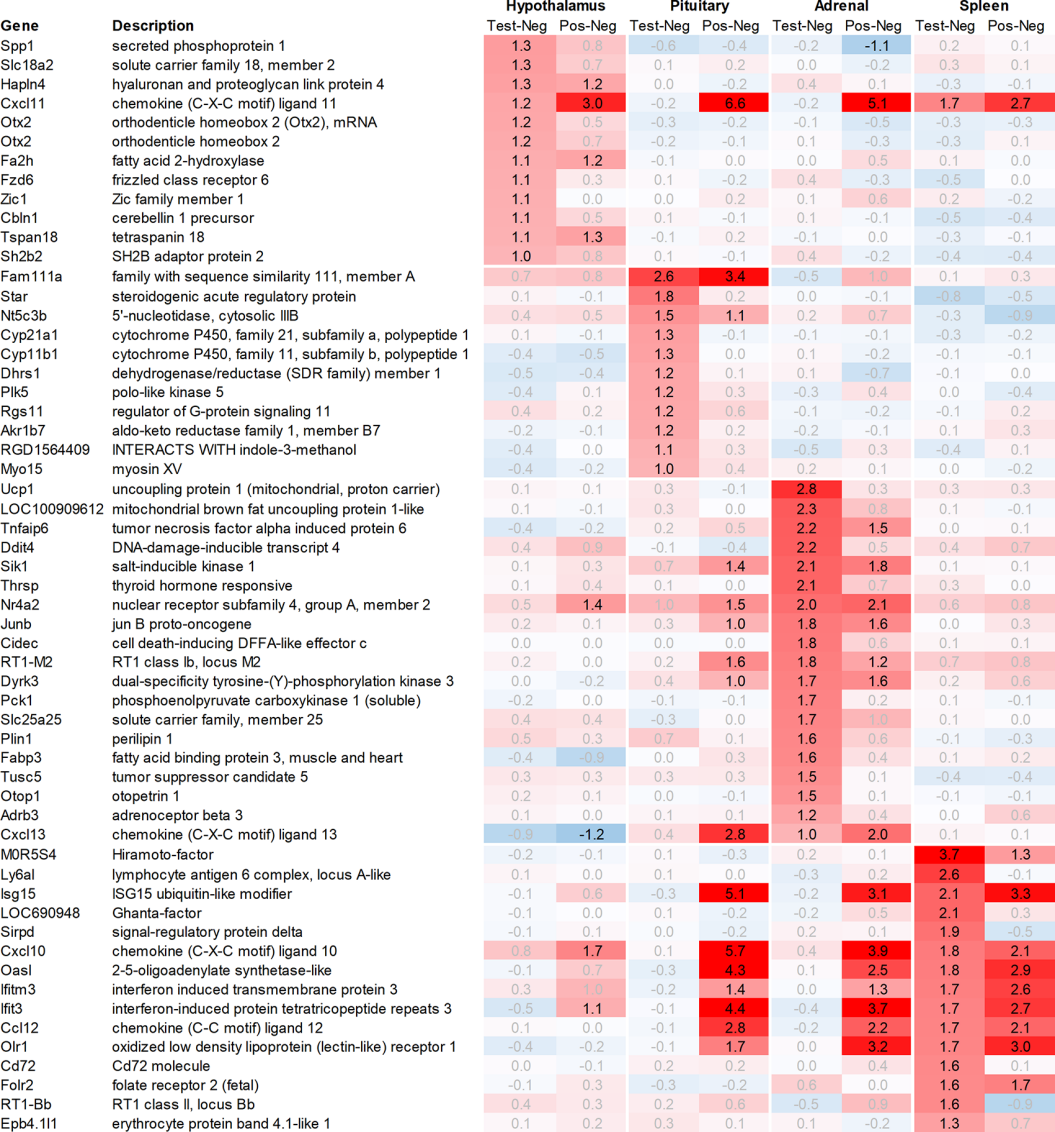
Genes upregulated across the hypothalamus, pituitary, adrenal, and spleen; differential gene expression shown as avg. across all time points for test vs. negative control and positive vs. negative control, respectively. All data shown as 2^x.

**Figure 2.**
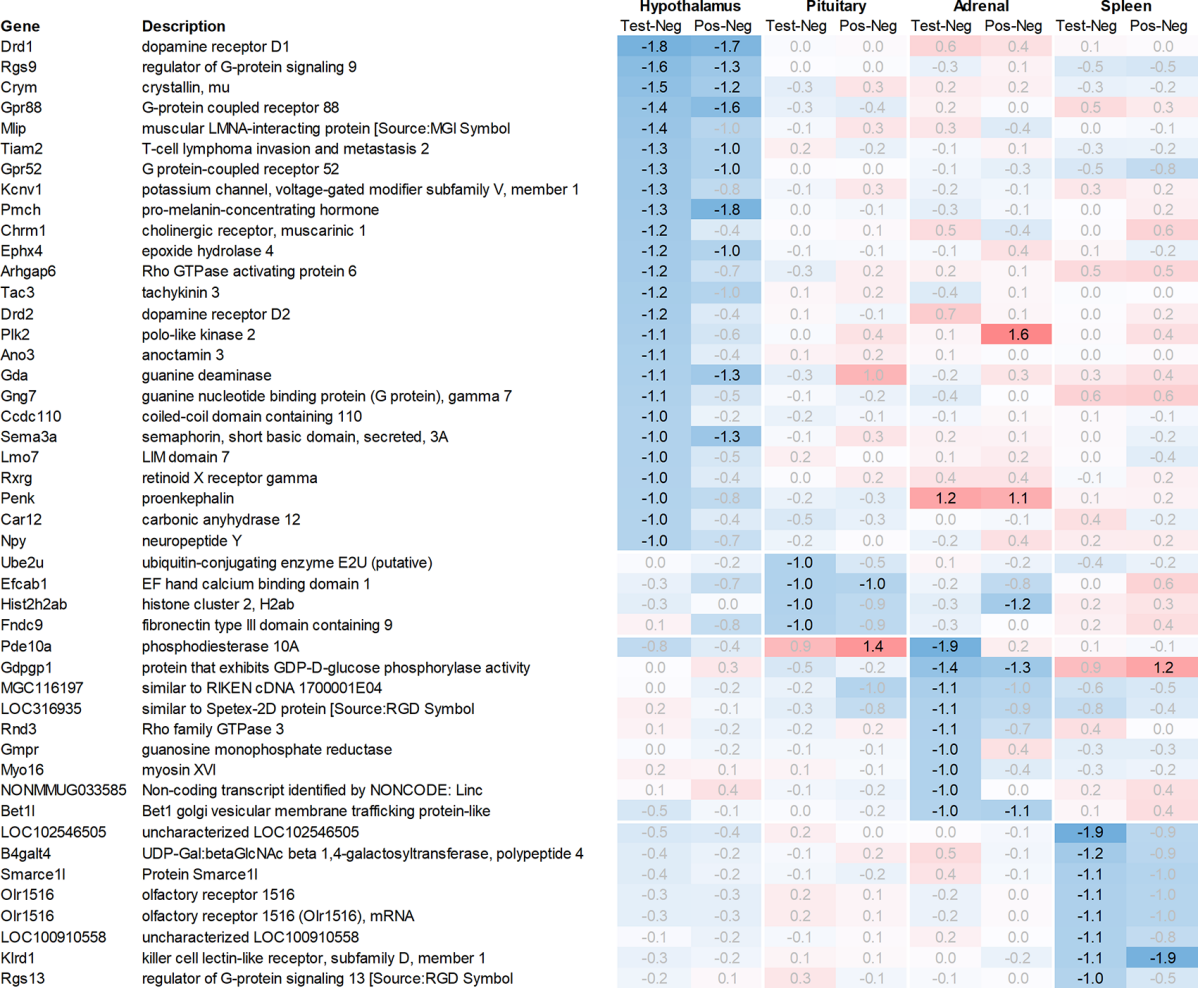
Genes downregulated across the hypothalamus, pituitary, adrenal, and spleen; differential gene expression shown as avg. across all time points for test vs. negative control and positive vs. negative control, respectively. All data shown as 2^x.

Leveraging this approach to identify major upregulated genes in test vs. negative control across all tissues with a >2fold average regulation across all time points, we identified 11 upregulated transcripts in both the hypothalamus and the pituitary ( Figure 1). In the adrenal and spleen, however, the number of genes upregulated using this approach was significantly higher. To identify meaningful candidates in these tissues, we excluded transcripts with unknown identity and focused only on genes with strong upregulation during the first six hours post recall and/or *p*<0.05, comparing expression across all time points in the test group with their respective counterparts in the negative control group, thereby reducing the number of transcripts from the adrenal glands and spleen to 18 and 20, respectively. We also excluded genes with a stronger upregulation in positive vs. negative control than the test except when also showing a strong regulation across multiple other tissues, e.g. Cxcl11 or Cxcl13. A similar approach was then applied to identify the most promising candidate genes for downregulation ( Figure 2).

In addition to this, we focused on the up- and downregulation of neurotransmitter-associated genes such as receptors, transporters, and solute carriers, potentially involved in dopaminergic, serotonergic, opioid, cholinergic, glutamatergic, or GABAergic neurotransmission. We then selected for strong regulation across multiple tissues, even if not crossing an average >2fold regulation across all time points analysed, and limited the time frame for this analysis to the first six hours post recall, unless otherwise mentioned.

### Hypothalamus

In the hypothalamus, a total of 11 transcripts, detected by 12 probes, were upregulated >2fold when comparing the average regulation across all time points in the test group vs. negative control. Among these, Spp1, which codes for secreted immune modulator osteopontin (OPN), and Slc18a2, which codes for vesicular monoamine transporter 2 (VMAT2), demonstrated the strongest upregulation in the test group vs. negative control, without a comparable upregulation in the positive vs. negative control. Likewise, Otx2, which codes for orthodenticle homeobox 2 and was detected by two independent probes, also showed a strong upregulation in the test group vs. negative control without a strong upregulation in the positive vs. negative control as e.g. observed for Hapln4 or Cxcl11 ( Figure 1).

In addition to Slc18a2, Otx2, and Spp1, 8 additional genes were found upregulated >2fold in the hypothalamus. Out of these genes, only Fzd6, Zic1, and Cbln1 exhibited a significantly higher level of upregulation in the test group vs. negative control. On the other hand, Fa2h, Tspan18, Sh2b2 and Hapln4 and Cxcl11 as already mentioned, either showed a weaker level of regulation or had a similar or even stronger regulation in the positive vs. negative control.

Both Otx2 and Spp1 have been shown to interact with Sox9 (SRY box transcription factor 9) ^23^ and like Fzd6 and Zic1, seem to play a role in Wnt/ß-catenin signaling. We, therefore, also analysed Sox-related gene expression and found that Sox7/8/9/10/11 and 13 were upregulated in the hypothalamus of the test group compared to the negative control. Notably, Sox11 showed the strongest immediate regulation post recall ( Figure 4).

Looking at downregulation ( Figure 2), among the genes with the strongest average regulation across all time points were dopamine receptors Drd1 and Drd2, inhibitory regulatory G-protein Rgs9, and Gpr88 (involved in negative regulation of opioid signaling), as well as thyroid hormone-binding protein crystallin-mu (Crym). Likewise, Gpr52, shown to have a modulatory role in dopamine receptor signaling, as well as Mlip, Tiam2, Kcnv1, Chrm1, Ephx4, Arhgap6, Tac3, Plk2, and Ano3 were strongly downregulated.

Looking in more detail at neurotransmitter-related regulation ( Figure 3a), we detected an upregulation of inhibitory glycine- (Glra1, Slc6a9, Slc6a5) and GABA- (Slc6a11, Gabra6) and excitatory glutamate-related genes (Grik3, Grik4, Grm1, Grm4, Grin2d and 3b, Slc17a6 and Slc25a18). In addition, we saw upregulation of neuropeptide S (Nps), adenylate cyclase-activating polypeptide 1 (Adcyap1) plus upregulation of nicotinic cholinergic (Chrna4, Chrna2, and Chrna6) and opioid-transmission related genes (Pnoc, Pcsk1n; detected by two independent probes). Interestingly, while this upregulation was observed with multiple genes immediately post recall in the positive control, the same upregulation was only observed +3h post recall in the test group.

**Figure 3a.**
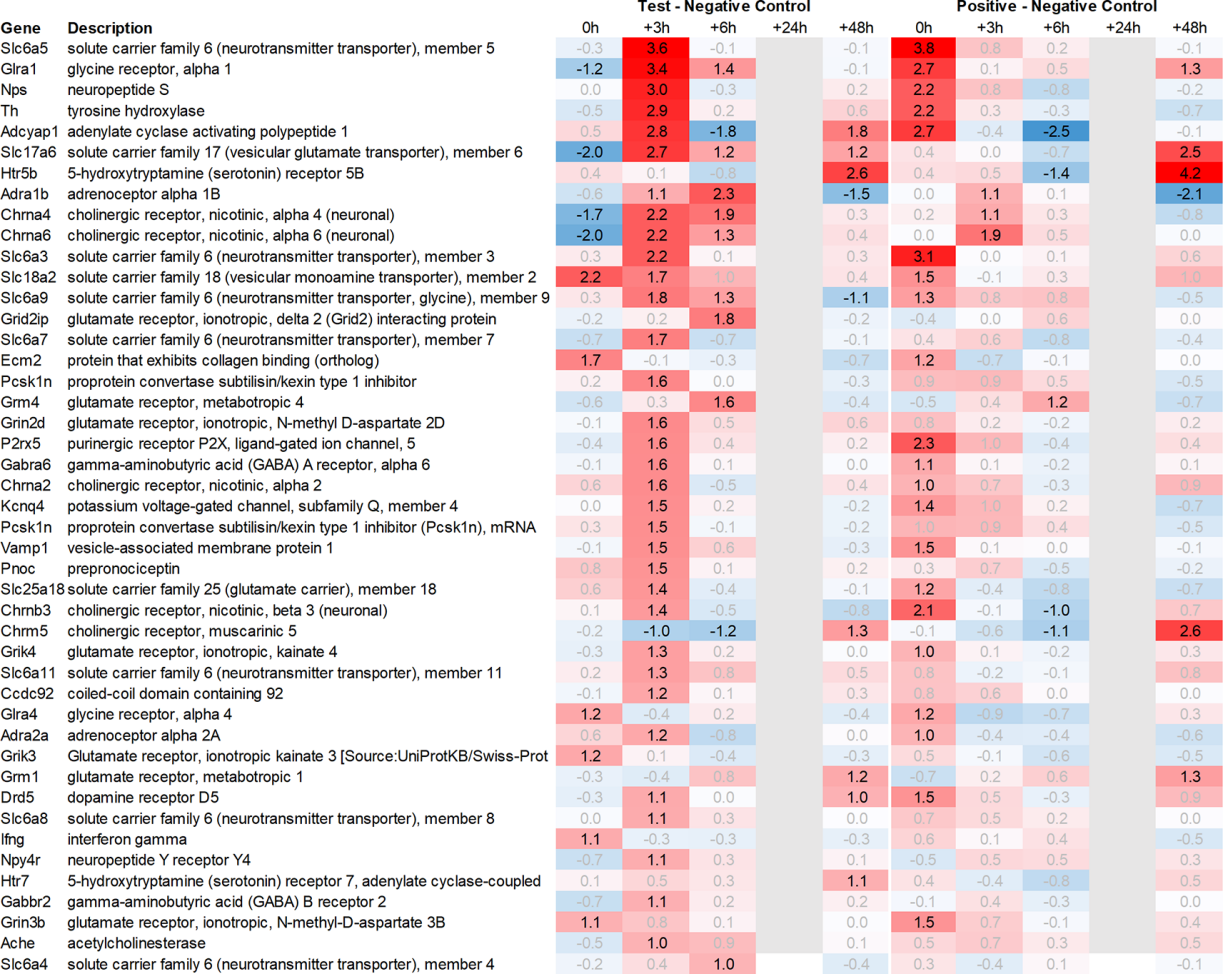
Neurotransmitter-related genes upregulated in the hypothalamus; differential gene expression at 0h, +3h, +6h, +24h, and +48h for test vs. negative control and positive vs. negative control, respectively. Grey lanes depict combinations not analyzed due to insufficient RNA quality. All data shown as 2^x.

This pattern of delayed regulation +3h in the test group vs. positive control was also seen with many downregulated, signaling-related transcripts ( Figure 3b) such as enkephalin precursor proenkephalin Penk. We also observed a downregulation of Pcsk1 and prodynorphin Pdyn, opioid receptors kappa and mu (Oprk1, Oprm1), GABAergic- (Gabra4, Gabra5, Gabrad, Gabrarq), serotonergic- (Htr2a, Htr1d, and Htr6) and muscarinic cholinergic receptors (Chrm1), opioid regulators Rgs4 and 9, and neuropeptide Y (Npy; detected by two independent probes). Interestingly, the hypothalamus of test animals immediately post recall showed more than 30fold decrease in the expression of two of the most highly regulated genes, overall: pro-melanin-concentrating-hormone Pmch and vasoactive intestinal peptide Vip.

**Figure 3b.**
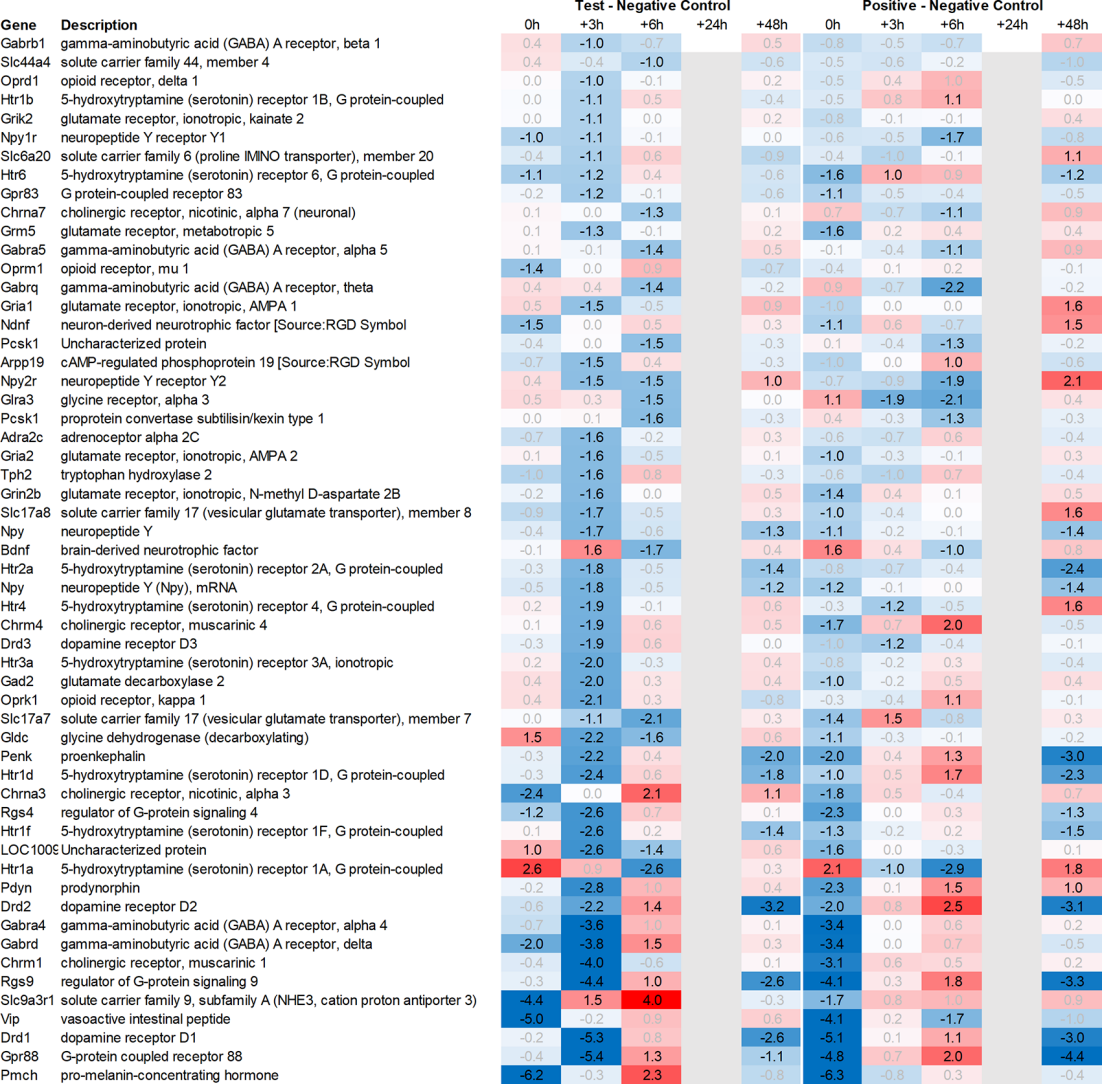
Neurotransmitter-related genes downregulated in the hypothalamus; differential gene expression at 0h, +3h, +6h, +24h, and +48h for test vs. negative control and positive vs. negative control, respectively. Grey lanes depict combinations not analyzed due to insufficient RNA quality. All data shown as 2^x.

**Figure 4.**
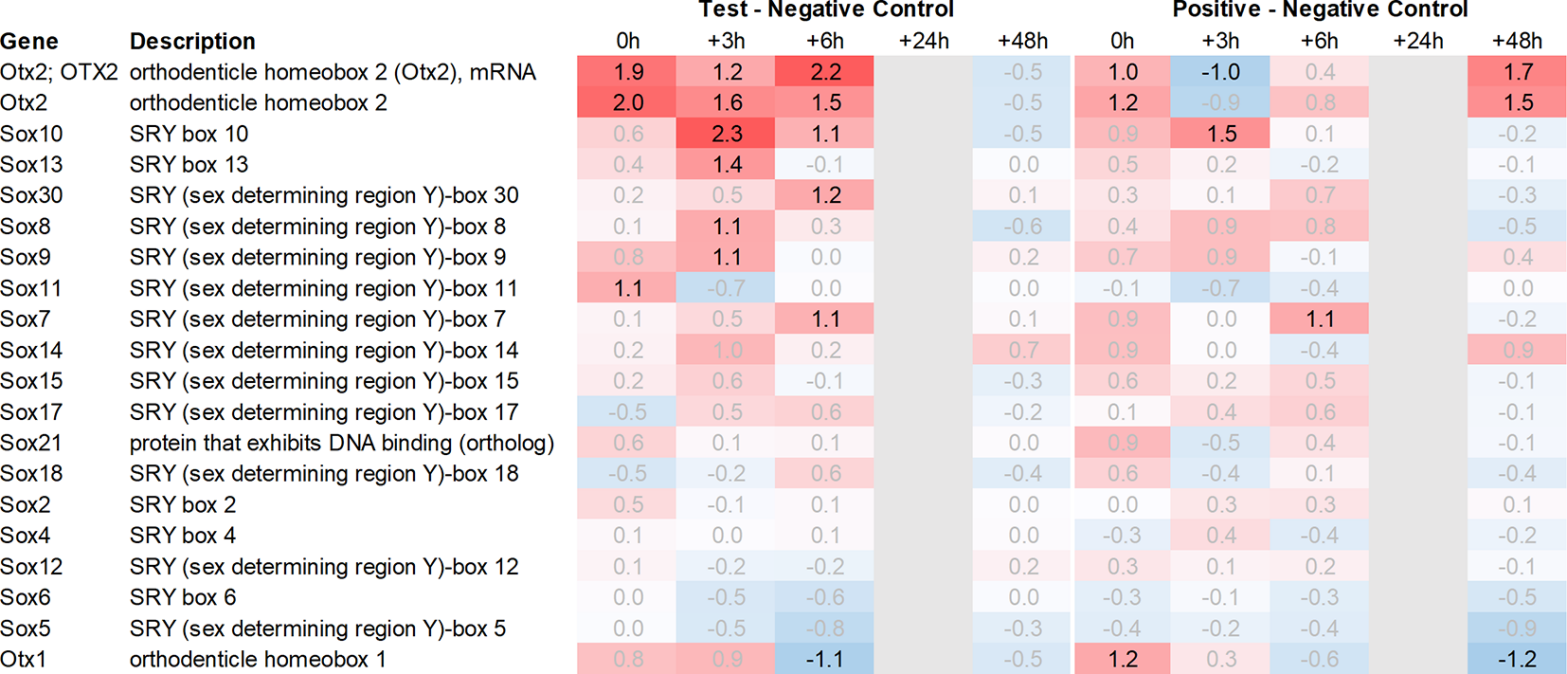
Otx and Sox genes up- and downregulated in the hypothalamus; differential gene expression at 0h, +3h, +6h, +24h, and +48h for test vs. negative control and positive vs. negative control, respectively. Grey lanes depict combinations not analyzed due to insufficient RNA quality. All data shown as 2^x.

### Pituitary

In the pituitary of the test group and positive controls, we found a heterogenous pattern of expression in steroidogenic, nuclear, and metabolism-related genes. Except for Fam111a and Nt5c3b, most of the genes like Cyp11b1/2, Plk5, unknown transcript RGD1564409, and Myo15 showed an upregulation preferentially in the test group but not in positive controls; however, as we had to exclude the first two time points from the pituitary, most of the upregulation of these genes was observed only +24h post recall. Contrary to this pattern in the test group, we detected a very strong upregulation of interferon-stimulated genes (ISGs) in the positive vs. negative control such as Cxcl9/10/11, Gbp5, Rsad2, Ccl2 and 12, Isg15, Oasb1a/b, Ifit2/3, in some cases exceeding regulations by factor 100.

Looking at downregulation, we only observed four genes with an average gene expression downregulated >2fold: ubiquitin-conjugating enzyme Ube2U, Efcab1, Hist2h2ab, and Fndc9.

With respect to neurotransmission, we detected an upregulation of ionotropic glutamate receptors like kainate receptor (Grik2) at +6h and monoamine transporter Slc18a1 (VMAT1, endocrine variant of CNS-specific and hypothalamus-upregulated VMAT2), GABA receptors (Gabbr1, Gabre) and opioid binding protein-like (Opcml) upregulation in the pituitary +24h post recall of the test group. At the same time, tryptophan hydroxylase Tph1 and GABA-related transporter Slc6a1 were downregulated at +6h post recall, while cholinergic nicotinic neurotransmission-related genes as Chrna9 and Slc44a4, neuron-derived neurotrophic factor Ndnf and proline transporter Slc6a20 were downregulated +24h post recall ( Figure 9).

### Adrenal

In the adrenal glands, 19 genes were found to be upregulated >2fold on average in the test group compared to their negative control counterparts. Most of the upregulated genes observed were involved in thermogenesis and metabolic pathways like carbohydrate metabolism (Pck1), lipid metabolism (Cidec, Thrsp), triglyceride catabolism (Fabp3), and heat-generation related functions (Ucp1; Thermogenin), which showed a strong and immediate upregulation in the test group.

In addition to the above-mentioned genes, we observed an upregulation of tumor necrosis factor signaling genes (Tnfaip6, Nr4a2), stress-responsive gene (Ddit4), gene encoding serine/threonine protein kinase (Sik1), thyroid hormone responsive protein (Thrsp), nuclear receptor (Nr4a2), proto-oncogene (Junb), and cell death-inducing DFFA-like effector c (Cidec).

Looking at downregulation, we observed 9 transcripts with an average gene expression downregulated >2fold: Phosphodiesterase (Pde01a), Gdpgp1, mgc11619, LOC31693, Rho family GTPase (Rnd3), Guanosine monophosphate receptor (Gmpr), Myosin protein (Myo16), and Golgi vesicular membrane trafficking protein (Bet1l).

Related to neurotransmission in the adrenal glands of test animals, we found an immediate upregulation of adrenergic receptors (Adra1a, Adrb3), neuropeptide Y receptor (Npy4r), purinergic signaling receptor (P2rx5) as well as cholinergic nicotinic receptor (Chrna7). We also observed an upregulation of endorphin precursor proenkephalin (Penk), opioid receptor mu (Oprm1), neuron-derived neurotrophic factor (Ndnf), and dopamine receptor (Drd2) ( Figure 10). We further saw an upregulation in the inhibitory neurotransmission-associated genes like GABA receptors (Gabrg1, Gabrg2), excitatory neurotransmitter glutamate/D-serine amino acid transporter (Slc1a4), and muscarinic cholinergic receptor 1 (Chrm1). Moreover, we observed single-point downregulation for opioid-related convertase (Pcsk1/2), vesicular glutamate transporter (Slc17a6), GABA transporters (Slc6a1, Slc6a11), and serotonin receptor (Htr3a).

### Spleen

While analysing the gene expression in the spleen, the expression of 15 transcripts was found to be upregulated on average >2fold across all time points measured in the test group as compared to negative controls ( Figure 1). These genes included Ig-like domain-containing protein MOR5S4 (“Hiramoto factor”), lymphocyte antigen 6 complex (Ly6al), ubiquitin-like modifier (Isg15), similar to paired-Ig-like receptor A1 LOC690948, signal regulatory protein delta (Sirpd), chemokines Cxcl10 and Ccl12, oligoadenylate synthetase (Oasl), interferon signaling molecules as Ifitm3 and Ifit3, oxidized low-density lipoprotein (Olr1), B-cell receptor Cd72, folate receptor 2, RT-1BP, and erythrocyte protein band Ebp4.1. Most of the upregulated genes identified in the spleen were either involved in immune activation or regulation.

Likewise, many genes downregulated in the spleen across all time points measured were related to immune regulatory functions such as G protein signaling regulator (Rgs13) and Killer cell lectin receptor (Klrd1). In addition, we observed a strong downregulation of olfactory receptor Olr1516, detected by two probes, Smarce1l, UDP-Gal:betaGlcNAc beta 1,4-galactosyltransferase polypeptide 4 (B4galt4) and two further uncharacterized transcripts (LOC10254, LOC10091).

Looking at gene expression regarding neuronal and endocrine signaling ( Figure 11), we detected an upregulation of opioid signaling (Pcsk2 and Pcsk1; detected by two independent probes and opioid receptor Sigmar1), nicotinic acetylcholine receptor alpha 3 (Chrna3), ionotropic glutamate receptor delta 2 (Grid2), DNA directed RNA polymerase II (Polr2m), nmDA glutamate receptor ionotropic 1A (Grin1a), D-serine transporter (Slc1a4) and glutamate decarboxylase 2 (Gad2). We also observed downregulation of opioid receptor delta 1 (Oprd1), adrenomedullin 2 (Adm2), cholinergic receptor muscarinic 2, and both glutamatergic (Grik1, Gad2, Grm4), GABAergic (Gabbr1, Gabrp) and serotonergic receptors (Htr1d). Interestingly, we observed an upregulation of neuropeptide Y (Npy) and a downregulation of neuropeptide S (Nps) in the spleen, which is exactly the opposite of what was seen in the hypothalamus.

Taken together, gene expression data obtained from the spleen were summarized by a strong upregulation of immunomodulatory genes, chemo- and cytokines, and glutamatergic as cholinergic neurotransmission related genes. Similar to hypothalamus, we saw a pattern of immediate regulation in the spleen for positive control vs. negative control of opioid-related transcripts, such as Pcsk1 or Gpr88, while the same upregulation in the test group vs. negative control was only observed 3h later.

### Validation of microarray gene expression data by using qRT-PCR

**Figure 5.**
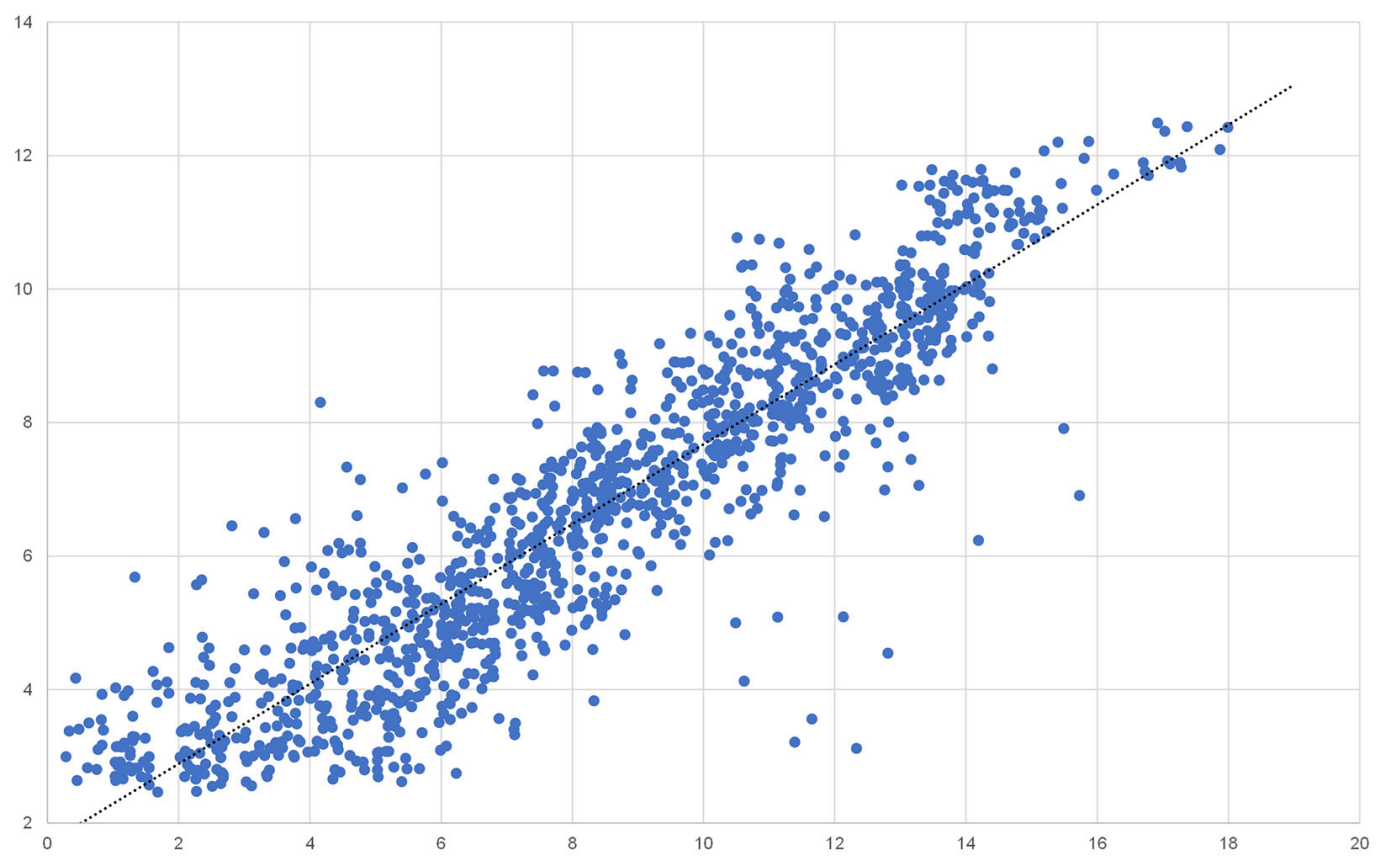
Scatter plot correlation analysis of 32 differentially expressed genes in all four tissues analyzed using Affymetrix chip analysis and qRT-PCR.

To validate the gene expression results obtained by rat full genome microarray analysis, we picked a representative subset of 32 genes including housekeeping genes, interleukins, ISGs, and chemokines, preferably regulated across multiple tissues ( Table 2). ^24^ The mRNA quantity of those genes was analysed by qRT-PCR and correlation with the corresponding microarray results was assessed. While not all genes correlated perfectly, a strong overall correlation across multiple tissues and expression levels (r ^2^ = 0.8377) was detected ( Figure 5).

**Table 2.** List of 32 genes used for validation of Affymetrix rat genome chip data by qRT-PCR including individual correlation per gene and correlation across all data points.

Gene symbol	Official full name	Correlation
Ccl12	chemokine (C-C motif ) ligand 12	0.840
Ccl2	chemokine (C-C motif ) ligand 2	0.859
Ccl5	chemokine (C-C motif ) ligand 5	0.929
Ccl7	chemokine (C-C motif ) ligand 7	0.821
Cd274	CD274 molecule	0.914
Creb3l1	cAMP responsive element binding protein 3-like 1	0.944
Csf1	colony stimulating factor 1 (macrophage)	0.829
Cx3cl1	chemokine (C-X3-C motif ) ligand 1	0.877
Cxcl10	chemokine (C-X-C motif ) ligand 10	0.890
Cxcl11	chemokine (C-X-C motif ) ligand 11	0.872
Cxcl9	chemokine (C-X-C motif ) ligand 9	0.851
Fos	FBJ osteosarcoma oncogene	0.898
Fosl2	fos-like antigen 2	0.962
Gbp2	guanylate binding protein 2, interferon-inducible	0.926
Gbp5	guanylate binding protein 5	0.866
Hprt1	hypoxanthine phosphoribosyltransferase 1	0.783
Igtp	interferon gamma induced GTPase	0.939
Il10	interleukin 10	0.907
Il1a	interleukin 1 alpha	0.734
Il1b	interleukin 1 beta	0.913
Il21	interleukin 21	0.347
Irf1	interferon regulatory factor 1	0.865
Irf7	interferon regulatory factor 7	0.954
Junb	jun B proto-oncogene	0.914
Ly49si1	immunoreceptor Ly49si1	0.647
Mmp12	matrix metallopeptidase 12	0.906
Mx2	myxovirus (influenza virus) resistance 2	0.947
Rsad2	radical S-adenosyl methionine domain containing 2	0.928
RT1-A1	RT1 class Ia, locus A1	0.859
Sdha	succinate dehydrogenase complex, subunit A, flavoprotein	0.896
Tnf	tumor necrosis factor	0.759
Usp18	ubiquitin specific peptidase 18	0.932
**Total**		**0.914**

### Plasma cytokine/chemokine levels post recall of the behaviorally conditioned immune response

**Figure 6.**
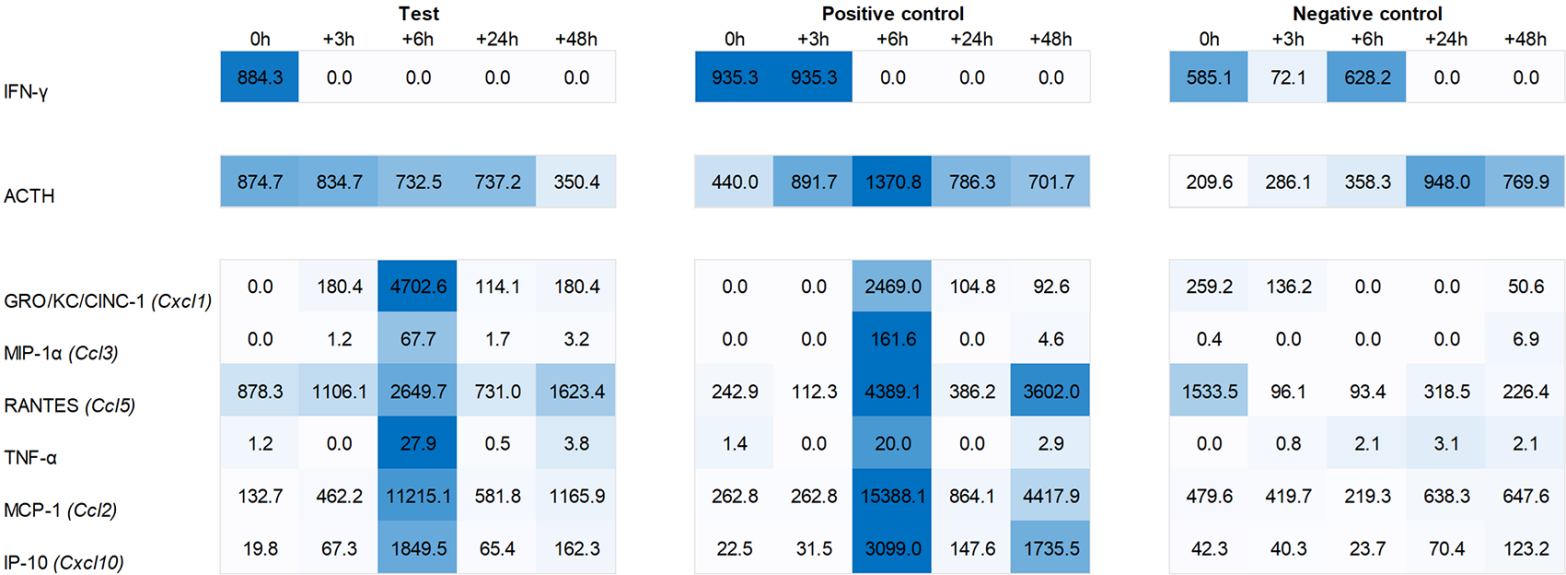
Heat map showing levels of IFN-γ, ACTH, GRO/KC/CINC-1, MIP-1α, RANTES, TNFα, MCP-1, and IP-10 in the plasma of test, positive control, and negative control animals.

Plasma levels of ACTH were found to be increased during the first six hours post recall in both test group and positive control animals, with a peak at +6h in the latter. Similarly, we observed an increased plasma level of IFN-γ immediately post recall in the test group and 0h to +3h in the positive control. IL-1α showed a similar yet less pronounced response in the plasma of both test and positive control animals, peaking at 0h and +6h post recall while IL-1β showed a similar profile to IFN-γ, peaking at 0h to +3h in the test and three hours later in the positive control. The most pronounced changes were observed for the plasma levels of chemokines GRO/KC/Cinc-1 (Cxcl1), IP-10 (Cxcl10), MCP-1 (Ccl2), MIP-1α (Ccl3), Rantes (Ccl5), and TNF-α, with a strong peak at +6h post recall, both in test group and positive control animals ( Figure 6).

### Sucrose preference test (SPT) and anhedonia

Regarding future studies, we also assessed behavioural data during recall of the conditioned immune response employing the sucrose preference test to evaluate potentially conditioned depression-like anhedonia. During the run-in phase from day -7 to -1, a constant and strong preference (>90%) for the sucrose solution was observed in all study groups. As shown in Figure 12, we observed a drop in sucrose preference in both the test group and the positive control compared to D-1 after poly I:C injection; the reduction was much less pronounced in the negative control, receiving only saline injections. Following a return to normal on D1, we observed another drop in all three groups on D2 with further reduction in sucrose preference in all animal groups, potentially due to the repeated injection and handling stress ( Figure 12).

**Figure 7.**
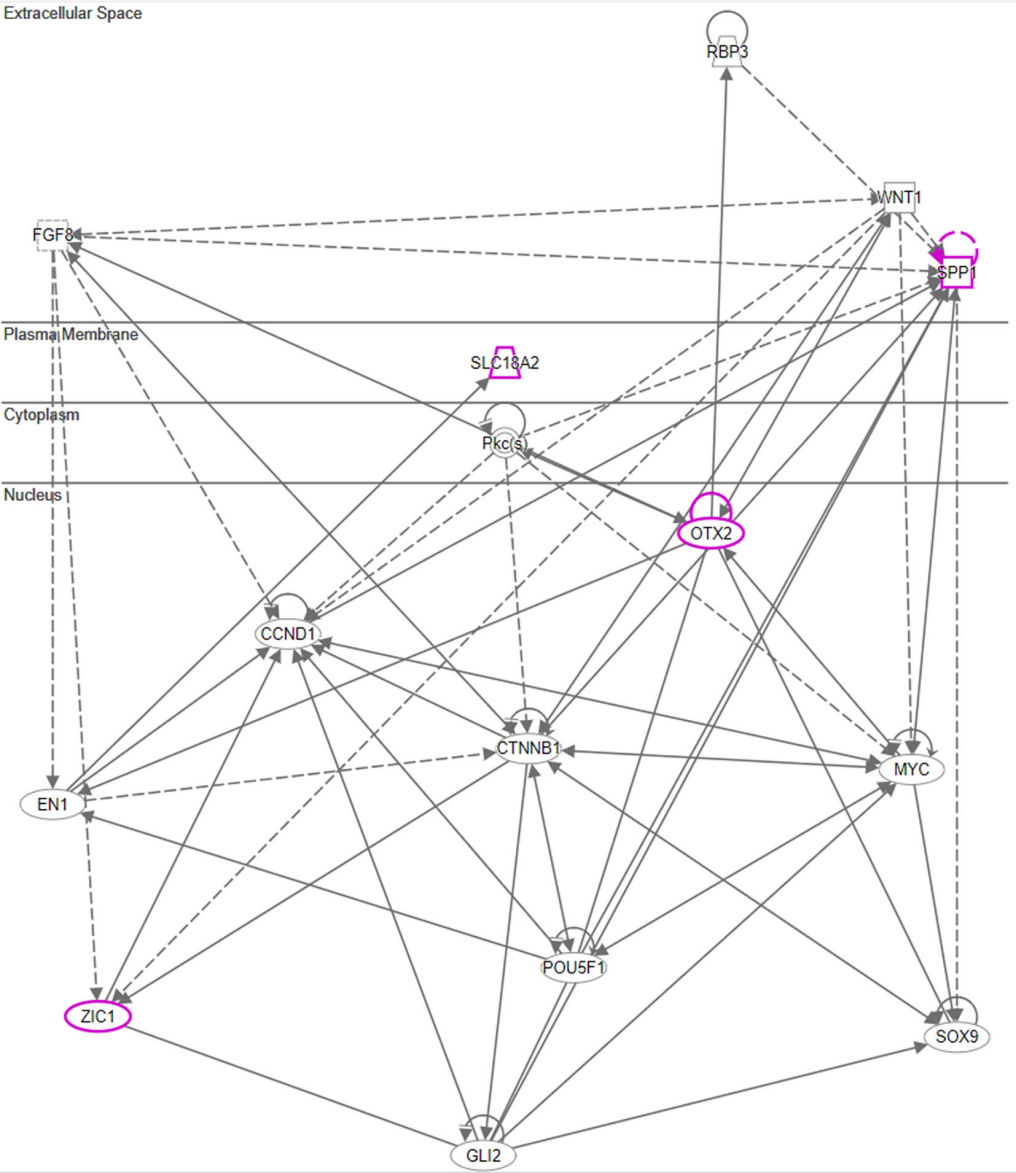
Network chart, depicting potential interaction between upregulated genes connected to Otx-2, Spp1, and Slc18a2 in the hypothalamus and potential intermediaries (Qiagen 2020-2022 Ingenuity Pathway Analysis).

**Figure 8.**
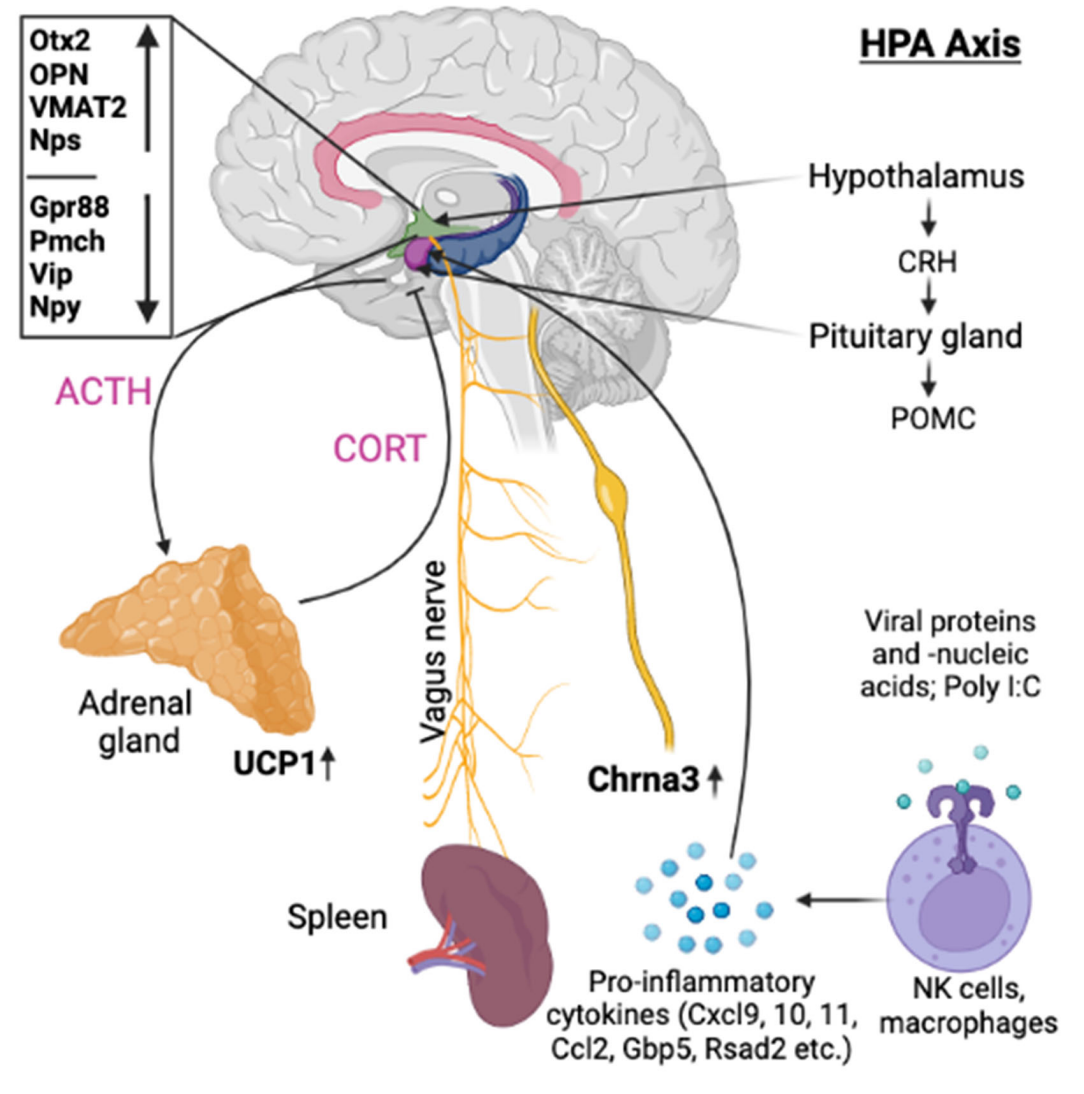
Illustration showing the possible efferent pathway involved in the immune regulation during recall of behaviorally conditioned immune response.

**Figure 9.**
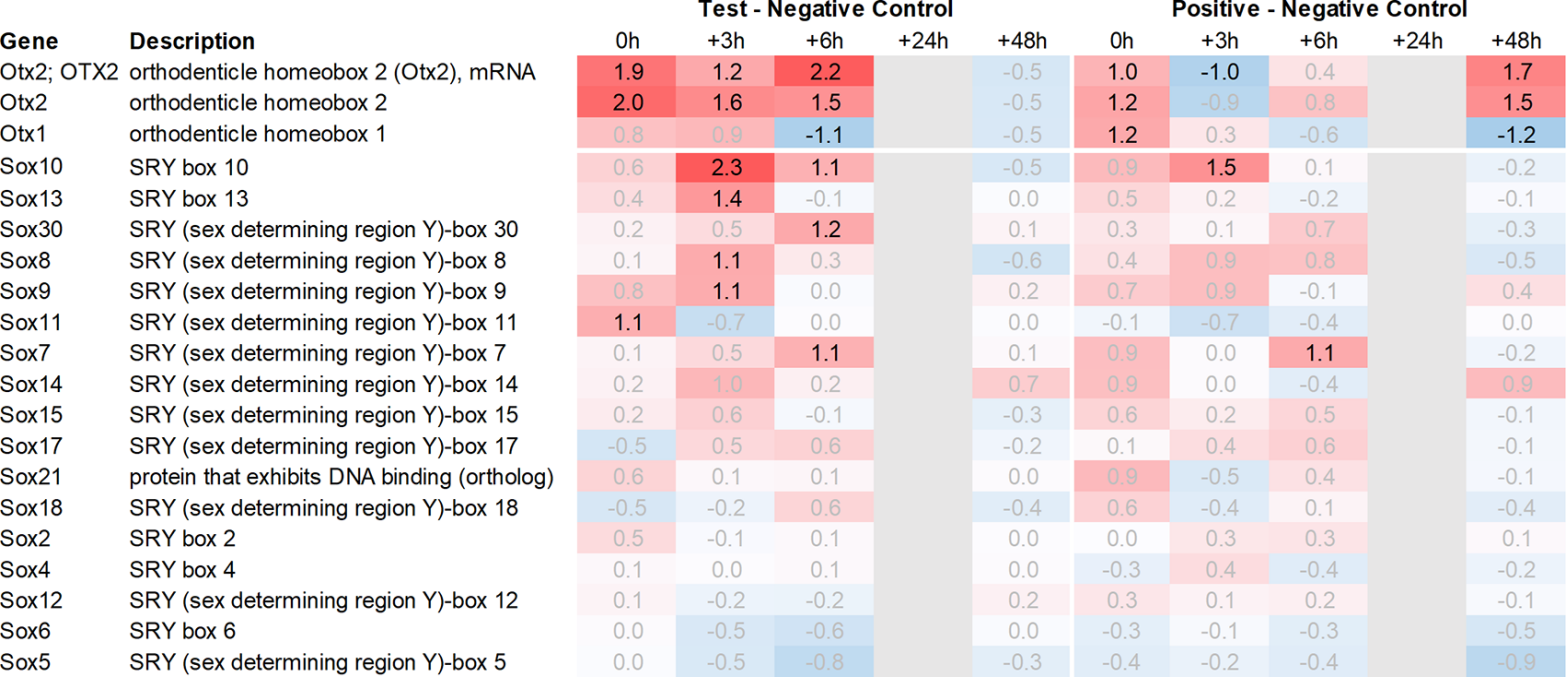
Neurotransmitter-related genes upregulated in the pituitary; differential gene expression at 0h, +3h, +6h, +24h, and +48h for test vs. negative control and positive vs. negative control, respectively. Grey lanes depict combinations not analyzed due to insufficient RNA quality. All data shown as 2^x.

**Figure 10.**
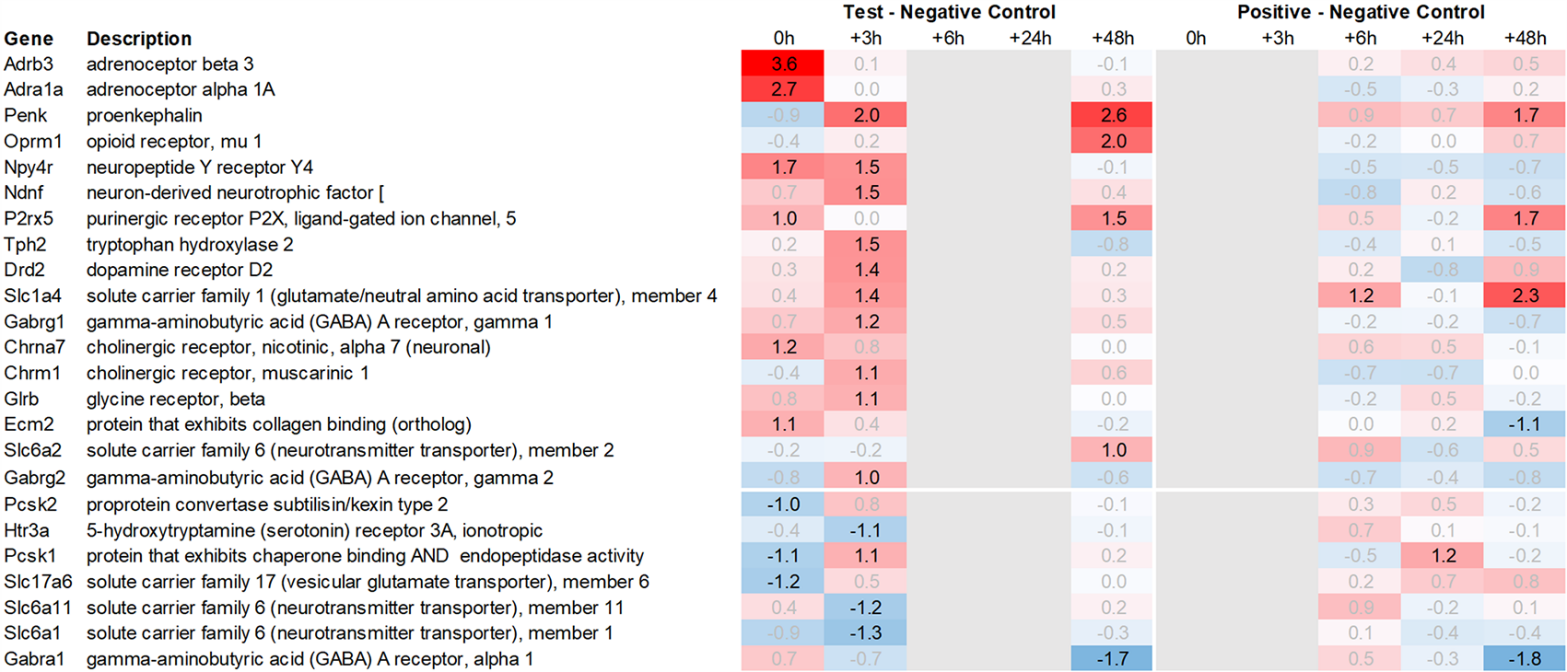
Neurotransmitter-related genes upregulated in the adrenal; differential gene expression at 0h, +3h, +6h, +24h, and +48h for test vs. negative control and positive vs. negative control, respectively. Grey lanes depict combinations not analyzed due to insufficient RNA quality. All data shown as 2^x.

**Figure 11.**
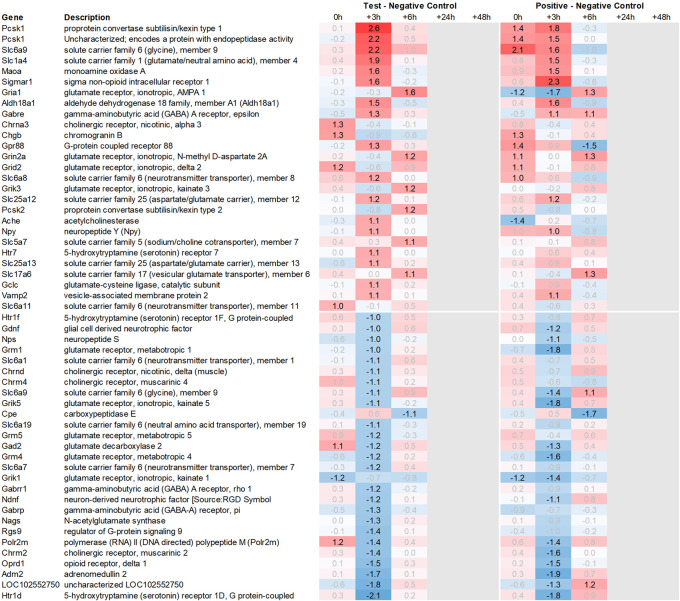
Neurotransmitter-related genes upregulated in the spleen; differential gene expression at 0h, +3h, +6h, +24h, and +48h for test vs. negative control and positive vs. negative control, respectively. Grey lanes depict combinations not analyzed due to insufficient RNA quality. All data shown as 2^x.

**Figure 12.**
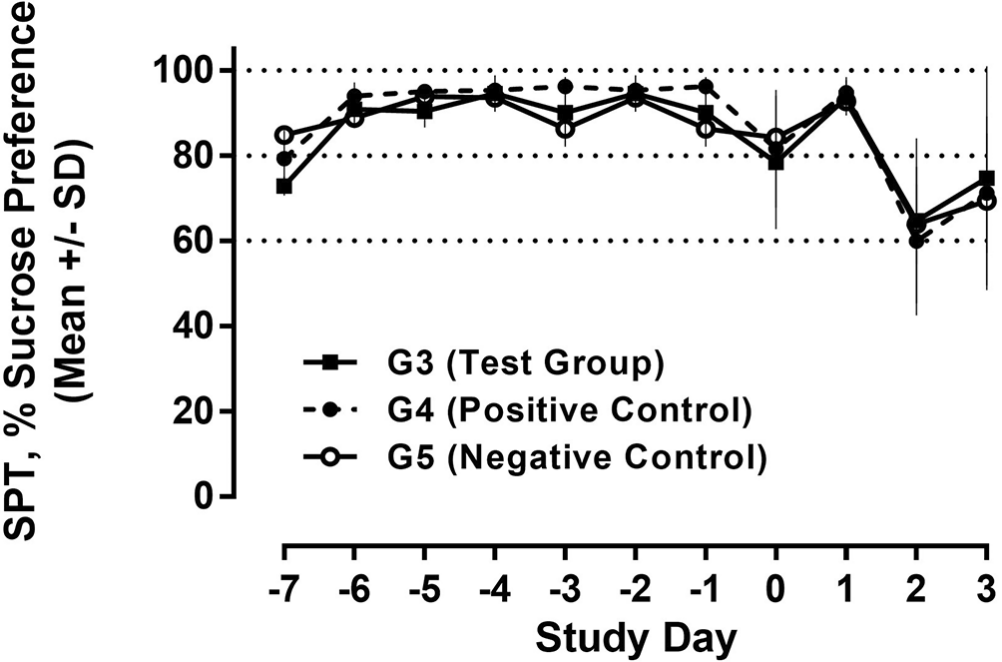
Sucrose preference test (SPT). Water and 2% sucrose solution were presented to all animals, and the relative consumption of sucrose solution is shown as % (100% = exclusively sucrose solution). The consumption over 24h was measured and analysed for each day.

## Discussion

In this pilot study, we aimed to explore the molecular mechanisms that underlie behaviourally conditioned immune enhancement. For that purpose, we employed a conditioning paradigm that involved using camphor smell as CS and an i.p. injection of poly I:C as US. We hypothesized that the efferent signals for the recall of immune responses originate from the hypothalamus. To test our hypothesis, we analysed differential gene expression in the organs along the HPA axis in test animals (who underwent smell re-exposure and sham injection on D2), positive control animals (who received poly I:C injection on D0 and D2, only), and negative control animals (who underwent smell re-exposure but received sham injections both on day 0 and day +2). Additionally, we analysed the plasma levels of cytokines/chemokines.

After analysing gene expression patterns in the hypothalamus, pituitary, adrenal glands, and spleen, we found evidence that there could be multiple signaling pathways post recall along the HPA axis. One example is the involvement of the WNT/β-catenin pathway, including Otx2, Fzd6, Zic1, and Sox7/8/9/10/11 and 13 as identified in the hypothalamus during the early phases of the recall. Moreover, we also observed elevated expression of secreted immunomodulators (osteopontin, OPN) in the hypothalamus, as well as dopaminergic and opioid signaling, corroborating earlier and recent studies. ^25^^,^^26^ In the pituitary of test animals, we observed a strong upregulation of steroid synthesis related genes. Although the gene expression of interferon stimulated genes (ISGs) as Cxcl10, Cxcl9, Ccl2, Ccl7, Ccl12, Gbp5, Rsad2, Oasb1a/b, and Ch25h seemed mostly confined to the positive control, some of these genes as Cxcl11 were also found upregulated in the test group e.g. in hypothalamus and spleen. We also identified a significant and immediate increase in the expression of genes related to thermogenesis and prostaglandin synthase in the adrenal glands. Additionally, we noticed a strong increase in the expression of immunoregulatory molecules in the spleen. The molecular signal appears to travel from the hypothalamus to the pituitary gland, and then onwards to the adrenal gland and spleen, both via neuronal pathways as blood-borne messengers. From there a feedback loop seems to bring back the signals to the hypothalamus, resulting in delayed gene expression of additional genes. In line with this concept, in the positive control, a similar gene expression pattern could be observed; however, the reaction seemed to occur much faster, especially for downregulated genes like Npy, Gpr88, or Penk. These genes were present three hours earlier in the positive control where poly I:C was re-injected, as opposed to their gene expression in the test group post re-exposure to the conditioned stimulus.

Upon closer examination of the cascade, one key player in the hypothalamus appears to be Otx2. This is due to its immediate and strong upregulation in the test arm, which was detected by two independent array probes. Moreover, the upregulation of frizzled receptor Fzd6 and transcription factor Zic1 in the test arm further corroborate the involvement of the WNT/β-catenin pathway. While Wnt1 upregulation in the test arm did not exceed the >2fold threshold, we observed an immediate upregulation of Wnt5a and Wnt7b in the hypothalamus of test animals. This observation of Wnt regulation in association with genes involved in dopaminergic signal transmission (Slc18a2) is consistent with a recent study by Zhang & Yang, 2021, ^27^ which found two other Wnt genes, Wnt1 and Wnt3a play a critical role in the differentiation of neural stem cells into dopaminergic neurons using tetrahydroxy stilbene glycoside and Wnt-signaling specific inhibitor (IWR1).

Downstream of Otx2, we noticed an increase in the expression of several neural transcription factors, such as Sox8, 9, 10, 11 and 13. Sox11 was upregulated immediately post recall in the test group, while Sox10 showed the most significant upregulation +3h post recall. Of note, Sox9, 10, and 11 have previously been shown to interact with Otx2 during the neural development ^28^ and regulation of visual cycle gene expression in the retinal pigment epithelium. ^23^ In addition, we observed a single point upregulation of the homeobox transcription factor Engrailed 1 (En1) +3h post recall in the test group, and immediately post poly I:C re-injection in the positive control. Engrailed 1 has been shown to interact with Otx2 and inhibit the canonical Wnt-signaling during murine embryonic development of the mid and hindbrain. Its expression seems to play a crucial role in the survival of both serotonergic and dopaminergic neurons. ^29^

In addition to Otx2, two other genes exhibited strong upregulation in the hypothalamus post recall in the test group: Spp1, responsible for encoding the immune regulator osteopontin (OPN), and Slc18a2, responsible for encoding the vesicle transporter VMAT2. Beyond its historical role in biomineralization, OPN has been shown to hold a central role in immune activation and regulation, for example through its interactions with leukocyte integrin receptors (α4β1, α9β1, and α9β4) ^30^^,^^31^ as well as the cell-surface marker CD44, ^32^ which is involved in lymphocyte activation, recirculation, and homing. OPN is also a chemoattractant for neutrophils and expressed by a wide range of immune cells. It has been shown to play a role in immunomodulation by blocking IL-10 in Th2 type cytokine responses and promoting inflammatory IL-12 and Th1 type expansion. ^33^^,^^34^ Considering its humoral immunomodulatory functions and its pronounced upregulation during the first six hours post recall in the test group, we postulate that OPN plays a central role in the recall of the conditioned immune enhancement, either directly or in conjunction with the release of Corticotropin-Releasing Hormone (CRH) and/or other soluble factors into the median eminence.

As next step along the HPA axis, the release of CRH then triggers the release of ACTH and further immunomodulatory molecules from the pituitary gland. While this step aligns with earlier findings by Hsueh et al., ^11^ which demonstrated the involvement of plasma ACTH in the recall of the behaviorally conditioned immune enhancement, except for a short peak in the positive control at +3h, we did not detect any upregulation of Pomc, precursor of ACTH and multiple other peptide hormones, at the mRNA level in any of the tissues. We did, however, detect an immediate, single-point upregulation of IFN-γ gene expression in the hypothalamus of the test group and a similar upregulation of IFN-α in the spleen of positive control animals at +6h post recall.

An alternative, not necessarily mutually exclusive explanation, for OPN’s involvement in the signaling cascade during the recall of the conditioned immune reaction, could be the HPA feedback loop. According to Wang et al., ^35^ OPN -/- mice exhibited decreased levels of ACTH and elevated levels of cortisol during chronic restraint stress. It is speculated that this could be due to a negative feedback loop involving OPN. Interestingly, we also detected decreased levels of Cxcl10 and Ccl2 in both the hypothalamus of the test group at +6h post recall, and immediately post recall in the pituitary of positive control animals. Further to this hypothesis, Trinh et al. in 2020 ^36^ demonstrated that stress, induced by systemic poly I:C injection, can increase plasma OPN, triggering the release of ACTH. This is in line with the observation by Wang that OPN injection partially restores the ACTH response to stress in OPN -/- mice. Furthermore, an anti-OPN antibody was shown to partially inhibit the stress response in OPN WT mice. ^35^

The third strongly upregulated gene post recall in the hypothalamus of the test group was Slc18a2, encoding vesicular monoamine transporter (VMAT2). VMAT2 is responsible for transporting monoaminergic neurotransmitters such as norepinephrine and dopamine into synaptic vesicles. Interestingly, both Reserpine and 6-Hydroxydopamine (6-OHDA), which irreversibly inhibits VMAT2 and inhibits dopamine signaling respectively, have been shown to downregulate Slc18a2 transcription. This inhibition blocks both the conditioned recall of an enhanced NK reaction ^37^ and the conditioned recall of the enhanced neutrophil reaction. ^12^ According to Hsueh et al., ^11^^,^^12^^,^^38^ both cholinergic and serotonergic pathways in the CNS seem to be involved during acquisition as well as recall of the conditioned NK cell response. Additionally, this process involves both muscarinic and nicotinic receptors. In line with these data, we observed decreased activity of dopamine receptors (Drd1, Drd2) and serotonergic receptors (Htr1f, Htr2a) in the hypothalamus of test animals +3h post recall. These results suggest a possible involvement of dopaminergic and/or noradrenergic signaling pathways during the recall of the conditioned response. Figure 7 illustrates the putative interaction between the upregulated genes and their potential intermediaries.

In addition to upregulation, several genes were also found to be strongly downregulated in the hypothalamus, including pro-melanin-concentrating hormone (Pmch), a precursor of the orexigenic peptide MCH. Additionally, the genes responsible for producing vasoactive intestinal peptide (Vip) as well as neuropeptide Y (Npy), were found to be downregulated, while the gene responsible for producing neuropeptide S (Nps) was upregulated. MCH, in addition to its role in appetite stimulation, has been shown to play a critical role in regulating dopamine signaling by suppressing DA release in the nucleus accumbens. Meanwhile, NPY similarly inhibits dopaminergic neurons in the ventral tegmental area (VTA). ^39^ Neuropeptide S, on the other hand, has the exact opposite effect of NPY, acting as an anxiolytic and anorectic agent. The immediate and strong downregulation of Pmch and Vip, further augmented by downregulation of Npy and upregulation of Nps, observed immediately in the positive control and at +3h post recall in the test group, add to the pattern of appetite-reduction and energy conversation.

We also observed a strong downregulation of opioid inhibitory G-protein coupled receptor 88 (Gpr88) and regulator of G-protein signaling 9 (Rsg9) in concert with an upregulation of prepronociceptin (Pnoc) and proprotein convertase subtilisin/kexin type 1 inhibitor (Pck1n), which was detected by two independent array probes; just like with Npy and Nps, this regulation was observed at +3h in the test group and immediately in the positive control. All these observations are again in line with previous studies by Hsueh et al., ^40^ which have demonstrated the involvement of opioid pathways during the recall of behavioral conditioned immune enhancement.

Finally, we also identified that RT-1A was upregulated in both the hypothalamus and adrenal glands of test animals. RT-1A is a classical rat MHC class Ia gene that is involved in the presentation of peptides to CTLs. It also interacts with the Ly-49 family of receptors on NK cells to identify itself, thereby providing protection against NK cell lysis. ^41^

Most of the other genes we observed that were regulated in the hypothalamus post recall, such as cilia and flagella associated protein (Cfap43), involved in olfactory detection, Npas4, a key regulator of GABAergic synapse development, and dynein subunits Dnah12 and Dnah1, are indicative of a heavy involvement of microtubular transport processes during the recall of the conditioned immune reaction.

From the pituitary onward, we noted an upregulation of steroidogenic, nuclear, and metabolism-related genes like Fam111, Star or Rgs1 in test animals post recall, contrasting with a significant upregulation of ISGs in the positive control. This perspective, however, might be skewed since we were unable to analyse time points 0h and +3h in the pituitary. Consequently, we might have overlooked potential upregulation during the initial three hours.

Independently of this, we observed an upregulation of orphan nuclear receptors Nr4a2 and Nr4a3 in the test group. While the exact function of both receptors has not been fully elucidated, recent studies suggest a role in antigen presentation and viral response. ^42^ Downregulation, in contrast, did not exhibit a consistent pattern. Apart from a few isolated instances, such as Tetraspanin (Tspan8) and Claudin (Cldn1), the only notable pattern was observed with serotonin-related tryptophan hydroxylase Tph1. This mixed expression pattern in the test group stands in stark contrast to the clear and strongly ISG-dominated expression pattern in the positive control. Genes such as Cxcl9, 10, 11 or Gbp5, Rsad2, and Ccl2 showed a more than 50fold upregulation in the positive control, indicating a different mechanism when comparing poly I:C re-injection to the recall of the conditioned immune reaction.

Further along the HPA axis, the marked immediate upregulation of Ucp1 (>300fold) in the adrenal glands of test animals suggests a dramatic shift in metabolism toward heat generation, e.g. as needed for fever induction. Similarly, the strong upregulation of Thrsp has been demonstrated to play a role in the thermogenesis of brown adipocytes, ^43^ a finding recently confirmed in fed and refed hatchling chicks. This metabolic shift toward heat generation aligns with the upregulation of fatty acid binding protein Fabp3, which is implicated in the regulation of mitochondrial thermogenesis and temperature homeostasis. Concurrently, we saw the upregulation of both adrenergic receptors Adr1a and Adr3b. These receptors have been implicated in metabolic abnormalities and associated diseases. Mutations in these receptors have been speculated to be linked to weight gain in association with antipsychotic drugs, further supporting the notion of gene expression adaptation to metabolic changes in sickness-related conditions.

In the spleen, we observed a robust upregulation of both known and partially unknown immunomodulatory genes. The most significant upregulation was identified in a 354bp uncharacterized transcript, coding for a 118aa Ig-like domain containing protein (M0R5S4; “Hiramoto factor”). Reactome analysis suggests the involvement of this protein in immunoregulatory interactions between lymphoid and non-lymphoid cells, potentially through lymphoid-expressed Fc-gamma receptors and/or signaling of the B cell receptor. The second upregulated gene, Ly6aI, also known as stem cell antigen 1 (SCA-1), has recently attracted attention for its role in facilitating the transport of neurotropic vector adeno-associated virus serotype 9 across the blood-brain barrier in C57BL/6J mice. ^44^ Its classic function, however, is better known in hematopoiesis where Sca1 serves as one of the most common cell surface markers used to enrich adult hematopoietic stem cells. Furthermore, a regulatory role of Ly6 proteins in nicotinic acetylcholine receptors (nAChRs) has been suggested. ^45^

In addition to M0R5S4 and Ly6aI, numerous other upregulated genes in the spleen belong to the category of immunomodulation and regulation. RT1-Bb is a member of the MHC class II family known to interact with CD4 receptors on helper T cells. SIRPδ (Sirpd), belonging to the signal regulatory protein family, is involved in signal transduction and cell adhesion. Another extensively studied member, SIRPα, acts as an inhibitory receptor by interacting with transmembrane protein CD47, thereby controlling effector functions of the innate immune response. ^46^ Loc690948, predicted to code for Lilrb3a, is an inhibitory member of the leukocyte immunoglobulin-like receptor family. LILRBs are well documented on a broad range of immune cells, including NK cells, where they can modulate immune cell functions such as cytokine release, antibody production and antigen presentation. ^47^ They also play a role in Toll-like receptor signaling, main pathway triggered by the injection of poly I:C. ^48^

Apol11a, which codes for Apolipoprotein L, a Bcl-2 like protein, has been proposed to have a function related to cell death due to its pore-forming domain. This function is inflicted by dendritic cells after viral stimulation, while the gene is also strongly expressed in splenic DCs post stimulation with poly I:C, a reaction dependent on TLR3/TRIF, and mimicked by IFN-β. ^49^ CD72 is an inhibitory co-receptor on B cells that recognizes the RNA-containing Sm/RNP (Smith/Ribonucleoproteins as resulting from cell death) through its extracellular C-type lectin domain (CTLD). Simultaneously, it inhibits the corresponding B cell response through its intracellular immunoreceptor tyrosine-based inhibition motif (ITIM), effectively blocking TLR7-mediated activation of antibody production. ^50^ Finally, RT1-N2 belongs to the family of rat MHC class Ib molecules. Recent studies have demonstrated interactions with both inhibitory and activating Ly49 receptors, resulting in the specific expansion of allospecific NK cells. ^51^ These findings collectively underscore the central role of the spleen in modulating the behavioural conditioned immune enhancement.

With reference to Hsueh et al. ^11^ original study in mice, our investigation into IFN-related gene expression in the spleen revealed no upregulation of IFN-α or -β in the test group. We did, however, observe an upregulation of IFN-α2 gene expression in the positive group at +6h post recall. More importantly, we saw a peak at 0h in IFN-γ expression in the hypothalamus of the test group, which was also reflected by elevated IFN-gamma levels in the plasma. Consistent with the finding of Hsueh et al., we also observed elevated ACTH plasma levels in both the test group and positive control during the first six hours post recall, whereas no such elevation was observed in the negative control ( Figure 6).

For other chemokines, we saw a somewhat mixed pattern when comparing plasma levels and gene expression. While we did see a peak of GRO/KC/Cinc-1, MIP-1α, and Rantes at +6h in the plasma of both the test group and positive control, a corresponding upregulation of Cxcl1, Ccl3 and Ccl5 at gene level was only evident in the pituitary and adrenal glands of the positive control. Similarly, a plasma peak of TNF-α did show a corresponding upregulation of Tnf gene expression.

For plasma MCP-1, we noted an immediate upregulation of Ccl2 in the spleen of the positive group and three hours later in the spleen of the test group. This pattern closely resembled the expression of Cxcl10 and the corresponding plasma levels of IP-10. Specifically, we first saw an upregulation of Cxcl10 in the spleen of the positive group, followed by a similar pattern three hours later in the test group. Further, we noted three hours delayed pattern in the hypothalamus. This pattern, characterized by an initial upregulation in the spleen followed by a delayed (three hours later) upregulation in the hypothalamus, starting in the positive group and occurring immediately and three hours later in the test group, was also observed for other ISGs as Cxcl11, Gbp5, and Rsad2. This suggests that the upregulation of ISGs in the hypothalamus could be a secondary effect triggered by an initial upregulation of ISGs in the spleen.

In this context, it is noteworthy that we observed a brief yet distinct upregulation of the acetylcholine receptor Chrna3 in the spleen of test animals immediately post recall. While most of the available literature points to Chrna7 as the primary receptor for acetylcholine ^52^ in the spleen, playing a role in relaying vagus nerve signals to cytokine-producing macrophages via acetylcholine-synthesizing T cells in the spleen, ^53^ recent discoveries indicate that the Chrna7-based activation of macrophages may be mediated by a downregulation of α1,3-Fucosyltransferase Fut7. ^54^ Although we did not observe any upregulation of Chrna7 in the spleen, we did note a downregulation in Fut7 at +3h post recall in both the test group and positive control, hinting at a potential involvement of the vagal system in modulating the recall of the conditioned immune reaction.

While a recent paper by Verlinden et al. in 2019 ^55^ described catecholaminergic but not direct cholinergic innervation of the spleen, this finding does not preclude the possibility of indirect vagal innervation via postganglionic non-cholinergic fibers. This notion is further supported by a recent study by Zhang et al. ^56^ which showed that CRH neurons originating from the central nucleus of the amygdala (CeA) and PVN of the hypothalamus can stimulate the formation of splenic plasma cells via direct splenic innervation. This potential pathway, possibly involving intermediate T cells, could translate the noradrenergic signal into an acetylcholinergic message.

In summary, our data indicate that in addition to the classic HPA axis involving CRH, ACTH and cortisol release, there may be additional pathways connecting the brain and the immune system during the recall of the behaviourally conditioned immune enhancement. These pathways could include direct splenic innervation, modulating and finetuning the message via cholinergic signaling from the PVN. We further postulate that the recall of the conditioned immune response starts in the hypothalamus via a Wnt/β-catenin related pathway, involving key factors such as Otx2, Sox7/8/9/10/11, Zic1, and Fzd6 as well as monoaminergic (Slc18a2) and opioid messaging (Gpr88).

While requiring more detailed analysis, osteopontin, strongly upregulated in hypothalamus and potentially in concert with Pmch and Vip, strongly downregulated immediately post recall, also appear to play a key role during these early phases post recall – either in the communication between the hypothalamus and pituitary and/or as blood-borne immune modulators themselves. From the pituitary onward, identifying a single, specific pathway becomes challenging. We observe, however, the upregulation of steroidogenic and thermogenic genes in the pituitary and adrenal glands, respectively, indicating early hormonal activation alongside robust immunomodulation in the spleen. This coincides with a potential feedback loop involving strongly upregulated ISGs such as Cxcl9/10/11, extending from the spleen back to the brain.

### Limitations of the study

The present study has several limitations: first, not all tissue samples could be analysed due to insufficient RNA quality. Furthermore, due to the character of a pilot study, we consciously used a limited amount of animals (2) per group and time point, respectively, and only a limited number of cytokines and signaling peptides were analysed on protein level.

Another limitation considers the design of the trial groups: our negative control was built in a way that animals were exposed to camphor smell and then injected with saline on both day D0 and D2 as opposed to an alternative design with poly I:C injection on D0 and saline injection on D2. While this latter design would have had the advantage of observing residual poly I:C injection effects on D2 in the negative control, we decided against this approach as the injection procedure itself, due to its pain, might have been perceived by the animals as a conditioning stimulus and thus resulted in a partial recall. Future studies might explore a different design and/or explore injection under anesthesia to limit the injection effect.

Independently of this, we believe that the gene expression data generated in this study, in combination with plasma protein data, provide strong evidence for a role of Otx2, Vmat2, and osteopontin – in concert with opioid and monoaminergic signaling – as key efferent messengers during the recall of the conditioned immune enhancement, confirming earlier results by Hiramoto, Ghanta, and Hsueh. ^9^^–^^12^^,^^37^^,^^38^^,^^40^

## Data availability

Underlying data deposited in Zenodo.org: https://doi.org/10.5281/zenodo.7086375. ^57^

• 20220902 Affymetrix all tissues all time points red green incl p v2.xlsb (Affymetrix raw data and analysis across all tissues and time points)
• 20220803 Ct values and Affymetrix correlation v2.xlsx (qRT-PCR Ct values and corresponding Affymetrix data for 32 genes)

### Reporting guidelines

Zenodo: ARRIVE checklist for *“Differential gene expression during recall of behaviorally conditioned immune enhancement in rats: a pilot study”*, https://doi.org/10.5281/zenodo.7086375. ^57^

Data are available under the terms of the Creative Commons Attribution 4.0 International “No rights reserved” data waiver ( https://creativecommons.org/share-your-work/public-domain/cc0/)
