# Supplementary material for: Differential gene expression during recall of behaviorally conditioned immune enhancement in rats: a pilot study
Source: F1000Res. 2025 Jan 10;11:1405. Originally published 2022 Nov 30. [Version 3] doi: 10.12688/f1000research.123975.3 (PMC11745302; doi:10.12688/f1000research.123975.3)
Supplement: Peer review history

# Reviewer report: Reviewer response for version 3

**DOI:** 10.5256/f1000research.176677.r358060

**Published:** 2025-01-16

**Authors:** Federico Bermudez-Rattoni

**Affiliations:** 1 National Autonomous University of Mexico, Mexico, Mexico

I believe the authors have addressed most of the reviewers' concerns, and the paper is suitable for indexing in its current form.

Is the work clearly and accurately presented and does it cite the current literature?

Partly

If applicable, is the statistical analysis and its interpretation appropriate?

Partly

Are all the source data underlying the results available to ensure full reproducibility?

Yes

Is the study design appropriate and is the work technically sound?

Partly

Are the conclusions drawn adequately supported by the results?

Partly

Are sufficient details of methods and analysis provided to allow replication by others?

Yes

Reviewer Expertise:

Neurobiology of memory and neuroimmune communication.

I confirm that I have read this submission and believe that I have an appropriate level of expertise to confirm that it is of an acceptable scientific standard.

# Reviewer report: Reviewer response for version 2

**DOI:** 10.5256/f1000research.160426.r318706

**Published:** 2024-09-12

**Authors:** Federico Bermudez-Rattoni, Daniel Osorio Gomez

**Affiliations:** 1 National Autonomous University of Mexico, Mexico, Mexico; 2 Neurociencias, National Autonomous University of Mexico, Mexico, Mexico

The revised manuscript presents data on upregulated and downregulated genes associated with an immune-conditioned response paradigm, with analyses conducted in the HPA axis and spleen. While the authors have addressed some of the reviewers' previous comments, several concerns remain, and additional observations need to be addressed.

 The methodology states three groups of rats: test, positive, and negative. However, the rationale behind the inclusion of the positive group remains unclear. The authors apparently aimed to evaluate the naturally induced immune response by injecting Poly I: C. However, the necessity of implementing a training conditioning session on Day 0 (D0) is questionable and needs further clarification.

 The analyses involved comparisons between the Test vs. Negative and Positive vs. Negative groups. In the first comparison, the changes in gene expression are linked to differences between conditioned immune responses and untrained animals. The second comparison distinguishes between naturally induced immune responses and odor exposure. However, a crucial comparison that has been overlooked is between the Test and Positive groups. This comparison is of utmost importance, as it would reveal whether the learned immune response mirrors the naturally induced response, and it needs to be addressed in the manuscript.

 Figures 10 and 11 were not corrected; the column titles are repeated and need to reflect the evaluation time.

 In the sucrose preference test, the authors state that "…a drop in sucrose preference in both the test group and the positive control was observed after Poly I: C injection, indicating anhedonia; the reduction was less pronounced in the negative control receiving only saline injections." However, these differences are not visible in the graph provided. It is unclear how the analysis was performed. Furthermore, why did the negative group, which was only exposed to camphor odor, show a reduction in sucrose consumption? This point needs clarification.

 Regarding the temporal analyses, providing a more detailed discussion of the temporal changes in gene expression is crucial. As mentioned in a previous comment, I strongly recommend that the authors compare the Test group with the Positive group to investigate differential gene expression changes over time. This comparison is essential for understanding gene expression dynamics under these conditions. It could help determine whether some of these temporal changes are linked to conditioned versus naturally induced immune responses, offering valuable insights.

 The manuscript would also benefit from further editing and review, as some paragraphs could be more precise and accurate. For example, on page 18, the authors begin by discussing changes in dopaminergic response but abruptly shift to a statement about cholinergic activity without explaining or suggesting the underlying mechanisms involved. This disrupts the flow and coherence of the text.

 While the authors evaluated several genes, the discussion section would greatly benefit from an integrated overview of the main findings. Providing a cohesive synthesis of the results is crucial, as it would help readers understand the research and its implications, and it needs to be addressed in the manuscript.

Is the work clearly and accurately presented and does it cite the current literature?

Partly

If applicable, is the statistical analysis and its interpretation appropriate?

Partly

Are all the source data underlying the results available to ensure full reproducibility?

Yes

Is the study design appropriate and is the work technically sound?

Partly

Are the conclusions drawn adequately supported by the results?

Partly

Are sufficient details of methods and analysis provided to allow replication by others?

Yes

Reviewer Expertise:

Neurobiology of memory and neuroimmune communication.

We confirm that we have read this submission and believe that we have an appropriate level of expertise to confirm that it is of an acceptable scientific standard, however we have significant reservations, as outlined above.

# Response

**Published:** 2025-01-10

**Authors:** Markus Rueckels

**Affiliations:** Lisa-Kolk-Stiftung, Germany

**Response to reviewer’s comments**

 Reviewer’s comments: purple

*Our comments: blue italic*

**Federico Bermudez-Rattoni and Daniel Osorio Gomez**

 National Autonomous University of Mexico, Mexico, Mexico

 Reviewer’s comments: The revised manuscript presents data on upregulated and downregulated genes associated with an immune-conditioned response paradigm, with analyses conducted in the HPA axis and spleen. While the authors have addressed some of the reviewers' previous comments, several concerns remain, and additional observations need to be addressed. The methodology states three groups of rats: test, positive, and negative. However, the rationale behind the inclusion of the positive group remains unclear. The authors apparently aimed to evaluate the naturally induced immune response by injecting Poly I: C. However, the necessity of implementing a training conditioning session on Day 0 (D0) is questionable and needs further clarification.

*Author Response: Our objective was to identify genes up- and downregulated during the recall of the conditioned immune reaction. The positive group was included to exclude genes not specific for the recall pathway as e.g. Cxcl11, which is also strongly upregulated in the positive group (figure 1, page 6). In order to keep the training conditions between the groups as similar as possible, we made the positive group undergoing the same training paradigm as the test group.*

 Reviewer’s comments: The analyses involved comparisons between the Test vs. Negative and Positive vs. Negative groups. In the first comparison, the changes in gene expression are linked to differences between conditioned immune responses and untrained animals. The second comparison distinguishes between naturally induced immune responses and odor exposure. However, a crucial comparison that has been overlooked is between the Test and Positive groups. This comparison is of utmost importance, as it would reveal whether the learned immune response mirrors the naturally induced response, and it needs to be addressed in the manuscript.

*Author Response: As we aimed to identify genes, which were specific for the efferent pathway after the recall, we consciously focused on gene expression differences between the test and negative group. Genes predominantly regulated in the positive group are mentioned, too, but only if potentially indicating a different mechanism compared to the test group (page 18, “Genes such as Cxcl9, 10, 11 or Gbp5, Rsad2, and Ccl2 showed a more than 50fold upregulation in the positive control, indicating a different mechanism when comparing poly I:C re-injection to the recall of the conditioned immune reaction.”).*

 Reviewer’s comments: Figures 10 and 11 were not corrected; the column titles are repeated and need to reflect the evaluation time.

*Author Response: Figures 10 and 11 were corrected*

 Reviewer’s comments: In the sucrose preference test, the authors state that "…a drop in sucrose preference in both the test group and the positive control was observed after Poly I: C injection, indicating anhedonia; the reduction was less pronounced in the negative control receiving only saline injections." However, these differences are not visible in the graph provided. It is unclear how the analysis was performed. Furthermore, why did the negative group, which was only exposed to camphor odor, show a reduction in sucrose consumption? This point needs clarification.

*Author Response: The statement has been corrected.*

*“As shown in figure 12, we observed a drop in sucrose preference in both the test group and the positive control compared to D-1 after poly I:C injection; the reduction was much less pronounced in the negative control, receiving only saline injections. Following a return to normal on D1, we observed another drop in all three groups on D2 with further reduction in sucrose preference in all animal groups, potentially due to the repeated injection and handling stress.”*

 Reviewer’s comments: Regarding the temporal analyses, providing a more detailed discussion of the temporal changes in gene expression is crucial. As mentioned in a previous comment, I strongly recommend that the authors compare the Test group with the Positive group to investigate differential gene expression changes over time. This comparison is essential for understanding gene expression dynamics under these conditions. It could help determine whether some of these temporal changes are linked to conditioned versus naturally induced immune responses, offering valuable insights.

*Author Response: As mentioned, we focused on identifying genes, which were specific for the efferent pathway after the recall, we consciously focused on gene expression differences between test and negative group and used the positive group to exclude genes, which were not specific to the recall.*

 Reviewer’s comments: The manuscript would also benefit from further editing and review, as some paragraphs could be more precise and accurate. For example, on page 18, the authors begin by discussing changes in dopaminergic response but abruptly shift to a statement about cholinergic activity without explaining or suggesting the underlying mechanisms involved. This disrupts the flow and coherence of the text.

*Author Response: As only a few studies are available on the topic of gene expression after recall of the conditioned NK response, we compared the gene expression results with the existing literature on this topic. VMAT2 is one of the genes which stands out during the recall and is responsible for transporting monoaminergic neurotransmitters such as norepinephrine and dopamine into synaptic vesicles. Hiramoto et al. showed that reserpine, which inhibits dopamine signaling, blocks the recall of the conditioned NK response. Hsueh et al. found that both, cholinergic and serotonergic pathways, in the CNS seem to be involved during the acquisition as well as the recall of the conditioned NK cell response. Both results are in line with our finding of an involvement of VMAT2 during recall of the conditioned NK response and further corroborated by the decreased activity of dopamine receptors (Drd1, Drd2) and serotonergic receptors (Htr1f, Htr2a), observed in the hypothalamus of test animals +3h post recall.*

 Reviewer’s comments: While the authors evaluated several genes, the discussion section would greatly benefit from an integrated overview of the main findings. Providing a cohesive synthesis of the results is crucial, as it would help readers understand the research and its implications, and it needs to be addressed in the manuscript.

*Author Response: An integrated overview of the main findings is provided in the manuscript. Though the discussion is already quite long, a more detailed discussion could indeed be of interest. However, as the purpose of the study was to function as a pilot study, we focused on a few selected genes, only.*

# Reviewer report: Reviewer response for version 2

**DOI:** 10.5256/f1000research.160426.r278521

**Published:** 2024-06-27

**Authors:** Marie Jakobs

**Affiliations:** 1 Institute of Medical Psychology and Behavioral Immunobiology, University Hospital Essen, Essen, Germany

Rueckels and Picard-Mareau took an interesting and valuable approach to reveal additional pathways to the classic HPA axis, thus trying to complement the mechanisms underlying conditioned immune enhancement. However, their approach could be improved partially, allowing them to draw more meaningful conclusions. Overall, the manuscript is well-written.

 The authors state that their pilot study was designed to identify differentially expressed genes along the HPA-axis during the recall of the behaviourally conditioned immune response. Therefore, they applied the 3-day conditioning paradigm originally developed by Hsueh et al.

  However, the authors must be careful about dedicating the regulation of these differentially expressed genes to the recall of behaviourally conditioned immune responses only, since their study design is lacking a (residual effect) control group that received a poly IC injection on D0 but no poly IC treatment on D2. Looking at the gene expression of their positive control group at +48h shows that poly IC still influences the regulation of certain genes on D2, when conditioning is retrieved. This fact should be mentioned in the limitations section.

 In the results section, the authors present a vast amount of interesting data. However, in some cases the recorded results do not seem to be consistent (with the corresponding figures).

  On page 7, the authors wrote that they “identified 12 and 10 upregulated transcripts in the hypothalamus and the pituitary, respectively (Figure 1).”, whereas on the same page in the hypothalamus chapter they wrote that “in the hypothalamus, a total of 11 transcripts, detected by 12 probes, were upregulated [...]”. In addition, Figure 1 shows 11 (not 10) upregulated transcripts in the pituitary for test vs. negative control.

 Moreover, looking at Figure 1, for the reader it is not easy to understand, why the authors concluded that there is “significant upregulation” for Slc18a2 (1.3) in the test group vs. negative control, but “only a trend towards significance” for Spp1 (1.3) in the test group vs. negative control in the hypothalamus. Probably, the authors rather compared test-neg vs. pos-neg than test group vs. negative control? Additional information in the figure legends or rephrasing might be helpful/necessary.

 The labeling of the individual columns in Figure 10 and Figure 11 seems to be incorrect. Each caption says “test-neg” instead of “0h, +3h, +6h, ...”.

  Overall, the results section would be easier to read, if the figures appeared in chronological order.

  The sucrose preference test (SPT) was conducted on D-7 to D-1 as well as on D0 to D3. Drinking bottles were presented for 24h. To better interpret the results, a specification of the number of animals tested on each study day would be helpful, since euthanasia of the animals already started on D2 at various timepoints.

Is the work clearly and accurately presented and does it cite the current literature?

Partly

If applicable, is the statistical analysis and its interpretation appropriate?

I cannot comment. A qualified statistician is required.

Are all the source data underlying the results available to ensure full reproducibility?

Yes

Is the study design appropriate and is the work technically sound?

Partly

Are the conclusions drawn adequately supported by the results?

Partly

Are sufficient details of methods and analysis provided to allow replication by others?

Yes

Reviewer Expertise:

Behavioural conditioning of immune responses.

I confirm that I have read this submission and believe that I have an appropriate level of expertise to confirm that it is of an acceptable scientific standard, however I have significant reservations, as outlined above.

# Response

**Published:** 2025-01-10

**Authors:** Markus Rueckels

**Affiliations:** Lisa-Kolk-Stiftung, Germany

**Response to reviewer’s comments**

 Reviewer’s comments: purple

*Our comments: blue italic*

**Marie Jakobs**

 Institut of Medical Psychology and Behavioral Immunobiology, University of Duisburg-Essen, Essen, Germany

 However, the authors must be careful about dedicating the regulation of these

 differentially expressed genes to the recall of behaviourally conditioned immune responses

 only, since their study design is lacking a (residual effect) control group that received a poly

 IC injection on D0 but no poly IC treatment on D2. Looking at the gene expression of their

 positive control group at +48h shows that poly IC still influences the regulation of certain

 genes on D2, when conditioning is retrieved. This fact should be mentioned in the

 limitations section.

*The limitations section was expanded accordingly.*

*“Another limitation considers the design of the trial groups: our negative control was built in a way that animals were exposed to camphor smell and then injected with saline on both, day D0 and D2, as opposed to an alternative design with poly I:C injection on D0 and saline injection on D2. While this latter design would have had the advantage of observing residual poly I:C injection effects on D2 in the negative control, we decided against this approach as the injection procedure itself, due to its pain, might have been perceived by the animals as a conditioning stimulus and thus resulted in a partial recall. Future studies might explore a different design and/or explore injection under anesthesia to limit the injection effect.”*

 In the results section, the authors present a vast amount of interesting data. However, in

 some cases the recorded results do not seem to be consistent (with the corresponding

 figures). On page 7, the authors wrote that they “identified 12 and 10 upregulated transcripts in the hypothalamus and the pituitary, respectively (Figure 1).”, whereas on the same page in the hypothalamus chapter they wrote that “in the hypothalamus, a total of 11 transcripts, detected by 12 probes, were upregulated [...]”. In addition, Figure 1 shows 11 (not 10) upregulated transcripts in the pituitary for test vs. negative control.

*The numbers were corrected in the results section. Eleven transcripts were upregulated.*

*“Leveraging this approach to identify major upregulated genes in test vs. negative control across all tissues with a >2fold average regulation across all time points, we identified 11 upregulated transcripts in both the hypothalamus and the pituitary (* *Figure 1* *).”*

 Moreover, looking at Figure 1, for the reader it is not easy to understand, why the authors

 concluded that there is “significant upregulation” for Slc18a2 (1.3) in the test group vs.

 negative control, but “only a trend towards significance” for Spp1 (1.3) in the test group vs.

 negative control in the hypothalamus. Probably, the authors rather compared test-neg vs.

 pos-neg than test group vs. negative control? Additional information in the figure legends

 or rephrasing might be helpful/necessary.

*Test for significance was calculated in an early stage of the study to identify highly differentially expressed genes using absolute expression values for all time points of test vs all time points of negative (<0.05). Since that approach could not be expanded to all genes lacking sufficient data, we decided to remove significance statements.*

*The results section was modified accordingly:*

*“..Spp1, which codes for secreted immune modulator osteopontin (OPN), and Slc18a2, which codes for vesicular monoamine transporter 2 (VMAT2), demonstrated the strongest upregulation in the test group vs. negative control, without a comparable upregulation in the positive vs. negative control. Likewise, Otx2, which codes for orthodenticle homeobox 2 and was detected by two independent probes, showed a strong upregulation in the test group vs. negative control without a strong upregulation in positive group vs. negative control as e.g. observed for Hapln4 or Cxcl11 (* *Figure 1* *).*

*…On the other hand, Fa2h, Tspan18, Sh2b2 and Hapln4 and Cxcl11 as already mentioned, either..”*

 The labeling of the individual columns in Figure 10 and Figure 11 seems to be incorrect.

 Each caption says “test-neg” instead of “0h, +3h, +6h, ...”.

 Overall, the results section would be easier to read, if the figures appeared in chronological

 Order

*The labeling was corrected (see Fig 10 and Fig 11).*

 The sucrose preference test (SPT) was conducted on D-7 to D-1 as well as on D0 to D3.

 Drinking bottles were presented for 24h. To better interpret the results, a specification of

 the number of animals tested on each study day would be helpful, since euthanasia of the

 animals already started on D2 at various timepoints.

*The number of animals was indicated in the methods section accordingly. 30 animals were analyzed per day except day 2 (12 animals) and day 3 (6 animals).*

*“Except for days D2 and D3, all 30 animals were analyzed; due to animal sacrificing post-treatment on D2, only 12 animals were analyzed on D2 and 6 animals on D3.”*

# Reviewer report: Reviewer response for version 1

**DOI:** 10.5256/f1000research.136138.r174282

**Published:** 2023-06-20

**Authors:** Daniele Suzete Persike de Oliveira

**Affiliations:** 1 Department of Medicinal Chemistry, College of Pharmacy, University of Duhok, Duhok, Iraq

Reading this article was very useful for me, since it addresses a very interesting and still not well understood topic. 

 I understand that the article was well written and presented in a clear manner. I would only have two comments:

1. Despite being a pilot study, the total number of animals used per group seems to be quite small (n=5).
  A clarification on that would be appreciated since the Table 1 mentions 5 animals/group, but the descriptive text below it explains that the animals from each group were later divided into 0h (n=2), +3h (n=2), +6h (n=2), +24h (n=2) and +48h (n=2) making a total of 10 animals/group.
2. I believe it would be interesting if the author addressed the "placebo response" in the discussion. Current placebo research postulates that conditioning processes are one of the major mechanisms of the placebo response and I believe it would be something interesting to be addressed as it might be part of the results observed. See the article: Vits *et al. *(2011 ^1^).

Is the work clearly and accurately presented and does it cite the current literature?

Yes

If applicable, is the statistical analysis and its interpretation appropriate?

Yes

Are all the source data underlying the results available to ensure full reproducibility?

Yes

Is the study design appropriate and is the work technically sound?

Yes

Are the conclusions drawn adequately supported by the results?

Yes

Are sufficient details of methods and analysis provided to allow replication by others?

Yes

Reviewer Expertise:

Neuroscience.

I confirm that I have read this submission and believe that I have an appropriate level of expertise to confirm that it is of an acceptable scientific standard, however I have significant reservations, as outlined above.

## References

[1] : Behavioural conditioning as the mediator of placebo responses in the immune system. *Philos Trans R Soc Lond B Biol Sci* .2011;366(1572) : 10.1098/rstb.2010.0392 1799-807 10.1098/rstb.2010.0392 21576137 PMC3130401

# Reviewer report: Reviewer response for version 1

**DOI:** 10.5256/f1000research.136138.r173302

**Published:** 2023-06-15

**Authors:** Manfred Schedlowski, Laura Lückemann

**Affiliations:** 1 University of Duisburg-Essen, Essen, Germany; 2 Institut of Medical Psychology and Behavioral Immunobiology, University of Duisburg-Essen, Essen, Germany

The present approach taken by Rueckels *et al.* investigates in biochemical pathways generating conditioned immune enhancement. The authors showed, upregulation of genes related to Wnt/β-catenin signaling (Otx2, Spp1, Fzd6, Zic1), monoaminergic transporter Slc18a2, and opioid-inhibitory G-protein Gpr88 in the hypothalamus as well as downregulation of dopaminergic receptors, vasoactive intestinal peptide (Vip), and pro-melanin-concentrating hormone (Pmch). In the pituitary, they report upregulation of steroid synthesis and neurotransmission genes (GABAergic, cholinergic, opioid). Peripheral data from spleen showed upregulation of immunomodulatory genes, chemo-/cytokines, and neurotransmission genes (glutamatergic/cholinergic). Plasma analyses confirmed increased chemokine and ACTH levels. Based on their results the authors suggest the possibility of additional pathways, such as the cholinergic anti-inflammatory pathway (CAIP), which could serve as a connection between the brain and immune system. This pathway may play a role in regulating and finely adjusting the communication between these two systems.

 The authors have taken an interesting approach, revealing the hypothesis of an alternative pathways in addition to the classic HPA axis. However, there are a number of issues which have to be addressed before the manuscript can be indexed.

  

- The overall poor quality of language (huge amount of typos and grammar errors) makes the manuscript difficult to read and unfortunately suggests sloppiness in terms of preparation. The manuscript needs to be carefully checked for grammar and typos (by a native English speaker).
- There is some inconsistency between the abstract and the discussion section. Quality of language changed throughout the manuscript. Please comment on this.
- Even though the methodologies used in these studies appear to be appropriate, there are some issues that need discussion. All experiments are missing a “negative control group”, in order to exclude possible residual effects of PolyI:C in which animals are injected with Poly I:C during the learning phase and receive no treatment during recall D2. These results would confirm that the measured ”conditioning effects” are not only “residual effects” of Poly I:C treatment, since the experimental design allows only one day retention interval between the learning and testing phases.
- *Page 1*: ”connecting brain and immune system, modulating and finetuning communication between brain and immune system” redundant; please rephrase.
- *Page 3: line 1-5 *“Pavlovian or classical conditioning is a phenomenon of associative learning”, it is not clear why you make a difference between both conditioning paradigms. The reader might get confused, because both expressions describe the same paradigm.
- *Page 3 line 30-31: *“48h later, animals in the test group were re-exposed to camphor smell, only, to recall a behaviorally conditioned immune response in rats while animals in a positive control group receive a re-injection of poly I:C and animals in a negative control group receive smell conditioning, only, but no poly I:C re-injection”. This sentence does not make sense. Please rephrase.
- Methodology/terminology - animals in the negative control group did not receive smell conditioning (neither in the learning phase (saline injection) nor in the recall phase (re-exposure to camphor smell)).
- Page 3, line 11: terminology - what does "work paradigms“ mean?
- Page 3 Table 1: An additional timeline would be very helpful for the reader to understand the complex experiments.
- *Page 4 line 1*: Please provide literature for this poly I:C dose (Sigma #P1530; ip; 1 mg/kg)
- *Page 4 , line 8-9:* Consistent terminology – "for the isolation of […] serum to perform cytokine/chemokine array analysis“ throughout the rest of the paper, the term "plasma“ is used.
- *Page 4 line 16:* Consistent terminology – "days D-7 to D-1 […] as well as days D0 – D3.“
- *Page 4, line 18*: Comprehensibility - "Rats were presented with two drinking bottles for 24 h. One filled with plain drinking water and one filled with 2% (w/v) sucrose solution.“
- *Page 4 line 18:* Methodology – "rats were housed in groups of two“. How was the SPT conducted to assess individual fluid consumption?
- *Page 4, line 38-39*: Poor and vague language – "Since we hypothesized that the conditioned response evoked during recall was mediated by the hypothalamus, […]“.
- *Page 5, line 8*: blank space between number and unit of measurement – "1 µl of each primer“ "1 µl sterile water“.
- *Page 5, line 22:* Consistent terminology - "All statistical analyses were conducetd“ (consistent with heading).
- All figures are of poor quality/resolution; please do not use screenshots from Excel or other programs for a scientific publication. For example: Figure 1-5 : Figure legend is missing, the figure is blurry, words are cut off, no proper headings describing the columns. Does each column represent one tissue? Tables, no figures.
- Figure 6 & 7; the reader has to guess the numbers and Abbreviations (Poor quality) Please correct. Figure 11: poor figure – figure legend is missing, the figure is blurry, words are cut off, no proper headings describing the columns. What do the grey columns mean?
- *Page 5, line 37*: Terminology - "test vs. negative control ('test‘) (the term "test“ is already assigned to the conditioned experimental group. It is confusing to use this term again to describe the comparison between test vs. negative control).
- *Page 5, line 43*: Terminology - "positive vs. negative control ('positive‘) (the term "positive“ is already assigned to the positive group. It is confusing to use this term again to describe the comparison between positive vs. negative control)
- *Page 6, line 1-5*: Sentences throughout the paper are too long, complicated, including too many commas and are therefore incomprehensible.
- Page 7, line 6-8: Sentences throughout the paper are too long, complicated, including too many commas and are therefore incomprehensible – "in test. However, they were either strongly upregulated in positive or did not reach significance when comparing gene expression in test vs. negative control. While […]“
- *Page 8, line 2:* Figures do not appear in chronological order throughout the text.
- *Page 11, line 20*: consistent terminology, poor language – "in test was only observed +3h post recall“.
- *Page 11, Table 2*: Comprehensibility - "including individual correlation per gene and correlation across all data points.“ Are there two different correlations? Which correlation is depicted in the table?
- *Page 13, Figure 6*: Poor figure – figure legend is missing, the figure is blurry, consistent terminology, alpha, gamma, etc. consistently depicted as symbols or written words.
- *Page 12, line 2*: Comprehensibility – "immediately post recall“ What does immediately mean? 0h?
- *Page 14, Figure 8*: Comprehensibility – the connection between "Viral proteins and –nucleic acids; Poly I:C“ and the rest of the figure is missing, if the figure is intended to illustrate the pathways involved during recall of behaviorally conditioned immune response, it would make more sense to depict camphor smell instead of Poly I:C, figure legend is missing
- *Page 14, line 1*: Methodology/comprehensibility – "in negative control with saline injection only.“ ("only“ is misleading, because animals received a saline injection and camphor smell).
- *Page 15, Figure 10*: Poor figure – figure legend is missing, the figure is blurry, words are cut off, no proper headings describing the columns. What doo the grey columns mean?
- *Page 15, Figure 11:* Poor figure – figure legend is missing, the figure is blurry, words are cut off, no proper headings describing the columns. What does the grey columns mean?
- *Page 15, Figure 12*: groups do not really differ on D0.
- *Page 15, line 3*: Consistent terminology – "on D0 and D2, respectively)“.
- *Page 17, line 19:* Repetition/redundancy – "further“.

Is the work clearly and accurately presented and does it cite the current literature?

No

If applicable, is the statistical analysis and its interpretation appropriate?

Partly

Are all the source data underlying the results available to ensure full reproducibility?

Partly

Is the study design appropriate and is the work technically sound?

Partly

Are the conclusions drawn adequately supported by the results?

Partly

Are sufficient details of methods and analysis provided to allow replication by others?

Yes

Reviewer Expertise:

Behavioral Immunobiology

We confirm that we have read this submission and believe that we have an appropriate level of expertise to confirm that it is of an acceptable scientific standard, however we have significant reservations, as outlined above.

# Response

**Published:** 2023-12-19

**Authors:** Markus Rueckels

**Affiliations:** Lisa-Kolk-Stiftung, Germany

**Response to reviewer’s comments**

 Reviewers comments: purple

 Our comments: black

*Revised manuscript text of version 2 where applicable: blue/Italic*

**Manfred Schedlowski**

 University of Duisburg-Essen, Essen, Germany

**Laura Lückemann**

 Institut of Medical Psychology and Behavioral Immunobiology, University of Duisburg-Essen, Essen, Germany

 The present approach taken by Rueckels *et al. *investigates in biochemical pathways generating conditioned immune enhancement. The authors showed, upregulation of genes related to Wnt/β- catenin signaling (Otx2, Spp1, Fzd6, Zic1), monoaminergic transporter Slc18a2, and opioid- inhibitory G-protein Gpr88 in the hypothalamus as well as downregulation of dopaminergic receptors, vasoactive intestinal peptide (Vip), and pro-melanin-concentrating hormone (Pmch). In the pituitary, they report upregulation of steroid synthesis and neurotransmission genes (GABAergic, cholinergic, opioid). Peripheral data from spleen showed upregulation of immunomodulatory genes, chemo-/cytokines, and neurotransmission genes (glutamatergic/cholinergic). Plasma analyses confirmed increased chemokine and ACTH levels. Based on their results the authors suggest the possibility of additional pathways, such as the cholinergic anti-inflammatory pathway (CAIP), which could serve as a connection between the brain and immune system. This pathway may play a role in regulating and finely adjusting the communication between these two systems

 The authors have taken an interesting approach, revealing the hypothesis of an alternative pathways in addition to the classic HPA axis. However, there are a number of issues which have to be addressed before the manuscript can be indexed.

- The overall poor quality of language (huge amount of typos and grammar errors) makes the manuscript difficult to read and unfortunately suggests sloppiness in terms of preparation. The manuscript needs to be carefully checked for grammar and typos (by a native English speaker)

The manuscript was carefully reviewed and revised by a native English speaker improving language and expression. Typos were carefully removed.

  

- There is some inconsistency between the abstract and the discussion section. Quality of language changed throughout the manuscript. Please comment on this.

The manuscript consists of parts independently written by the two authors. Language and expression were carefully revised and aligned.

  

- Even though the methodologies used in these studies appear to be appropriate, there are some issues that need discussion. All experiments are missing a “negative control group”, in order to exclude possible residual effects of PolyI:C in which animals are injected with Poly I:C during the learning phase and receive no treatment during recall D2. These results would confirm that the measured ”conditioning effects” are not only “residual effects” of Poly I:C treatment, since the experimental design allows only one day retention interval between the learning and testing phases.

The experiment could have been conducted in two ways regarding the design of the negative control: first, camphor smell and poly I:C on day 0 and saline, only, but no smell on day 2. Secondly, camphor smell and saline at both day 0 and day 2. The second option was chosen to exclude a conditioning effect of the injection procedure itself, potentially recalled through saline injection on day 2.

  

- *Page 1*: ”connecting brain and immune system, modulating and finetuning communication between brain and immune system” redundant; please rephrase.

The sentence was modified, new sentence:

*Our data set indicates that in addition to the classic HPA axis there could be additional pathways as e.g. the cholinergic anti-inflammatory pathway (CAIP), modulating and fine tuning communication between brain and immune system.*

  

- *Page 3: line 1-5 *“Pavlovian or classical conditioning is a phenomenon of associative learning”, it is not clear why you make a difference between both conditioning paradigms. The reader might get confused, because both expressions describe the same paradigm.

The sentence was modified, new sentence:

*Pavlovian, sometimes also called classical conditioning, is a phenomenon of associative learning,*

  

- *Page 3 line 30-31: *“48h later, animals in the test group were re-exposed to camphor smell, only, to recall a behaviorally conditioned immune response in rats while animals in a positive control group receive a re-injection of poly I:C and animals in a negative control group receive smell conditioning, only, but no poly I:C re-injection”. This sentence does not make sense. Please rephrase.

The sentence was modified, new sentence:

*Following that paradigm, all animals were first exposed to camphor smell and animals in the test and positive control group then injected with polyinosinic:polycytidylic acid (poly I:C), simulating a viral infection and inducing so-called sickness behavior, an animal model for depression-like behavior in rodents, while animals in the negative group were injected with saline. 48h later, animals in the test group were re-exposed to camphor smell, only, to recall the a behaviorally conditioned immune response in rats while animals in the positive control group receive a re-injection of poly I:C, only. Animals in the negative control group received smell re-exposure and an injection of saline.*

  

- Methodology/terminology - animals in the negative control group did not receive smell conditioning (neither in the learning phase (saline injection) nor in the recall phase (re- exposure to camphor smell)).

The experiment could have been conducted in two ways regarding the design of the negative control: first, camphor smell and poly I:C on day 0 and saline only day 2. Secondly, camphor smell and saline at day 0 and day 2. The second option was chosen to exclude all potential impact of the camphor smell alone.

  

- Page 3, line 11: terminology - what does "work paradigms“ mean?

The sentence was modified, new sentence:

*by different researchers all over the world and current knowledge about this*

*phenomenon including potential pathways and experimental paradigms employed have been comprehensively summarized in a recent review by Hadamitzky et al*

  

- Page 3 Table 1: An additional timeline would be very helpful for the reader to understand

the complex experiments.

 A illustrative timeline was added below Table 1

  

- *Page 4 line 1*: Please provide literature for this poly I:C dose (Sigma #P1530; ip; 1 mg/kg)

The reference below was added:

*Behav Pharmacol 2010 Jul;21(4):369-73. doi: 10.1097/FBP.0b013e32833c7ce5.*

 Synthetic double-stranded RNA polyinosinic-polycytidylic acid augments morphine-induced conditioned place preference in rats

Lei Zhang ^1^ , Suqing Chen, Hongyu Liu, Lin Lu, Haifeng Zhai

  

- *Page 4 , line 8-9: *Consistent terminology – "for the isolation of [...] serum to perform cytokine/chemokine array analysis“ throughout the rest of the paper, the term "plasma“ is used.

The sentence was corrected to plasma

**Page 4 line 16**

 The sentence was modified:

 The SPT was carried out on D-7 to D-1 (run-in phase for baseline data) as well as days D0 - D3.

  

- *page 4, line 18*: Comprehensibility - "Rats were presented with two drinking bottles for 24 h.

One filled with plain drinking water and one filled with 2% (w/v) sucrose solution.“

**The sentence was modified accordingly:**

 Rats were presented with two drinking bottles, one with plain drinking water and one with 2% (w/v) sucrose solution for 24 h. The ratio of sweetened vs. plain water consumed during this time period was calculated, and results were expressed as %sucrose preference.

  

- *Page 4 line 18: *Methodology – "rats were housed in groups of two“. How was the SPT conducted to assess individual fluid consumption?

Average values per cage were assessed. The assessment of individual values was not conducted.

  

- *Page 4, line 38-39*: Poor and vague language – "Since we hypothesized that the conditioned response evoked during recall was mediated by the hypothalamus, [...]“.

The sentence was modified:

*As we hypothesized that the message post recall originated from hypothalamus, we additionally leveraged QIAGEN Ingenuity Pathway Analysis (QIAGEN IPA) for a more detailed analysis of hypothalamic genes. All genes identified with avg. regulation >2fold in the hypothalamus of test animals were selected and subcellular network analysis was derived using IPA graphic interface.*

  

- *Page 5, line 8*: blank space between number and unit of measurement – "1 μl of each primer“ "1 μl sterile water“.

Corrections were made for all units of measurement accordingly throughout the manuscript.

  

- All figures are of poor quality/resolution; please do not use screenshots from Excel or other programs for a scientific publication. For example: Figure 1-5 : Figure legend is missing, the figure is blurry, words are cut off, no proper headings describing the columns. Does each column represent one tissue? Tables, no figures.

All figures including headings, legend and resolution of the figures were improved.

  

- *Page 5, line 22: *Consistent terminology - "All statistical analyses were conducted“ (consistent with heading).

The sentence was modified accordingly:

*All statistical analyses were conducted using RMA gene expression data for all available time points for test, positive and negative control, respectively, applying a two-tailed, unpaired, homoscedastic Student t-test (Microsoft® Excel® for Microsoft 365 MSO, version 2022) *

- Figure 6 & 7; the reader has to guess the numbers and Abbreviations (Poor quality) Please correct. Figure 11: poor figure – figure legend is missing, the figure is blurry, words are cut off, no proper headings describing the columns. What do the grey columns mean?

We sent high resolution graphics to the editorial office to improve the quality of the figure resolution.

 The grey columns represent samples which could not be analyzed due to insufficient RNA quality.

  

- *Page 5, line 37*: Terminology - "test vs. negative control ('test‘) (the term "test“ is already assigned to the conditioned experimental group. It is confusing to use this term again to describe the comparison between test vs. negative control).

The text was modified accordingly:

*Leveraging this approach to identify major upregulated genes in test vs. negative control across all tissues with a >2-fold average regulation across all time points, we identified 12 and 10 upregulated transcripts in hypothalamus and pituitary, respectively (Figure 1). *

- *Page 5, line 43*: Terminology - "positive vs. negative control ('positive‘) (the term "positive“ is already assigned to the positive group. It is confusing to use this term again to describe the comparison between positive vs. negative control)

The text was modified accordingly:

*We also excluded genes with a stronger upregulation in positive vs. negative control than test except when also showing a strong regulation across multiple other tissues as e.g. Cxcl11 or Cxcl13 and applied a similar approach to focus on the most promising candidate genes for downregulation (Figure 2).*

- *Page 6, line 1-5*: Sentences throughout the paper are too long, complicated, including too many commas and are therefore incomprehensible.

The manuscript was reviewed and revised accordingly:

*In addition to this, we focused at up- and downregulation of neurotransmitter-associated genes such as receptors, transporters, and solute carriers, potentially involved in dopaminergic, serotonergic, opioid, cholinergic, glutamatergic, or GABAergic neurotransmission. We then selected for strong regulation across multiple tissues, even if not crossing an average >2fold regulation across all timepoints analyzed and limited the time frame for this analysis to the first 6h post recall unless otherwise mentioned.*

  

- Page 7, line 6-8: Sentences throughout the paper are too long, complicated, including too many commas and are therefore incomprehensible – "in test. However, they were either strongly upregulated in positive or did not reach significance when comparing gene expression in test vs. negative control. While [...]“

The manuscript was reviewed and revised accordingly. 

*In addition to Slc18a2, Otx2 and Spp1, 8 additional genes were found upregulated >2fold in hypothalamus. Of these, only Fzd6 and Zic1 showed a strong predominant upregulation in test vs. negative control, while Hapln4, Cxcl11, Fa2h, Tspan18, and Sh2b2 either showed a less strong regulation or were equally or even stronger regulated in positive vs. negative control.*

- *Page 8, line 2: *Figures do not appear in chronological order throughout the text.

Figures are inserted in the text by the editorial office

-  
*Page 11, line 20*: consistent terminology, poor language – "in test was only observed +3h post recall“.

The text was modified accordingly

*And similar to hypothalamus, also in spleen we saw a pattern of immediate regulation in positive vs. negative control of e.g. opioid-related transcripts as Pcsk1 or Gpr88, while the same upregulation in test vs. negative control was only observed 3h later.*

  

- ○ *Page 11, Table 2*: Comprehensibility - "including individual correlation per gene and correlation across all data points.“ Are there two different correlations? Which correlation is depicted in the table?

The text was modified accordingly

*Table 2. List of 32 genes used for validation of Affymetrix rat genome chip data by qRT-PCR including individual correlation per gene and total correlation across all genes. *

-  
*Page 13, Figure 6*: Poor figure – figure legend is missing, the figure is blurry, consistent terminology, alpha, gamma, etc. consistently depicted as symbols or written words.

Graphics resolution was improved. The use of abbreviations was changed to a uniform expression.               

 The text in figure 6 was modified accordingly:

*Figure 6. Heat map showing levels of IFN-γ, ACTH, GRO/KC/CINC-1, MIP-1α, RANTES, TNFα, MCP-1 and IP-10 in the plasma of test, positive and negative control animals.*

  

-  
*Page 12, line 2*: Comprehensibility – "immediately post recall“ What does immediately mean? 0h?

Yes, immediately means 0 h.

- ○ *Page 14, Figure 8*: Comprehensibility – the connection between "Viral proteins and –nucleic acids; Poly I:C“ and the rest of the figure is missing, if the figure is intended to illustrate the pathways involved during recall of behaviorally conditioned immune response, it would make more sense to depict camphor smell instead of Poly I:C, figure legend is missing

Text for figure 8 was modified:

*Figure 8. Illustration showing the possible afferent (poly I:C, mimicking a viral infection) and  efferent (gene regulation in hypothalamus, pituitary, adrenal and spleen) pathways involved in the immune regulation during recall of the behaviorally conditioned immune response.*

- *Page 14, line 1*: Methodology/comprehensibility – "in negative control with saline injection only.“ ("only“ is misleading, because animals received a saline injection and camphor smell).

“, only” was removed

-  
*Page 15, Figure 10*: Poor figure – figure legend is missing, the figure is blurry, words are cut off, no proper headings describing the columns. What do the grey columns mean?

All figures including headings, description of columns and resolution of the figures were improved.

-  
*Page 15, Figure 11: *Poor figure – figure legend is missing, the figure is blurry, words are cut off, no proper headings describing the columns. What does the grey columns mean?

All figures including headings, description of columns and resolution of the figures were improved.

- *Page 15, Figure 12*: groups do not really differ on D0.

Test and positive control showed an average reduction of 10.6% and 13.5% sucrose preference, respectively, versus 2.5% reduction in negative control on DO compared to D-1. As none of the groups reached significance (p < 0.05), we didn’t show any data

  

- *Page 15, line 3*: Consistent terminology – "on D0 and D2, respectively)“.

All figures including headings, legends, description of columns and resolution of the figures were improved.

- *Page 17, line 19: *Repetition/redundancy – "further“.

The sentence was modified accordingly:

*Further to this hypothesis, Trinh et al. in 2020 demonstrated that stress, induced by systemic poly I:C injection, can increase plasma OPN, triggering the release of ACTH.*

  

- * *
*4, line 38-39*: Poor and vague language – "Since we hypothesized that the conditioned response evoked during recall was mediated by the hypothalamus, […]“.
- *Page 5, line 8*: blank space between number and unit of measurement – "1 µl of each primer“ "1 µl sterile water“.
- *Page 5, line 22:* Consistent terminology - "All statistical analyses were conducetd“ (consistent with heading).
- All figures are of poor quality/resolution; please do not use screenshots from Excel or other programs for a scientific publication. For example: Figure 1-5 : Figure legend is missing, the figure is blurry, words are cut off, no proper headings describing the columns. Does each column represent one tissue? Tables, no figures.
- Figure 6 & 7; the reader has to guess the numbers and Abbreviations (Poor quality) Please correct. Figure 11: poor figure – figure legend is missing, the figure is blurry, words are cut off, no proper headings describing the columns. What do the grey columns mean?
- *Page 5, line 37*: Terminology - "test vs. negative control ('test‘) (the term "test“ is already assigned to the conditioned experimental group. It is confusing to use this term again to describe the comparison between test vs. negative control).
- *Page 5, line 43*: Terminology - "positive vs. negative control ('positive‘) (the term "positive“ is already assigned to the positive group. It is confusing to use this term again to describe the comparison between positive vs. negative control)
- *Page 6, line 1-5*: Sentences throughout the paper are too long, complicated, including too many commas and are therefore incomprehensible.
- Page 7, line 6-8: Sentences throughout the paper are too long, complicated, including too many commas and are therefore incomprehensible – "in test. However, they were either strongly upregulated in positive or did not reach significance when comparing gene expression in test vs. negative control. While […]“
- *Page 8, line 2:* Figures do not appear in chronological order throughout the text.
- *Page 11, line 20*: consistent terminology, poor language – "in test was only observed +3h post recall“.
- *Page 11, Table 2*: Comprehensibility - "including individual correlation per gene and correlation across all data points.“ Are there two different correlations? Which correlation is depicted in the table?
- *Page 13, Figure 6*: Poor figure – figure legend is missing, the figure is blurry, consistent terminology, alpha, gamma, etc. consistently depicted as symbols or written words.
- *Page 12, line 2*: Comprehensibility – "immediately post recall“ What does immediately mean? 0h?
- *Page 14, Figure 8*: Comprehensibility – the connection between "Viral proteins and –nucleic acids; Poly I:C“ and the rest of the figure is missing, if the figure is intended to illustrate the pathways involved during recall of behaviorally conditioned immune response, it would make more sense to depict camphor smell instead of Poly I:C, figure legend is missing
- *Page 14, line 1*: Methodology/comprehensibility – "in negative control with saline injection only.“ ("only“ is misleading, because animals received a saline injection and camphor smell).
- *Page 15, Figure 10*: Poor figure – figure legend is missing, the figure is blurry, words are cut off, no proper headings describing the columns. What doo the grey columns mean?
- *Page 15, Figure 11:* Poor figure – figure legend is missing, the figure is blurry, words are cut off, no proper headings describing the columns. What does the grey columns mean?
- *Page 15, Figure 12*: groups do not really differ on D0.
- *Page 15, line 3*: Consistent terminology – "on D0 and D2, respectively)“.
- *Page 17, line 19:* Repetition/redundancy – "further“.

- **Is the work clearly and accurately presented and does it cite the current literature?**

No

- **Is the study design appropriate and is the work technically sound?**

Partly

- **Are sufficient details of methods and analysis provided to allow replication by others?**

Yes

- **If applicable, is the statistical analysis and its interpretation appropriate?**

Partly

- **Are all the source data underlying the results available to ensure full reproducibility?**

Partly

- **Are the conclusions drawn adequately supported by the results?**

Partly
